# The ins and outs of vanillyl alcohol oxidase: Identification of ligand migration paths

**DOI:** 10.1371/journal.pcbi.1005787

**Published:** 2017-10-06

**Authors:** Gudrun Gygli, Maria Fátima Lucas, Victor Guallar, Willem J. H. van Berkel

**Affiliations:** 1 Laboratory of Biochemistry, Wageningen University & Research, Stippeneng 4, WE Wageningen, The Netherlands; 2 Joint BSC-IRB Research Program in Computational Biology, Barcelona Supercomputing Center, Jordi Girona 29, Barcelona, Spain; 3 Institució Catalana de Recerca i Estudis Avançats (ICREA), Passeig Lluís Companys 23, Barcelona, Spain; UNC Charlotte, UNITED STATES

## Abstract

Vanillyl alcohol oxidase (VAO) is a homo-octameric flavoenzyme belonging to the VAO/PCMH family. Each VAO subunit consists of two domains, the FAD-binding and the cap domain. VAO catalyses, among other reactions, the two-step conversion of *p-*creosol (2-methoxy-4-methylphenol) to vanillin (4-hydroxy-3-methoxybenzaldehyde). To elucidate how different ligands enter and exit the secluded active site, Monte Carlo based simulations have been performed. One entry/exit path via the subunit interface and two additional exit paths have been identified for phenolic ligands, all leading to the *si* side of FAD. We argue that the entry/exit path is the most probable route for these ligands. A fourth path leading to the *re* side of FAD has been found for the co-ligands dioxygen and hydrogen peroxide. Based on binding energies and on the behaviour of ligands in these four paths, we propose a sequence of events for ligand and co-ligand migration during catalysis. We have also identified two residues, His466 and Tyr503, which could act as concierges of the active site for phenolic ligands, as well as two other residues, Tyr51 and Tyr408, which could act as a gateway to the *re* side of FAD for dioxygen. Most of the residues in the four paths are also present in VAO’s closest relatives, eugenol oxidase and *p*-cresol methylhydroxylase. Key path residues show movements in our simulations that correspond well to conformations observed in crystal structures of these enzymes. Preservation of other path residues can be linked to the electron acceptor specificity and oligomerisation state of the three enzymes. This study is the first comprehensive overview of ligand and co-ligand migration in a member of the VAO/PCMH family, and provides a proof of concept for the use of an unbiased method to sample this process.

## Author summary

Enzymes are bionanomachines, which speed up chemical reactions in organisms. To understand how they achieve that, we need to study their mechanisms. Computational enzymology can show us what happens in the enzyme’s active site during a reaction. But molecules need first to reach the active site before a reaction can start. The process of substrate entry and product exit to the active site is often neglected when studying enzymes. However, these two events are of fundamental importance to the proper functioning of any enzyme. We are interested in these dynamic processes to complete our understanding of the mode of action of enzymes. In our work, we have studied substrate and product migration in vanillyl alcohol oxidase. This enzyme can produce the flavour vanillin and enantiopure alcohols, but also catalyses other reactions. The named products are of interest to the flavour- and fine-chemical industries.

## Introduction

Vanillyl alcohol oxidase (VAO) is a flavoenzyme involved in the mineralisation of aromatic compounds in the soil fungus *Penicillium simplicissimum*. VAO is active with a wide range of *para*-substituted phenolic compounds, but received its name because the enzyme initially was found to catalyse the oxidation of vanillyl alcohol (4-hydroxy-3-methoxybenzyl alcohol) to vanillin (4-hydroxy-3-methoxybenzaldehyde) [1]. The natural substrate of the enzyme could be 4-(methoxymethyl)phenol, as it is the only substrate known to induce VAO expression [2].

VAO is one of the designating members of the VAO/PCMH family of oxidoreductases that share a conserved FAD-binding domain [3–5]. Members of this family often bind their flavin cofactor in a covalent mode. The closest known and characterised relatives of VAO in the VAO/PCMH family are the bacterial enzymes eugenol oxidase (EUGO) and *p*-cresol methylhydroxylase (PCMH). While substrate specificities of these three enzymes differ, they all require their substrates to be *para*-substituted phenols [6–10]. The amino acid sequence of VAO is most similar to EUGO, sharing 47% sequence identity, while VAO and PCMH share 40% sequence identity. Interestingly, the sequence identity between the two bacterial enzymes is only 33%. All three enzymes bind their FAD cofactor covalently. In VAO, this bond is formed via an autocatalytic process, resulting in a covalent link between His422 and FAD [11]. In EUGO, the flavin is linked to His390 [12], while in PCMH, the flavin is covalently bound to Tyr384 [13].

**Fig 1:**
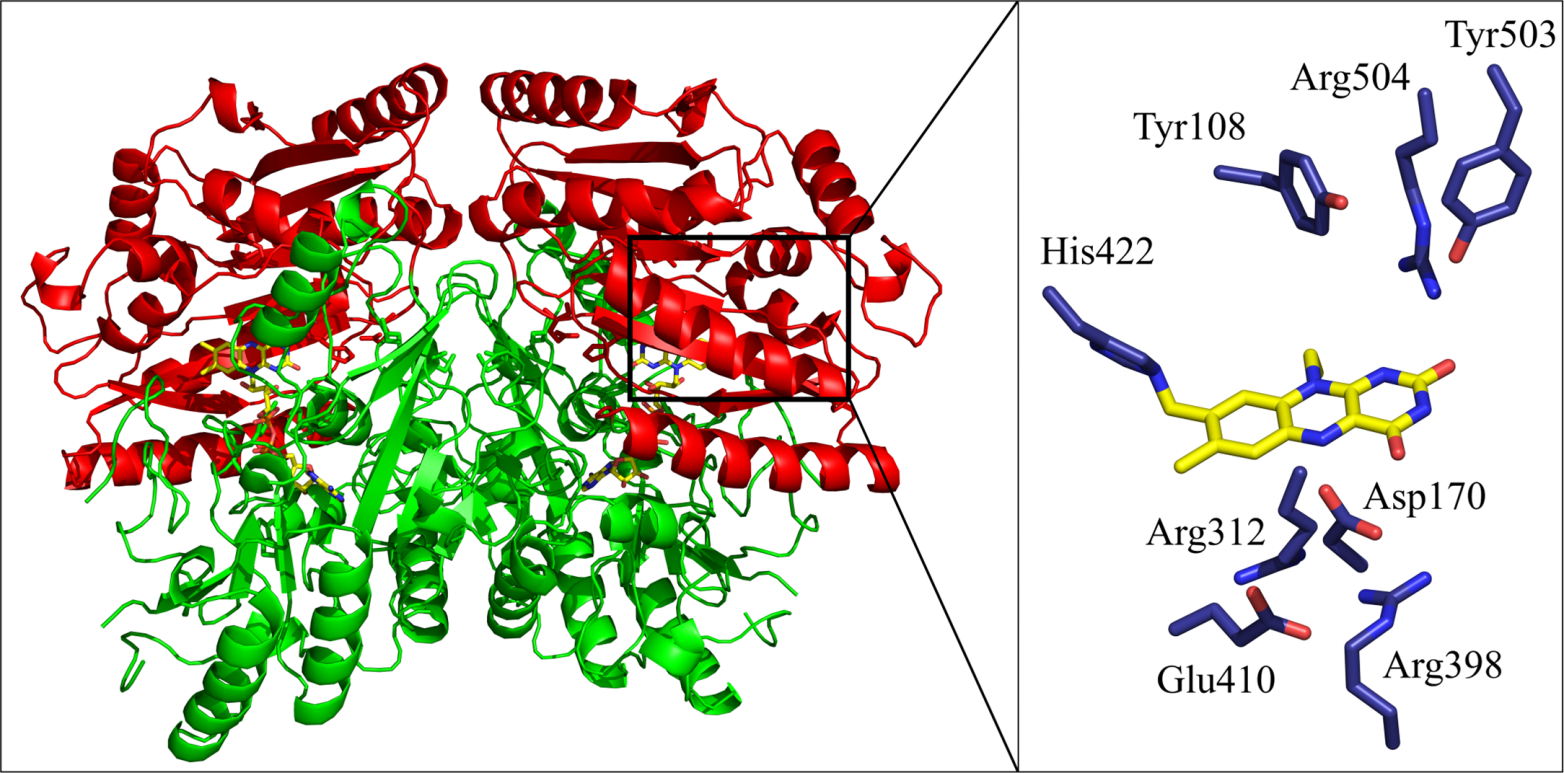
Three-dimensional structure of vanillyl alcohol oxidase (PDB ID: 1VAO). The dimer is shown as cartoon with the cap domain coloured in red and FAD-binding domain in green. A zoom into the active site at the interface of the two domains is shown with selected residues shown as blue sticks and the isoalloxazine ring of the covalently bound flavin cofactor in yellow.

VAO is a homo-octamer composed of a tetramer of dimers, where each subunit consists of 560 amino acids. Each subunit is organised into two domains, the FAD-binding domain and the cap domain [9], finding the active site at their interface (Fig 1). *In vitro*, VAO forms a mixture of dimers and octamers, with the octamers prevailing at physiological ionic strength [14,15]. EUGO and PCMH have different quaternary structures. EUGO is exclusively homodimeric, while PCMH consists of a heterotetramer. This heterotetramer consists of two FAD-binding subunits forming a homodimer and two cytochrome *c* subunits. The homodimeric structure of the FAD-binding subunits is the same in all three enzymes. See also supplementary S1 Fig for an overview of the quaternary structures of these three enzymes.

Important active site residues of VAO are Tyr108, Asp170, His422, Tyr503 and Arg504 (see the zoom into the active site in Fig 1). Tyr108, Tyr503 and Arg504 are proposed to be crucially involved in the deprotonation of substrates upon their arrival in the active site [9,16]. Asp170 has a multifunctional role. It is involved in the autocatalytic incorporation of the flavin cofactor and assists in increasing the redox potential of VAO (making it less negative) [17]. Asp170 can also act as active site base [17], and is involved in the enantioselective hydroxylation of 4-alkylphenols [18]. His422 increases the oxidation power of the FAD cofactor by increasing its redox potential (making it less negative) through covalent flavin binding [11,19].

**Fig 2:**
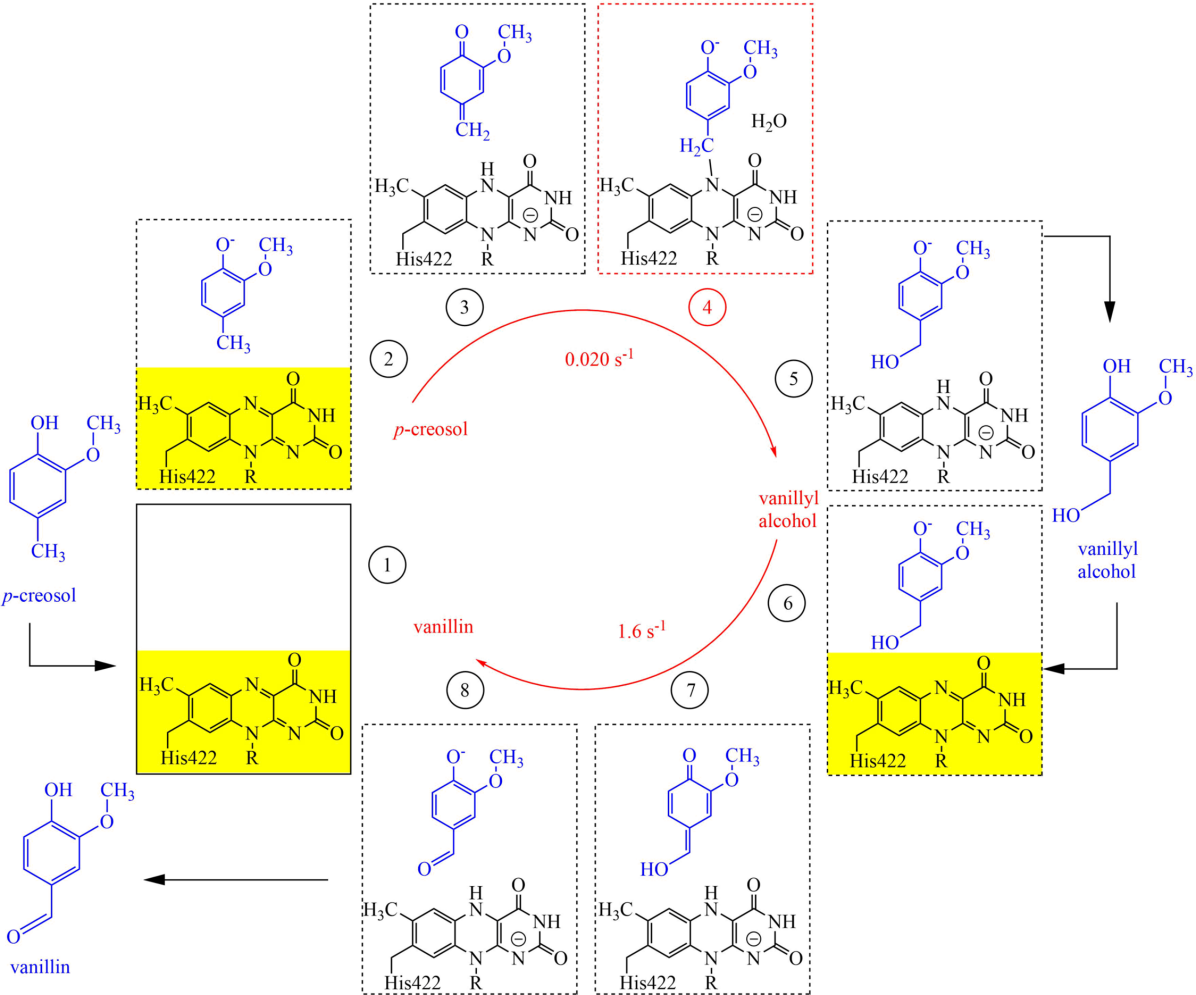
Overview of the reaction cycle from *p*-creosol to vanillyl alcohol and vanillin catalysed by VAO. Reaction rates at pH 7.5, 25°C are shown in the centre of the reaction cycle (data obtained from [22]). The rate-limiting step for the conversion of *p*-creosol to vanillyl alcohol is the decomposition of the air-stable flavin N5 substrate adduct (panel 4, marked in red). For the conversion of vanillyl alcohol to vanillin the rate-limiting step is the reduction of the flavin by the substrate (panel 7). In both reactions, the yellow flavin cofactor is rendered colorless through reduction by the substrate (occurring between panels 2 and 3 as well as 6 and 7). Molecular oxygen driven oxidation of reduced flavin (colourless) to oxidized flavin (yellow) occurs between panels 5 and 6 as well as panels 8 and 1, in the presence or absence of either vanillyl alcohol or vanillin.

The catalytic mechanism of VAO involves two half-reactions. In the reductive half-reaction, the enzyme-bound flavin is reduced by the phenolic substrate to generate a quinone methide intermediate [6,20]. In the oxidative half-reaction, the reduced flavin is oxidised by dioxygen, generating hydrogen peroxide. Depending on the substrate, there are differences in the reaction mechanism. With 4-(methoxymethyl)phenol, water addition to the quinone methide intermediate gives an unstable hemiacetal, which decomposes to 4-hydroxybenzaldehyde. VAO can produce vanillin in a one-step reaction from vanillyl alcohol as well as in a two-step reaction from *p-*creosol, where vanillyl alcohol is the decomposition product of the initially formed air-stable flavin-creosol adduct [21]. The reaction with *p*-creosol (Fig 2, plates 1 to 5) is rate-limited by the extremely slow decomposition of the flavin-N5 substrate adduct (Fig 2, plate 4) [9]. In the reaction of vanillyl alcohol to vanillin, there is no addition of water to the vanillyl alcohol quinone methide (Fig 2, plates 6 to 8) [20].

Although many details of the catalytic mechanism of VAO have been uncovered, it is unknown how the reaction participants enter and exit the active site. No path for solvent or ligand access to the active site is visible from the crystal structure of VAO. It is of fundamental interest to understand how reaction participants enter an enzyme’s active site to be able to identify catalytic bottlenecks. Such bottlenecks can then be used to guide substrate or enzyme redesign to eliminate them. This work provides a proof of concept for the use of an unbiased method to sample entry and exit pathways of a complex enzyme system.

We aim to discover the migration path(s) of reaction partners of VAO for the reaction from *p*-creosol to vanillin. Recent studies, with other enzymes, have pointed to the importance of remote residues for catalysis or the presence of multiple paths for ligands [23–27]. It is also worthy to note that during enzyme catalysis, the entry and exit of reaction partners may result in traffic jams in migration paths. We try to address these issues by modelling migration paths of reaction partners of VAO. Given the large size of VAO and the involvement of multiple ligands, it is conceivable that substrates and products use different paths.

In this study, we used Protein Energy Landscape Exploration (PELE), a Monte Carlo based program [28], to investigate the entry and exit paths of three phenolic ligands in VAO. Protonated and deprotonated phenolic ligands were used to be able to observe possible differences in binding energies during the simulations and thus establish when substrate deprotonation and product re-protonation happen in the reaction pathway of VAO.

An entry and exit path for phenolic ligands at the subunit interface, along with two additional exits paths, leading through the cap domain or the FAD-binding domain, were identified. It was found that protonated *p-*creosol freely migrated to the active site, while deprotonated *p-*creosol did not. In addition, the behaviour of dioxygen and hydrogen peroxide was modelled. Migration via the flavin *re* side turned out to be the shortest path for these molecules.

## Materials and methods

### System set-up

The initial coordinates for the VAO protein were taken from the 1VAO entry in the protein data bank (PDB) [9]. Although the protein is assembled as an octamer, due to computational limitations only the dimer, the smallest functional unit of VAO, was modelled. No allosteric regulation has been observed for VAO in its dimeric or octameric oligomerisation state. Loop deletion mutagenesis has revealed that the VAO dimer has similar catalytic properties as the octamer [29]. The protein structure was prepared assisted by the protein preparation wizard available in the Schrödinger software package [30]. All crystal water molecules were removed and missing hydrogen atoms were added to protein residues. Residues such as histidines, glutamates and aspartates were inspected for appropriate protonation states at pH 7.5 to match experimental conditions. PROPKA [31] and H++ web server (http://biophysics.cs.vt.edu/H++) [32] were employed to determine the pK_a_ of each residue. His56, His61, His313, His506 and His555 were modelled as ε protonated while all other His residues were δ protonated. Furthermore, the NE2 atom in the imidazole ring of His422 has no hydrogen atom since it is covalently bound to the C8M atom of the FAD cofactor and considered to be neutral. Missing residues from position 41 to 47, forming a loop at the surface of the protein, were added and minimised using PRIME [33].

The final system was composed of a total of 17674 atoms. Six phenolic and two small ligands were used including: COP, COD (*p*-creosol, where the P and D refer to the protonated and deprotonated state of the ligand; this notation is identical for all molecules), VAP, VAD (vanillyl alcohol) VNP, VND (vanillin) and dioxygen and hydrogen peroxide. All charges for the FAD cofactors were obtained through quantum mechanics/molecular mechanics (QM/MM) calculations inside the protein environment with an 8 Å layer of explicit waters. The QM region included all the isoalloxazine ring atoms and the QM/MM cut was made between the C1 and C2 atoms of the FAD molecule. All the ligands were optimized through QM calculations in an implicit solvent. QM/MM calculations used the all atom OPLS2005 force field [34], M06 functional [35] with the 6-31G** basis set [36] and Poisson Boltzmann Finite element (PBF) implicit solvent [37] (same functional and basis set used in QM). The parameter files for all ligands and the FAD cofactor are supplied as supplementary information S1.

### Ligand migration

PELE [28] was used to map the migration (exit and entry) path of different ligands between the VAO active site and the solvent. PELE is a Monte Carlo based algorithm that produces putative new configurations through a sequential ligand and protein perturbation scheme, side chain prediction and minimisation steps. A detailed description of the PELE methodology can be found elsewhere [28]. Ligand perturbation involves a random translation and rotation, while protein perturbation involves a displacement on the alpha carbon following one go the six lowest Anisotropic Normal Modes (ANM) [38]. These steps compose a move that is accepted (defining a new minimum) or rejected based on a Metropolis criterion for a given temperature. The combination of ligand and protein backbone perturbations results in an effective exploration of the protein energy landscape. This approach is capable of reproducing large conformational changes associated with ligand migration and has already been shown to produce reliable results [39–41].

For the entry simulations, the ligand was placed in six different positions at the bulk solvent. Similar parameters to the exit simulations were used, but with larger translations (from 1.25 and 2.5 Å) during substrate perturbations. The direction of such perturbations was kept for three consecutive steps. Moreover, for the entry simulations an adaptive scheme was used (using the new C++ version of PELE [42] plus an OBC solvent [43]). After a short simulation of 12 Monte Carlo steps, the adaptive scheme clustered the ligand position and new initial conditions were chosen, prioritizing those clusters with less population. Due to the use of different versions of PELE, the binding energies differ by a level of magnitude for ligands migrating in and out of VAO (see Results). In this context, it is important to note that the binding energies calculated by PELE are indicative of favourable or unfavourable positions for the ligand. While we refer to them as binding energies, they are interaction internal energies between the protein and the ligand, with a significant larger value than experimental ones, and should be analysed in a qualitative manner.

The exit simulation protocol was identical for all substrates and began by manually docking the phenolic molecule (COP, COD, VAP, VAD, VND,VNP) to the *si* side of the isoalloxazine ring of the flavin in the active site based on crystallographic evidence from the VAO structure with isoeugenol (PDB ID: 2VAO) [9]. Simulations were performed in equal number starting from both protein subunits. The phenolic ligand was then perturbed with random translations (from 0.75 and 1.75 Å) and rotations (0.05 and 0.25 rad) and requested to move away from the active site using PELE’s spawning procedure. This procedure selects a reaction coordinate (e.g. an atom-atom distance, ligand RMSD) and abandons the trajectories with worst values in the reaction coordinate to restart the simulation at the best value (which is constantly updated). Trajectories are abandoned only if they fall behind a user-defined range (from the best value). In our case, the spawning coordinate was the distance of the ligand center of mass to a point in the void volume of the active site (X, Y, Z coordinates 92, 25, 41 or 108, 47, 70 for simulations starting in the active site of chain A or B, respectively), with the distance range from the spawning coordinate being 4 Å. Protein perturbation was based on the six lowest normal modes [44] and side chain sampling including all residues within 8 Å from the ligand. Finally, after global minimisation had optimised the new configuration, this configuration was filtered with a Metropolis acceptance test. In this test, the energy is described with an all atom OPLS2005 force field [34] with a surface generalized Born solvent model [45]. Simulations were stopped when the wall clock limit time (48h) was reached or the phenolic ligand exited the protein completely. A total of 800 independent trajectories were produced for each ligand.

Simulations exploring exit paths from the active site of the small ligands dioxygen and hydrogen peroxide were performed using a non-biased approach as described previously [40], using random translations and rotations appropriate to achieve continuous paths (between 0.5 and 1.5 Å and 0.1 to 0.3 rad, respectively).

Data analysis was performed using VMD (Version 1.9.1, [46]), PyMOL (The PyMOL Molecular Graphics System, Version 1.7 Schrödinger, LLC.), and R (Version 3.3.1 [47]). Figures were created using the aforementioned programs, with the addition of GnuPlot (Version 4.4 [48]) and ChemDraw (Version 15.1.0.144, PerkinElmer Informatics).

Atom contacts between the ligands and the protein were calculated using a python script. A contact was defined as a distance smaller than 2.7 Å between an atom from any given residue and an atom belonging to the ligand. Root mean square fluctuation (RMSF) values of residues in the simulations were calculated using VMD (Version 1.9.1, [46]). Only RMSF values from residues in the chain of the dimer where the ligand was placed were used for the analysis.

### Protein structure comparison

To compare the structural conservation of path residues, the crystal structures of VAO, EUGO and PCMH (1VAO, 5FXE and 1DII respectively) were aligned using the PROMALS3D multiple sequence and structure alignment server [49]. The crystal structures were also compared using PyMOL (The PyMOL Molecular Graphics System, Version 1.7 Schrödinger, LLC.) and the built in alignment function.

### Protein sequence comparison

To compare the sequence conservation of path residues, a set of VAO-, EUGO- and PCMH-like sequences was identified. The protein sequences from crystal structures 1VAO, 1DII and 5FXE were used as queries for similarity searches with Consurf [50], searching the uniref90 database using default settings for the search of homologs. This approach resulted in three alignments (one each for VAO-, EUGO- and PCMH-like sequences). The obtained alignments were manually checked and VAO- and EUGO-like sequences that did not contain a histidine residue corresponding to His422 and His390 respectively were removed. PCMH-like sequences not containing a tyrosine residue corresponding to Tyr384 were removed as well. This selection was made because the named residues covalently bind the FAD cofactor, which has a significant impact on enzyme function [10–12].

The homology search resulted in 121, 117 and 30 VAO-, EUGO- and PCMH-like sequences remaining in the respective alignments (named MSA_VAO_, MSA_EUGO_ and MSA_PCMH,_ respectively). These alignments where then merged using the MAFFT webserver (mafft.cbrc.jp/alignment/software/merge.html) to match the residue positions (resulting in MSA_all_). The alignments were then uploaded to Consurf [49] to calculate the residue variety in percentages, always using the pdb structure 1VAO as query. This approach ensures that the same alignment positions are compared when the preservation of different residues in the three alignments is compared as described below; e.g. that residue number 422 in VAO is always compared to the residue present in the other sequences at the corresponding position in the three alignments. All alignments (MSA_VAO_, MSA_EUGO,_ MSA_PCMH_ and MSA_all_) can be found as supplementary information S1 Alignment.fa, S2 Alignment.fa, S3 Alignment.fa and S4 Alignment.fa, respectively.

An R script was used to analyse the preservation of different groups of residues in these alignments, amongst which residues involved in the paths identified in this study. We defined nine different groups of residues: the entire protein (560 amino acids), residues forming the cap domain (from residue 270 to 500), the *re* path residues (22 residues), the FAD path residues (36 residues), the cap path residues (25 residues), the subunit interface path residues (53 residues), a selection of random residues (58), surface residues of VAO (90 residues) and residues at the subunit interface (86 residues). Surface residues were defined using the built in tool of the SwissPDBviewer, with default settings (30% accessibility of residues by solvent in dimeric VAO) [51]. Interface residues, meaning residues at the subunit interface, were defined as residues within 4 Å of either subunit.

Preservation of these residues was analysed based on similar or dissimilar residues. Similar residues were defined according to the BLOSUM62 similarity parameters [52], resulting in six sets of residues (Trp, Tyr and Phe; Met, Ile, Leu and Val; His, Arg and Lys; Asn, Asp, Gln and Glu; Ser, Thr, Pro Ala and Gly; and Cys.)

The preservation of residues was analysed per position in the alignments and a cutoff of 50% preservation was used to categorise them as similar in the alignment. If preservation of the similar residues was above 90%, they were categorised as conserved in the alignment. This analysis was performed for MSA_VAO_, MSA_EUGO_ and MSA_PCMH_, for each of the nine groups of residues. This resulted in a percentage of similarity and a percentage of conservation for each group of residues for each sequence alignment of VAO-, EUGO- and PCMH-like sequences.

This analysis did not yet allow us to compare the conservation or similarity of these three alignments to each other. Different residues can be preserved in each of the three alignments, but we were interested in finding out how many residues are preserved in all three alignments (MSA_all_). We therefore performed the following analysis: we used the same definitions for similarity and conservation as above, but this time analysed the preservation of the nine groups of residues in MSA_all_. We added additional categories to this analysis: dissimilar and PCMH-unlike. Dissimilar positions were defined as positions less than 50% similar. PCMH-unlike positions are dissimilar positions for which VAO-like and EUGO-like sequences share a similar residue, but differ from PCMH. This resulted in percentages of similarity and conservation as well as percentages of dissimilarity and PCMH-unlikeness.

## Results

**Fig 3:**
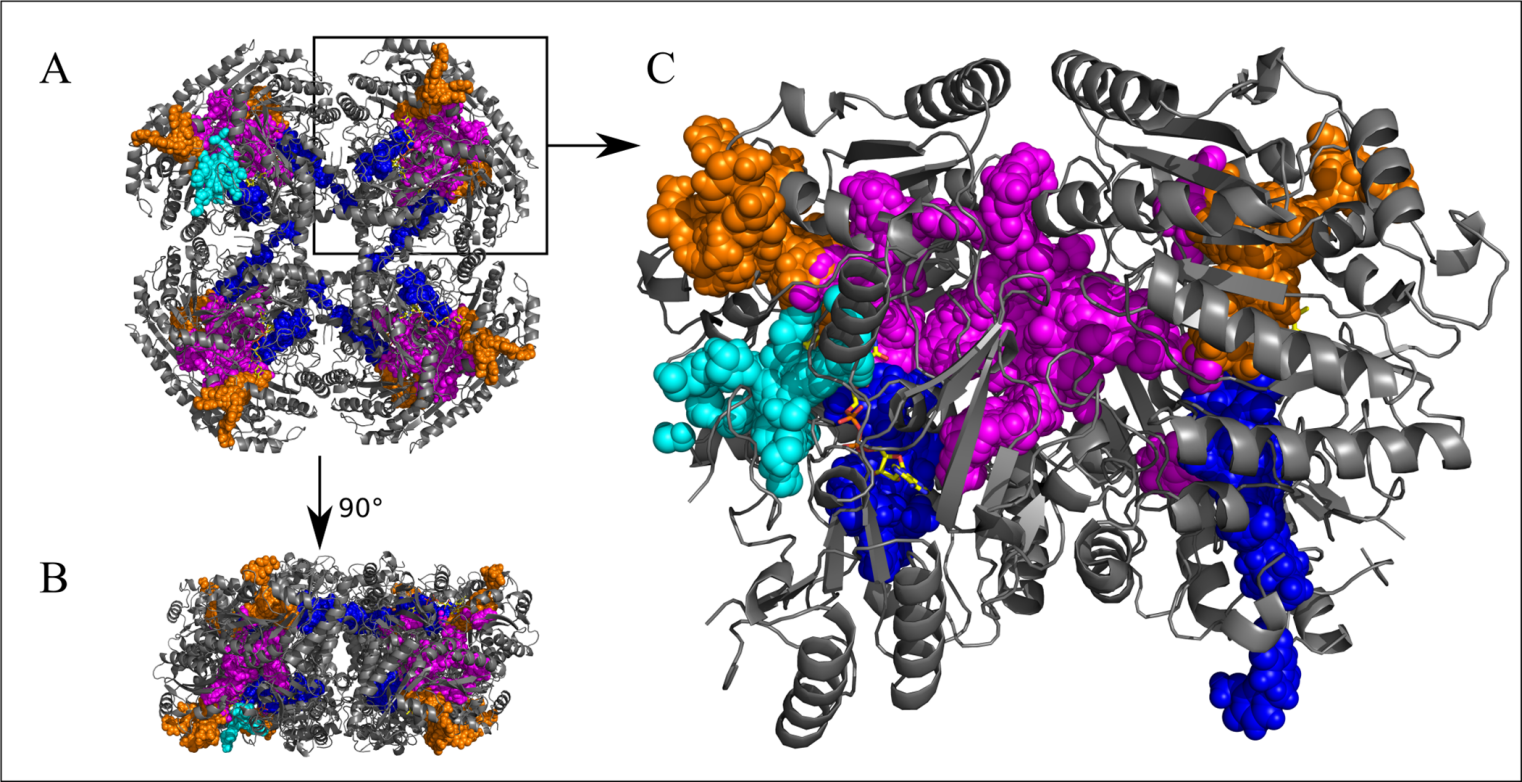
Three-dimensional structure of vanillyl alcohol oxidase and the four paths identified in this study. Vanillyl alcohol oxidase is shown as cartoon and coloured in grey, with FAD shown as sticks and coloured in yellow. (PDB ID: 1VAO). The paths identified in this work are shown through sphere representation of ligand positions in the trajectories. The cap path is coloured in orange, the FAD path in blue, the subunit interface path in magenta and the *re* path in cyan. A: Octameric VAO, seen from the front. B: Octameric VAO seen from the side. C: Dimeric VAO as seen in Fig 1.

Ligand migration simulations using PELE were run on the dimer of VAO with phenolic ligands COP, COD, VAP, VAD, VNP and VND, as well as with dioxygen and hydrogen peroxide. Fig 3 illustrates the location in VAO of all the paths identified through the approaches described in Materials and Methods.

Due to the stochastic nature of the PELE method, it is not possible to correlate the observed movements to biological timescales. Instead, the calculated binding energies (presented as interaction internal energies, see Methods), at different distances of the ligand from the FAD as well as the number of contacts between ligands and protein residues, were used to estimate their most likely paths as they move through the protein. Furthermore, we computed the flexibility of each protein residue during the simulations through RMSFs. This data can also be used to estimate if a path involves bottlenecks where the ligands tend to get stuck.

### Description of the entry path used by phenolic ligands

**Fig 4:**
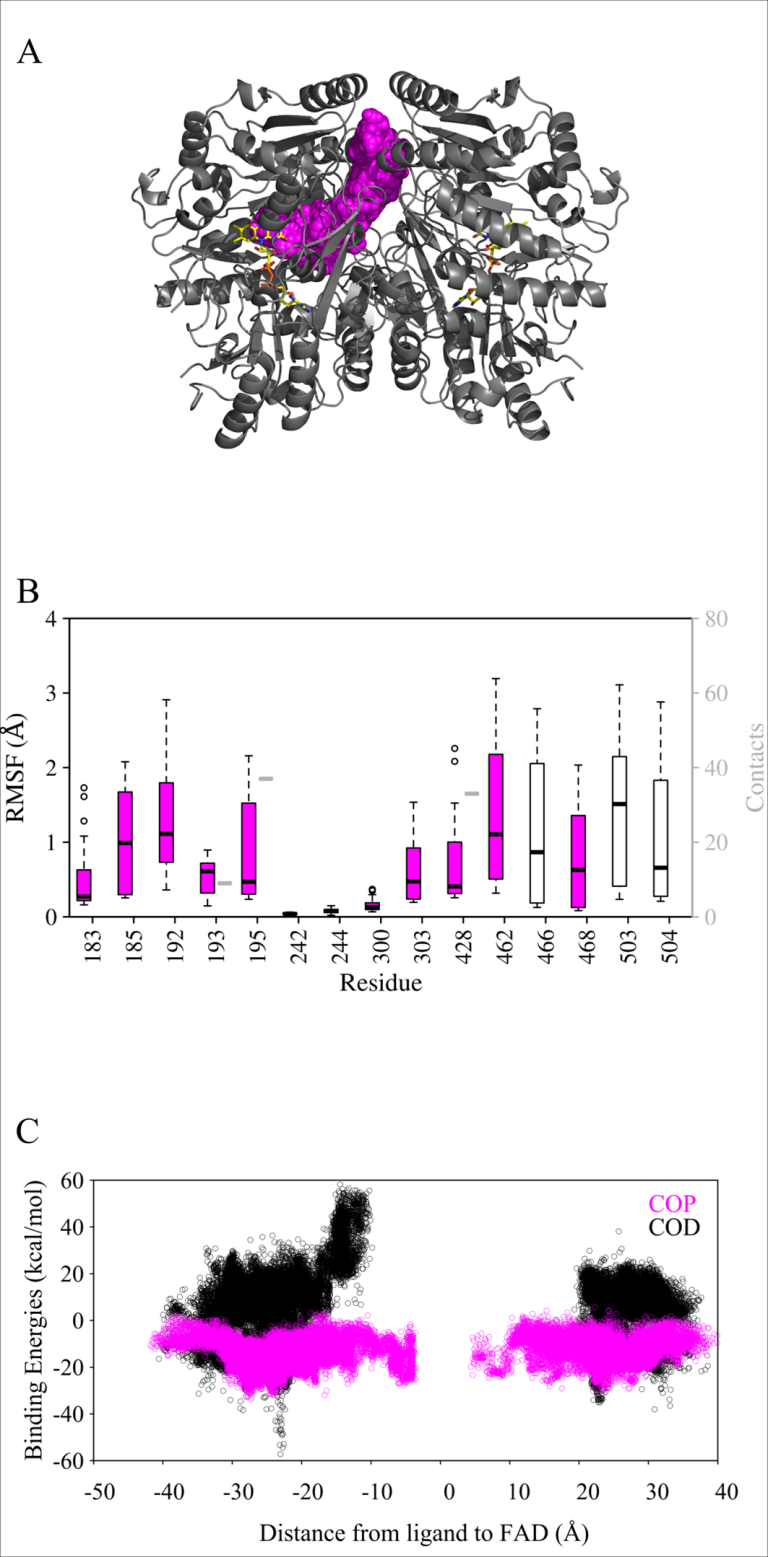
**Overview of the entrance path identified with COP (A) as well as RMSF and atom contacts (B, for COP) and binding energy data (C, for COP and COD).** A: The VAO dimer is shown as cartoon in grey and its FAD cofactor is shown as sticks in yellow. The entrance path is shown through sphere representation of ligand positions in the trajectories and coloured in magenta. For clarity, we only show data for an entrance simulation where COP migrates to the left-hand subunit. B: Highest RMSF values (black boxplot boxes) and atom contact counts with the ligand (grey boxplot boxes) of residues in VAO. Magenta filling of the boxplots indicates that the residue belongs to the entrance path. Residues that do not have atom contacts with the ligand but are highly flexible only have a black boxplot (for RMSF). Because data was obtained from only one simulation, the atom contacts do not have any variation. C: Scatterplot of binding energies as a function of the distance between the ligand and FAD. Negative x-values indicate the distance to the FAD in the other subunit. This graph shows that the path is symmetrical for COP (in magenta).

Using the adaptive PELE simulations, the surface of the VAO dimer was explored for COP entry, see Fig 4A. As stated, the ligand was placed in the bulk solvent and allowed to freely explore the surface and any possible entry path(s). It was found that using this approach, the ligand moved into the active site exclusively via the subunit interface. In order to refine the energy landscape along the entry path, we started additional simulations from the surface of the subunit interface with a reduced translation range (maximum ligand translation of 1.5 Å). These refinement simulations confirmed the entry for COP, but indicated that COD was unable to enter the protein via this path. Binding energies calculated showed that it was energetically favourable for COP to reach the active site via the subunit interface, but energetically unfavourable for COD to do the same (Fig 4C).

The COP entry path, which is identical to the third exit path described below, involves mainly residues Arg183, Val185, Asp192, His193, Met195, Met303, Ile428, Met462, His466 Ile468, Tyr503 and Arg504. Residues His466 and Tyr503 are easily changing conformation to grant the ligand access to the active site. This path is symmetrical, leading from the starting point at the subunit interface to either of the two active sites of the dimer, as can be seen in Fig 4C. The most contacted residues were His193, Met195 and Ile428 and the most flexible residues were Asp192, Gly462, His466 and Tyr503, as can be seen in Fig 4B.

### Overview of the three exit paths used by phenolic ligands (cap, FAD and subunit interface path)

To identify exit paths, simulations were performed for the dimeric protein and the ligands were placed into one of the two subunits and both subunits were used as starting points in equal amounts. We observed symmetry in our data (see Fig 3): some paths were fully symmetrical in simulations starting from either subunit and other paths were partly symmetrical, most likely due to incomplete sampling as can be seen from Figs 3 and 4A. The fact that we observed identical results independent of the starting point is a good indication that all potential paths have been sampled. Extrapolating data from each of the two subunits to the other subunit gave fully symmetrical paths, which were continuously connected.

**Fig 5:**
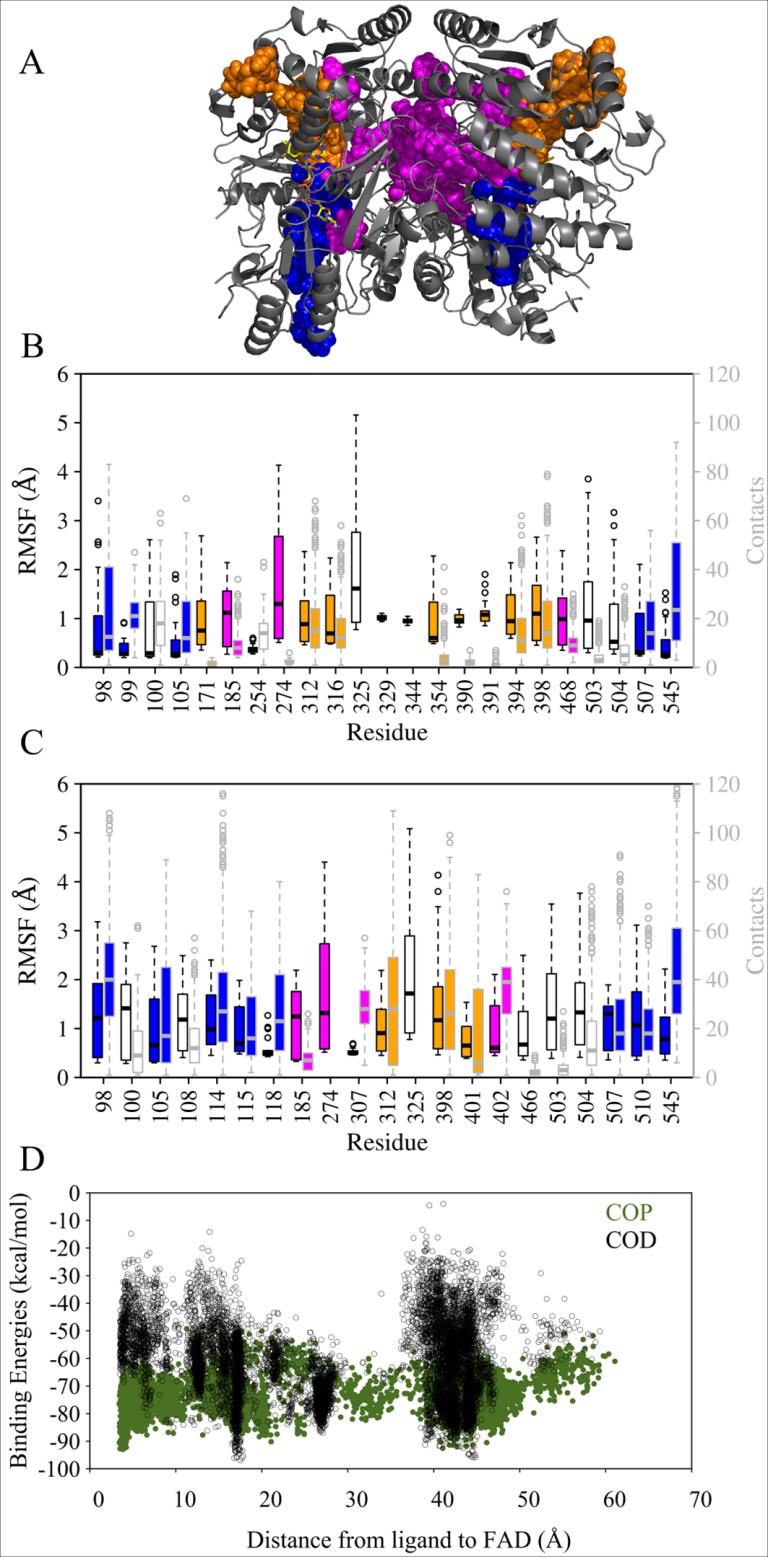
**Overview of the exit paths identified with COP and COD (A), RMSF and atom contacts (B for COP and C for COD) and binding energies (D).** A: The VAO dimer is shown as cartoon in grey and its FAD cofactor is shown as sticks in yellow. The paths identified in this work are shown through sphere representation of ligand positions in the trajectories. The cap path is coloured in orange, the FAD path in blue and the subunit interface path in magenta. B: Plot of simulations with COP, highest RMSF values (black boxplot boxes) and atom contact counts with the ligand (grey boxplot boxes) of residues in VAO. Coloured filling of the boxplots indicates that the residue belongs to the path of that colour. C: Plot of simulations with COD, highest RMSF values (black boxplot boxes) and atom contact counts with the ligand (grey boxplot boxes) of residues in VAO. Coloured filling of the boxplots indicates that the residue belongs to the path of that colour. D: Scatterplot of binding energies as a function of the distance between the COP and COD (in green and black) to FAD.

Three independent exit paths were observed for simulations with phenolic ligands, as shown in Fig 3 and parts A of Figs 4, 5, S2 and S3. The paths shown in different colours correspond to i) the cap path (in orange) where ligands exit the protein through the cap-domain, ii) the FAD path (in blue) where ligands traverse the protein through the FAD-binding domain, and iii) the subunit interface path (in magenta), which corresponds to a path connecting the subunits (and agreeing with the entry path described above).

Not all simulations led to the ligand leaving VAO. Some simulations showed that the ligand also moved from one path to the other, resulting in mixed paths. Only one of the three paths, the cap path, is exclusively connecting the active site to the solvent. The other two paths identified, the FAD path and subunit interface path, lead to interfaces between subunits or dimers of VAO. Paths leading to the interface of subunits or dimers allow the enzyme on one hand to connect the different active sites of the subunits (Fig 3 and parts A of Figs 4, 5, S2 and S3). On the other hand, these paths also allowed the ligands to exit via the interface of the dimer or the subunit, as there is sufficient space for the ligands available there. Because these paths can be expected to work into both directions, in and out, they could also act as another access point for the ligands to the active site.

However, analysis of residue flexibility represented by RMSF data as well as atom contact counts has to be taken into account to discover possible bottlenecks in these paths. The RMSF values and atom contacts calculated per simulation were combined and plotted in boxplots (parts B and C of Figs 4, 5, S2 and S3). The more flexible residues, mostly located at the surface, were often also the most contacted ones. Those most flexible surface residues were Arg274 and Arg325, while the buried ones showing larger flexibility were Arg398, Tyr503 and Arg504. Residue Lys545 was the most contacted residue for all ligands except VNP. Other flexible and/or highly contacted residues in simulations with most ligands were Trp98 (not with VNP), Ile100 and Asn105 (not with VAD and VNP) Val185 (not with VAP), Arg312 (not with VAD and VNP), surface residue Ser329 (not with COD) and Leu507 (not with VNP).

The binding energies of all the phenolic ligands were on comparable scales, but the range of the calculated binding energies varied between ligands, as can be seen in supplementary S2C Fig. The binding energies plotted against the distance of the ligand from the FAD spread least towards higher energy values for COP and VAP compared to COD and VAD. When comparing the data from the entry path and the exit paths, COP showed very little spreading of the data and presents lower binding energies than COD in both cases. Combining this information suggests that it is energetically more favourable for protonated substrates to migrate into and out of VAO than for deprotonated substrates or products.

In the following sections, each path and the behaviour of path residues will be described individually.

### Detailed description of the cap path

In this path, ligands migrated to the “upper-part” of the active site (orange in Figs 3, 5, S2 and S3), passing Glu410, Trp413, Arg312 and Arg398 to exit the protein in the cap domain. Arg398 and Trp413 have been proposed to be involved in a size-exclusion mechanism that could limit the size of the substrate-binding pocket [9]. The cap path requires the ligands to pass Arg312 and Arg398, which are among the most frequently contacted and most flexible residues for most ligands. Arg398 interacts with Glu410 and Trp413 and it is this interaction that the ligands need to disrupt to exit the active site via this route. Simulations often showed ligands to be trapped between the two arginines as they tried to pass this portal. This path was the shortest of the three identified for the phenolic ligands, but contained a bottleneck composed of residues Arg312, Arg398, Glu410 and Trp413 (as can be seen in Figs 3, 4, S2 and S3). Combining the data for all the ligands, residues belonging to the cap path that are among the most flexible or contacted residues were Leu171, Arg312, Leu316, Tyr354, Pro390, Glu391, Asn392, Val394, Arg398 and Thr401.

### Detailed description of the FAD path

In this path (blue in Figs 3, 5, S2 and S3), ligands migrated through Tyr503 and Arg504 to move closely along the FAD phosphate-ribityl chain towards the adenosine monophosphate part of the cofactor and to exit the protein between Trp98, Leu507 and Lys545. Trp98 is required to move aside, which is reflected in the RMSF and contact data (as can be seen in Fig 3B and 3C in the middle panel and supplementary S2A and S2B Fig). This path led to the dimer-dimer interface in octameric VAO, where we find sufficient space for the ligand to exit VAO or migrate to the neighbouring dimer in the octameric symmetry unit. However, Trp98, Leu507 and Lys545 constituted a bottleneck in this path. Combining the data for all the ligands, residues belonging to the FAD path that were among the most flexible or contacted residues were Trp98, Pro99, Asn105, Arg114, Val115, Ser118, Phe424, Thr505, Leu507, Met510, Lys545 and Ser546.

### Detailed description of the subunit interface path

In this path, ligands moved towards the subunit interface of the VAO dimer (magenta in Figs 3, 5, S2 and S3). The behaviour of different ligands modelled varied slightly in this path and they followed different minor variations of it, however all these minor paths led to the subunit interface. Simulations that were started with a ligand in one subunit sometimes showed the ligand completely entering the active site of the other subunit. Ligands were frequently found in the upper part of the subunit interface, where they were already surrounded with solvent. As stated, simulations mapping the entry path also showed the ligand passing through the same area.

Three residues clearly involved in the subunit interface path, but not showing up as frequently contacted or highly flexible residues in the analysis, were Asp192, Met195 and Glu464. Ligands passed quickly through a portal formed by Glu464, Asp192 and Met195, and from there directly into the subunit interface (for an illustration see supplementary S4 Fig). The portal formed by these three residues appeared to be the easiest for the ligands to pass through, not requiring significant movements of large residues and not involving a high frequency of contacts. The portal in this path connects the two active sites in the VAO dimer to each other.

**Fig 6:**
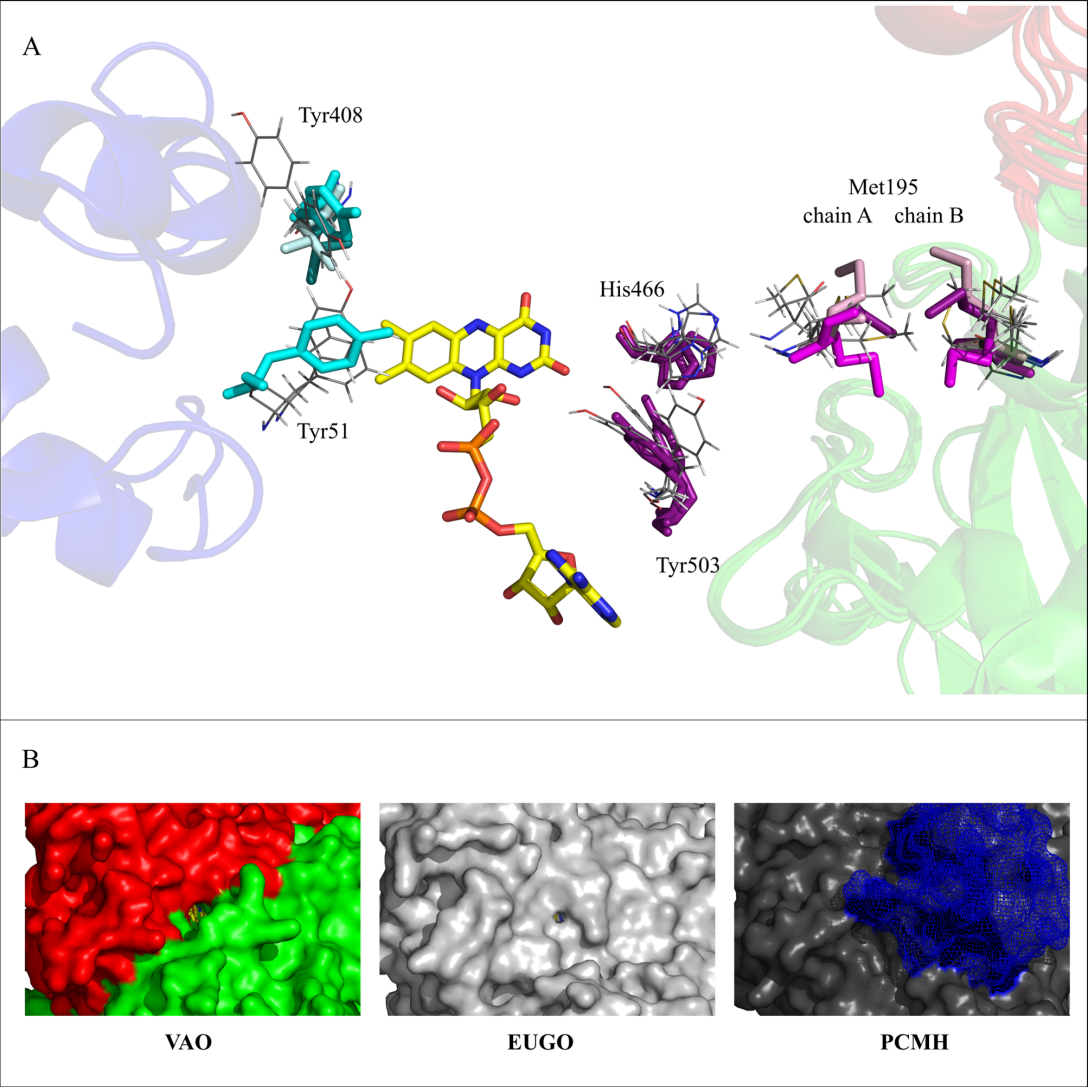
**Details of the ligand migration paths identified in this study (A) and illustration of the surface at the *re* path in VAO, EUGO and PCMH (B).** A: Crystal structures of VAO, EUGO and PCMH as well as selected frames from our simulations were aligned using PyMOL. Selected crystal structure residues (labelled with VAO numbering) are shown as sticks. Residues involved in the subunit interface path and their conformations in VAO are coloured in magenta. The conformations of the corresponding residues in EUGO and PCMH, are coloured in lighter or darker shades of magenta, respectively. Residues involved in the *re* path and their conformations in VAO are coloured in cyan. The conformations of the corresponding residues in EUGO and PCMH, are coloured in lighter or darker shade of cyan, respectively. Note that the loop carrying Tyr51 is completely absent in EUGO and PCMH and that Tyr408 present in VAO is a leucine in EUGO and PCMH. Selected residues from selected frames of our simulations are shown as lines, in grey. Frames showing the movement of Tyr51 and Tyr408 were taken from simulations with dioxygen, those showing movement of His466, Tyr503 and Met195 from simulations with phenolic ligands that were migrating through the subunit interface path. The respective other monomer of the three crystal structures is visible on the right of the figure as cartoon in red (cap domain) and green (FAD-binding domain). On the left, the cytochrome *c* subunit from PCMH is visible as cartoon, coloured in blue. B: Surface representation of the surface of the *re* path to illustrate the size of the channel leading to the *re* side of FAD in VAO, EUGO and PCMH. The proteins are shown as surfaces and the FAD cofactors as sticks, coloured in yellow. Note the different size of the channel in VAO and EUGO, as well as the blockage by the cytochrome *c* subunit in PCMH (blue mesh).

Additional residues involved in this path are His466 and Tyr503, which were moving aside to grant access to the active site in the entry simulations and were also allowing ligands to exit. See Fig 6A for details on the movement of His466 and Ty503 as well as Met195 in our simulations. Residues that were in contact with ligands and that line the subunit interface were Arg463, Arg300 and Met303. Combining the data for all the ligands, residues belonging to the subunit interface path that were among the most flexible or contacted residues were Arg183, Val185, Tyr187, Trp194, His197, Tyr244, Tyr249, Ser255, Arg274, Arg300, Met303, Asn307, Thr310, Ala356, Met402, Met462, Ile468 (see also parts B and C of Figs 5, S2 and S3).

### Detailed description of the paths used by dioxygen and hydrogen peroxide

The co-substrate dioxygen and co-product hydrogen peroxide were placed on the *si* or *re* side of the isoalloxazine ring of the FAD cofactor and left to explore the VAO dimer. On the *re* side of FAD, a possible oxygen binding pocket is located as indicated by a bound chloride ion in PDB ID: 1VAO and by a water molecule in the same position in PDB ID: 2VAO [9,53].

**Fig 7:**
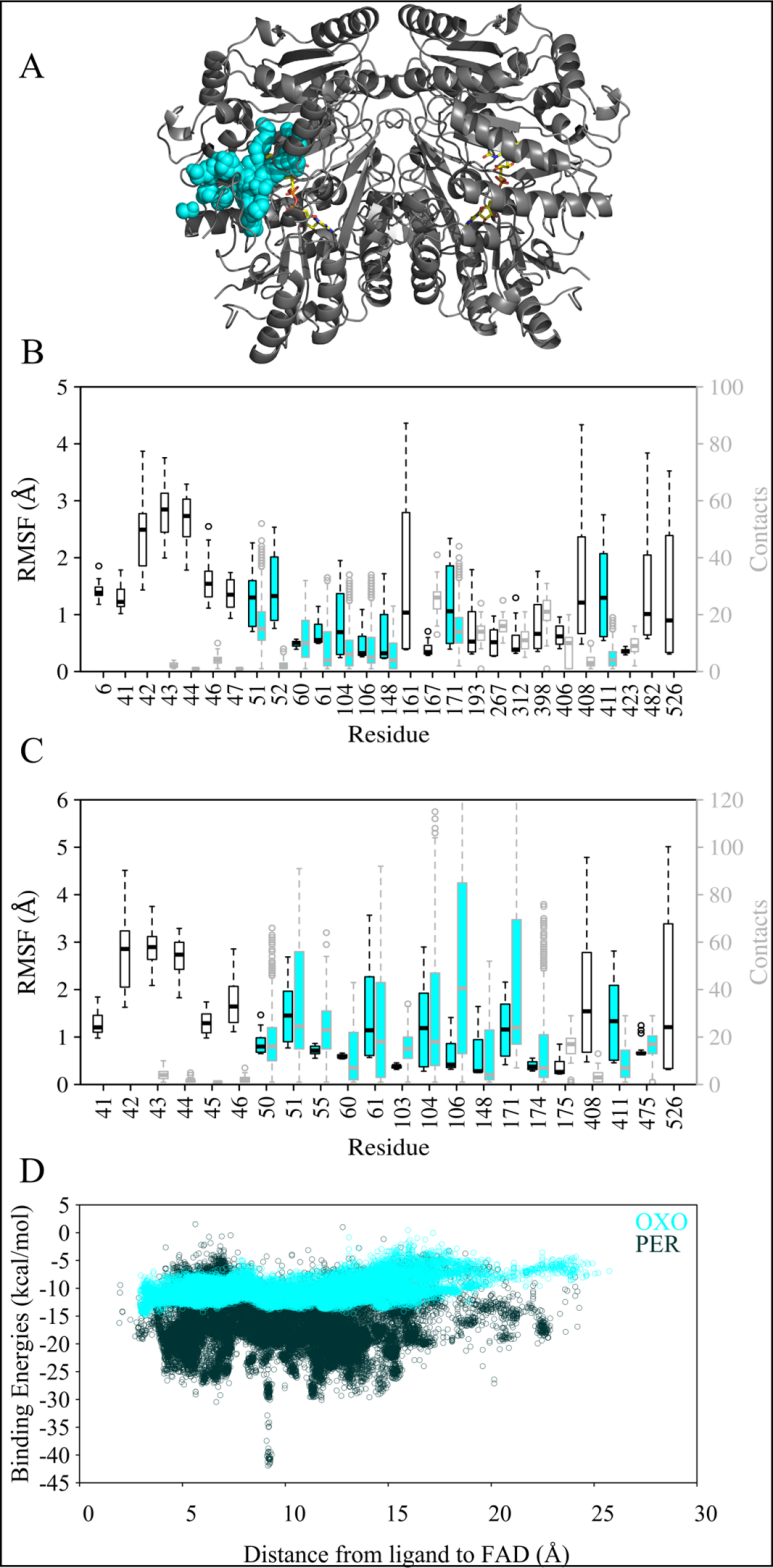
**Overview of the *re* path identified with dioxygen (OXO) and hydrogen peroxide (PER) (A), RMSF and atom contacts (B for dioxygen and C for hydrogen peroxide) and binding energies (D).** A: The VAO dimer is shown as cartoon in grey and its FAD cofactor is shown as sticks in yellow. The paths identified in this work are shown through sphere representation of ligand positions in the trajectories. The re path is coloured in cyan. B: Plot of simulations with dioxygen, highest RMSF values (black boxplot boxes) and atom contact counts with the ligand (grey boxplot boxes) of residues in VAO. Cyan filling of the boxplots indicates that the residue belongs to the *re* path. C: Plot of simulations with hydrogen peroxide, highest RMSF values (black boxplot boxes) and atom contact counts with the ligand (grey boxplot boxes) of residues in VAO. Coloured filling of the boxplots indicates that the residue belongs to the path of that colour. D: Scatterplot of binding energies as a function of the distance between dioxygen (cyan) or hydrogen peroxide (black) to FAD.

When placed on the *si* side of FAD, dioxygen and hydrogen peroxide behaved similarly to the phenolic ligands, but did not leave the enzyme after 200–250 simulation steps, probably due to under sampling. There is a difference in the behaviour of the co-ligands though: they both rarely used the FAD path and hydrogen peroxide had a clear preference for the cap path, almost exiting the protein when migrating through that path. When placing the co-ligands on the *re* side of FAD, they were able to leave the enzyme after 50 to 250 simulation steps via a path through the interface of the cap and FAD domain. This path will be referred to as *re* path for the remainder of this text and is illustrated in cyan in Figs 3 and 7. Contrary to the paths identified for phenolic ligands, this path is visible as an access channel to the active site from the crystal structure of VAO (see Fig 6B).

Binding energies for dioxygen and hydrogen peroxide were found to be on a different scale from those of the phenolic ligands, which is to be expected due to the difference in size of these molecules (computed binding energies are extensive properties), for comparison see parts D of Figs 4, 5 and 7. However, binding energies were comparable for these co-ligands when placed at the *si* or *re* side of FAD. In both cases, variations in binding energies for hydrogen peroxide were larger than for dioxygen, but overall binding energies were more negative for hydrogen peroxide (Fig 7D). This might suggest that hydrogen peroxide more likely gets stuck in local energy minima, which could mean that dioxygen reaches the active site in a faster and more directed manner than hydrogen peroxide leaves it.

Surface residues found in the analysis of RMSF and atom contacts were residues Lys43, Asp44, Ile46 and Arg482. Residues of the *re* path among the most flexible or contacted residues were Tyr51, Met52, Thr55, Pro60, His61, Gly103, Arg104, Ser106, Tyr148, Leu171 Gly174, Leu411 and Lys475 (as illustrated by Fig 7 parts B and C). Tyr51 and Tyr408 needed to move aside to allow dioxygen and hydrogen peroxide to leave the active site, a behaviour likely determining access to the flavin *re* side for these molecules. Tyr51 is also positioned close enough to the proposed oxygen binding pocket that it could be involved in FAD re-oxidation. See also Fig 6A for details of the movement of Tyr51 and Tyr408 in our simulations.

A movie illustrating all the paths is available in the supplementary data (S1 Movie).

### Structural conservation of the identified migration paths in VAO, EUGO and PCMH

**Fig 8:**
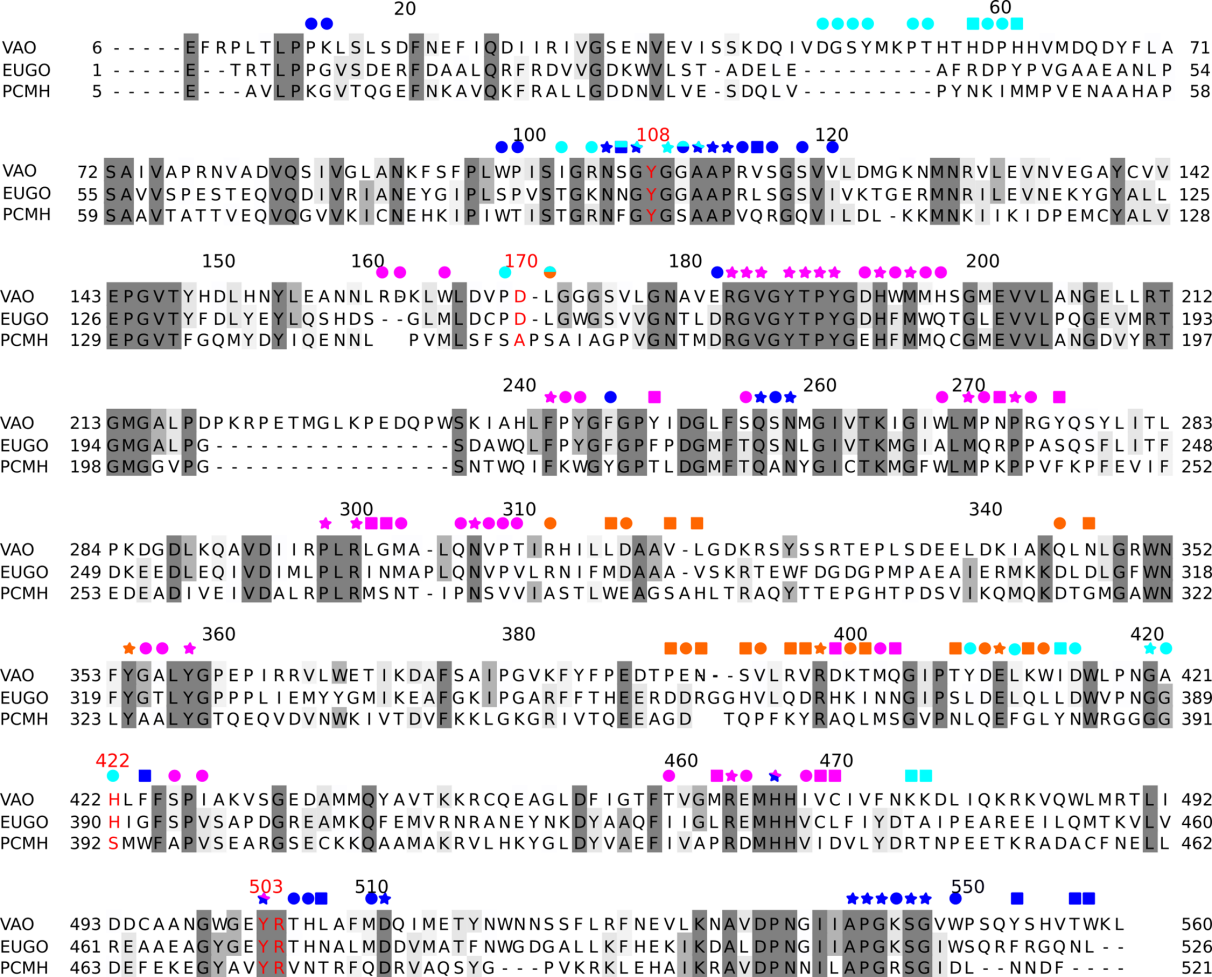
Structure-based sequence alignment of VAO, EUGO and PCMH. Numbering of residues at the beginning and end of each line correspond to the numbering used in the crystal structures. Numbers on top of the alignment correspond to the numbering of VAO sequence P56216.1 and is the same as used throughout this study. Coloured symbols indicate the path the residue underneath it is involved in. Orange, blue, magenta and cyan was used for the cap, the FAD, the subunit interface and the *re* path respectively. Red indicates functionally important residues. Bi-coloured symbols indicate the residue is involved in two paths. The shape of the symbol indicates preservation of the residue, stars (present in all three structures), circles (present in two of the three structures) and squares (present in only one of the three structures).

We analysed the structural conservation of path residues in three crystal structures of VAO, EUGO and PCMH. Fig 8 shows the obtained structure-based sequence alignment and illustrates the conservation of residues in the different paths through coloured symbols. We also compared the conformations of the most conserved residues involved in the paths in the three aligned structures (indicated by star symbols in Fig 8). With the exception of Met195, Gly420, His466 and Tyr503 the conformations of all conserved residues in the crystal structures were identical.

The different conformations of these four residues will be described below. Met195 was found to have different conformations in the crystal structures of VAO, EUGO and PCMH. These conformations correspond well to the movement by Met195 observed in our simulations (see Fig 6A for an illustration). Gly420 is located in a surface loop, which is in close proximity to the cytochrome *c* subunit in PCMH. This loop adopts a different conformation in PCMH than in VAO and EUGO. This could be in part due to the presence of the cytochrome *c* subunit in PCMH, which is absent in EUGO and VAO, and in part due to the covalent histidyl linkage to the FAD cofactor in VAO and EUGO but not PCMH. His466 and Tyr503 have already been observed to have two conformations in the high resolution crystal structure of PCMH (PDB ID: 1DII [8]). The conformational freedom of these residues is not observed in the other crystal structures but is in agreement with the movements of His466 and Tyr503 observed in our simulations (see Fig 6A for an illustration). It is noteworthy that the *re* path identified in VAO leads to where the cytochrome *c* subunit is located in PCMH. We compared this channel in VAO, EUGO and PCMH and found that it is non-existent in PCMH and narrower in EUGO compared to VAO (see Fig 6B for an illustration).

Several residues involved in the paths we identified in VAO are structurally conserved in EUGO and PCMH. If the paths we identified are also present in so far uncharacterised VAO-, EUGO- and PCMH-like enzymes, conservation of path residues should also be observed. This would further support the existence of the paths we have identified. We therefore performed an analysis of sequence conservation.

### Sequence conservation of the identified migration paths in VAO, EUGO and PCMH

We analysed sequence conservation in MSA_VAO_, MSA_EUGO_ and MSA_PCMH_ to establish if the paths identified in this study are also conserved in so far uncharacterised VAO-, EUGO- and PCMH-like enzymes. To this purpose, we used MSA_VAO_, MSA_EUGO_ and MSA_PCMH_, as described in Materials and Methods. We also defined nine groups of residues to be analysed in detail: the residues in the four paths (cap, FAD, subunit interface and *re* path), surface or interface residues, a random selection of residues, residues in the cap domain and the entire protein.

**Fig 9:**
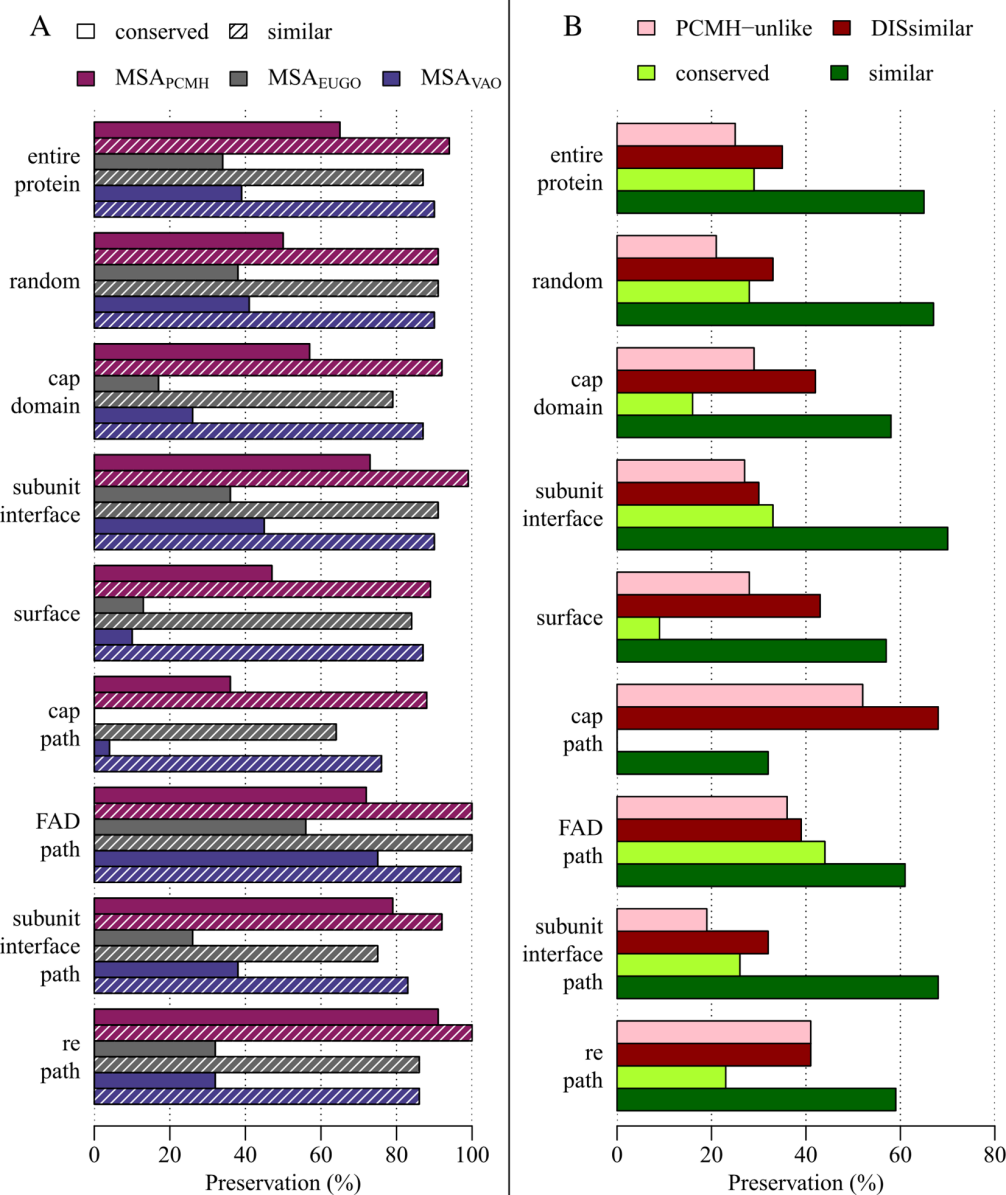
Sequence preservation of ligand migration paths. Preservation of residues in the entire protein, a random selection (random), the cap domain, the subunit interface, on the surface of the protein (surface), in the cap path, the FAD path, the subunit interface path and the *re* path. A: Sequence preservation of ligand migration paths within MSA_VAO_, MSA_EUGO_ and MSA_PCMH_. B: Sequence preservation of ligand migration paths within MSA_all_.

We found that within these three alignments, all sequences were more similar than dissimilar (see Fig 9A for all data). Residues in the cap path and at the surface were least similar and conserved in all three alignments. Residues in the FAD path and at the subunit interface were most similar and conserved in all three alignments. Note that if similarity or conservation is higher than in the entire protein or the random selection, this indicates higher selective pressure on this group of residues, and lower selective pressure if the opposite is the case.

Preservation of the groups of residues analysed in this manner should not be used as an indication of similarity or conservation within all three sets of sequences (Fig 9A). At one position in MSA_VAO_, a residue can be conserved or similar, but a different residue can be conserved or similar in the MSA_EUGO_ or MSA_PCMH_. To be able to determine overall preservation of residues in all three sets of sequences (MSA_all_), we continued our analysis as described in Materials and Methods.

The results of this analysis are summarised in Fig 9B. We found that when analysing the full length MSA_all_, 29% of all positions were more than 90% conserved, and 25% of all positions were similar in VAO and EUGO, but varied in PCMH (were PCMH-unlike). Residues in the cap path were most dissimilar, and also the most PCMH-unlike. Residues in the subunit interface path and at the subunit interface were most similar and residues in the FAD path and at the subunit interface were most conserved. It is noteworthy that all dissimilar residues in the *re* path are only dissimilar due to PCMH-like sequences.

It is conspicuous that the percentages of similarity and conservation in the two analyses differ significantly due to the preservation of different residues in VAO-, EUGO- and PCMH-like sequences. This is reflected in the percentages of dissimilarity and PCMH-unlikeness. Note the difference in preservation between residues in the cap domain and the cap path, which indicates that there is less selective pressure (or selective pressure for different residues) on residues in the cap path and the entire cap domain.

## Discussion

In this study, we have identified, for the first time, ligand and co-ligand migration paths in a member of the VAO/PCMH family. The process of ligand migration to the active site is often neglected when discussing enzyme mechanisms. The advances of computational methods have made it feasible to study this process in more detail, allowing better fundamental understanding of how enzymes work. Here, we will first address the implications our findings have for our understanding of the mechanism of VAO, and then expand further to cover the close relatives PCMH and EUGO, to finally discuss what is known about (co-)ligand migration paths in the entire VAO/PCMH family.

The residues discussed below are possible targets for site-directed mutagenesis to attempt to block or open a path. The effect of such modifications on enzyme activity can then be determined experimentally. Flavoenzymes are well suited to study enzyme kinetics using stopped flow instruments due to their reaction mechanisms involving a reductive and oxidative half-reaction.

### Ligand migration paths in VAO

**Fig 10:**
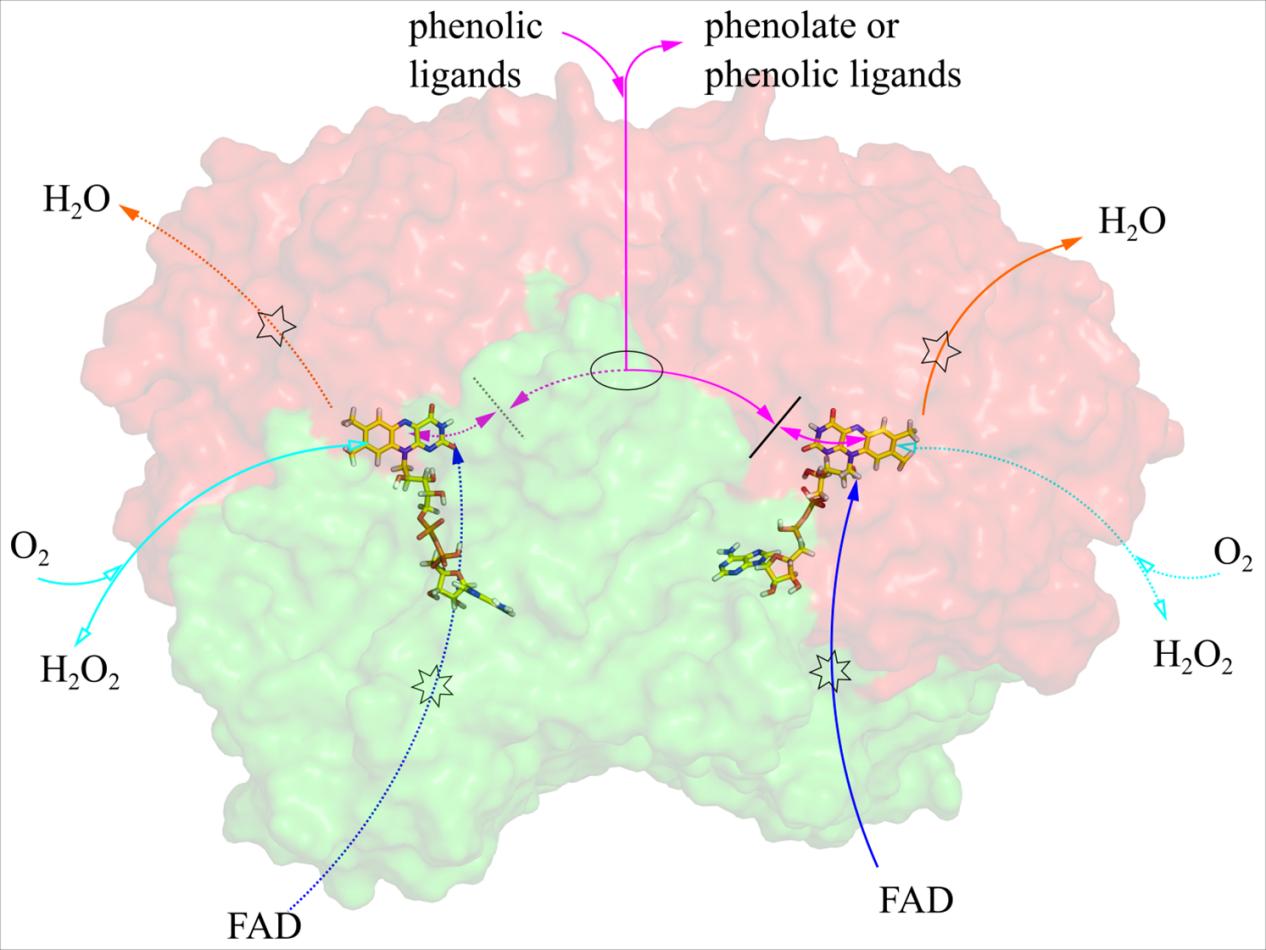
Overview of the identified paths and the ligands proposed to migrate through them (uni-or bi-directional). The VAO dimer is shown in surface representation, with the cap domain coloured in red and the FAD-binding domain coloured in green and the FAD cofactor shown as sticks in yellow. Coloured arrows indicate the paths for phenolic ligands (cap path in orange, FAD path in blue and subunit interface path in magenta). Filled in arrowheads indicate paths to the *si* side of FAD and empty arrowheads paths to the *re* side of FAD. Drawn out lines indicate the path is on the side of VAO facing the viewer, and dotted lines indicate the path is on the back side of VAO. Note that the two subunits are rotated 180 degrees relative to each other in the VAO dimer and that a path pointing towards the viewer in the right monomer is pointing away from the viewer in the left monomer and vice versa. The location of the concierge residues, His466 and Tyr503, is represented by a black line. The portal formed by Met192, Met195 and Glu464 is indicated by an ellipse. The bottlenecks formed by Arg312 and Arg398 in the cap path and Trp89 and Lys5454 in the FAD path are represented by pentagrams and heptagrams, respectively.

We have identified three migration paths in VAO for phenolic ligands (see Fig 10 for an overview). We do not observe any preference for a specific path for any of the three phenolic ligands analysed (*p*-creosol, vanillyl alcohol and vanillin). Two of these migration paths, the cap and FAD paths, are less likely paths for these ligands. They require large movements of amino acid side chains (namely Arg312 and Arg398 or Trp89 and Lys545, respectively) to allow the phenolic ligand to pass, which make them more difficult passages to the active site. This can be seen in the simulations, where the ligands often got stuck next to these residues. It is also evident from the high number of atom contacts ligands experienced with these residues. VAO also accepts phenolic substrates with larger hydrophobic side chains than the ligands studied here [9]. These larger ligands would be prone to have even more difficulties passing the bottlenecks in the cap and FAD path. Therefore, and because it represents the only entry and exit path identified, we argue that the VAO subunit interface path is the most probable route for phenolic ligands to enter or leave the active site.

In the subunit interface path, two residues are observed to change conformation to allow ligands to leave the active site. These two residues, His466 and Tyr503, do not present obstacles for the migration of the ligands but are in a closed conformation once a ligand is bound. Our results suggest a dual role for Tyr503, which is known to be involved in substrate deprotonation [16], and which we can show here to be also involved in substrate migration. His466 and Tyr503 can be regarded as concierges, limiting access to the active site. Interestingly, in the crystal structure of PCMH (PDB ID: 1DII [8]), two possible conformations are observed for His436 and Tyr473, which correspond to His466 and Tyr503 in VAO (see Fig 6A for an illustration). We would also like to highlight Met195 as the most flexible of the three portal residues to complete the list of flexible and probably functionally important residues in this path. It is of interest to keep in mind that this path shows that the two active sites of the VAO dimer are connected via this portal.

We were also able to demonstrate that protonated phenolic substrates enter the enzyme but deprotonated ones do not. Energy profiles of exit simulations also indicate more favourable binding of the protonated substrates and indifference towards the protonation state of the product.

We have also identified an additional migration path, the *re* path for the co-ligands dioxygen and hydrogen peroxide. This path leads co-ligands to and from the *re* side of FAD. The co-ligands were also able to migrate through the paths identified for the phenolic ligands. The *re* path is the shortest connection to the solvent, compared to the paths on the *si* side of FAD. On the *re* side of FAD, a chloride ion present in the VAO crystal structure (PDB ID: 1VAO) is indicative of a possible oxygen binding site [53,54]. There is no evidence from binding energies calculated in our simulations that the *si* path is preferred. Our simulations were performed with oxidised FAD, and while dioxygen reacts with reduced FAD, there are no indications of significant conformational changes in VAO crystal structures linked to the changed oxidation state of FAD (PDB ID: 1VAO vs PDB ID: 1AHU). All these observations make the *re* path the most probable migration path for dioxygen and hydrogen peroxide. In this path, it is possible that Tyr51 is involved in directing dioxygen to the FAD, as it is one of the most contacted and flexible residues in this path. It is also located in the probable oxygen-binding pocket on the *re* side of FAD, making it a candidate for assisting in reduced flavin oxidation.

From our results of the co-ligand simulations on the *si* side of FAD, we can see that co-ligands tend to migrate into the cap path. Crystallographic data (PDB ID: 1VAO) show that within the cap path, water molecules are located close to Arg312 and Arg398 as well as at the paths exit/entry on the surface of VAO. Water is able to access the active site and to participate in the reaction on the *si* side of FAD, reacting with the quinone methide of certain substrates [20]. The cap path is the only path at the *si* side of FAD directly connected to the solvent. In the case of binding of alkylphenols with hydrophobic aliphatic side chains, already bound water molecules would likely be expulsed from the active site via the cap path. This would explain why these substrates are dehydrogenated and not hydroxylated by VAO [55].

### Structure and sequence conservation in VAO, EUGO and PCMH

We will now discuss our findings in VAO in the context of the close bacterial relatives EUGO and PCMH. All three enzymes require their substrates to be *para*-substituted phenols [6–10].

In PCMH and EUGO, a possible route for substrate access to the active site via the subunit interface has been suggested purely on crystallographic evidence [8,12]. In this route, three residues (Glu177, Met180 and Asp434 in PCMH) have been proposed to be involved. These residues are Asp192, Met195 and Glu464 in VAO, with Asp192 being in the position of Glu177 and Glu464 in the position of Asp434. EUGO has recently had its crystal structure solved [12]. In EUGO, all the above-mentioned residues involved in the subunit interface path of VAO are also present (Fig 6).

We could show that residues at the subunit interface and in the subunit interface path are the most similar in all three of these closely related members of the VAO/PCMH family, an indication that increased selective pressure is in place for these residues. Additionally, the combination of this increased selective pressure and the presence of the entrance/exit migration path at the subunit interface (and absence of evidence of allosteric regulation in VAO) could indicate that dimerisation of VAO is functionally linked to ligand migration. Residues in the cap path are most diverse in all three enzymes. This lends support to the proposal that all three enzymes prefer the subunit interface path for ligand migration. The small amount of PCMH-like sequences remaining in the analysis after applying our selection criteria makes interpretation of the data with respect to PCMH less reliable.

VAO and EUGO re-oxidise their FAD cofactor using molecular oxygen, while PCMH requires a cytochrome c subunit for this. This functional difference is reflected in the preservation of the *re* path in these enzymes (see specifically the PCMH-unlike category in Fig 9B). Interestingly, dissimilarity in the residues of this path is only due to PCMH-like sequences and not due to differences between the two oxidases. It is noteworthy that the *re* path we identify here is located in a visible channel in VAO and EUGO. In PCMH, the channel is absent and the cytochrome *c* subunit in PCMH is located where dioxygen would exit the protein (see Fig 6B). Evolution of the *re* path in these oxidases may thus have coincided with loss of the cytochrome *c* subunit.

The strong conservation of residues in the FAD path could indicate that these residues are essential to the function of all three enzymes. It has been established that maturation of the VAO holoenzyme involves the initial non-covalent binding of the FAD cofactor to the already folded dimeric apoenzyme [11,56]. It could be that FAD binds to the apoenzyme via this path. A possible scenario is that FAD enters the enzyme with the isoalloxazine moiety first and then binds to the enzyme in the correct orientation already. The alternative scenario, in which FAD binds with its ADP moiety first, would require reorientation of the FAD inside the enzyme to fit the isoalloxazine moiety into the active site. From the crystal structure, it is not evident how this would happen as the free space around bound FAD is rather limited. Initial binding of FAD to the enzyme surface and migration of FAD into its binding pocket is likely guided by *pi* stacking interactions with the three tryptophans located in the FAD path (Trp98, Trp549 and Trp558).

### What is known about (co-)ligand migration in the entire VAO/PCMH family?

Very little is known about substrate access to the active site for the other members of this flavoprotein family. Dioxygen migration has been studied in alditol oxidase, berberine bridge enzyme (also called (*S*)-reticuline oxidase) and L-galactono-1,4-lactone dehydrogenase. Ala113 in L-galactono-1,4-lactone dehydrogenase was identified as a gatekeeper residue that prevents the enzyme from functioning as an oxidase [57]. Berberine bridge enzyme also contains such a gatekeeper residue. The variant G164A showed 800-fold decreased oxygen reactivity [58]. In alditol oxidase funnel shaped paths have been identified through molecular dynamics simulations, leading dioxygen to the “gatekeeper” Ala105 in alditol oxidase and from there to N1/N3 of the flavin ring [23]. This path leads to the *re* side of FAD, as does the *re* path we identified in this study. Our path, however, leads to its destination via the dimethylbenzene part of the flavin ring. We have therefore identified for the first time a substrate migration channel, the subunit interface path, and a novel co-substrate migration path.

In summary, we have carried out an exhaustive computational analysis of ligand migration pathways in VAO (see Fig 10 for an overview). We have identified a gated entry and exit path at the subunit interface of VAO for small phenolic ligands. The residues forming a portal in this path (Met192, Met195 and Glu464 in VAO) are conserved in PCMH and EUGO. Two residues, His466 and Tyr503 in VAO, act as concierges, obstructing the path to the active site for phenolic substrates once substrate is bound, as has been postulated before for PCMH [8]. An additional, different entry and exit path for dioxygen and hydrogen peroxide at the interface of the two domains of VAO has been identified (*re* path). Tyr51 in this path could assist reduced flavin oxidation in VAO. The only path identified directly connecting the *si* side of FAD to the solvent (the cap path) could be facilitating water access to the active site. Overall, our study illustrates the ability of ligand simulation techniques to advance the mechanistic understanding of enzyme function.

## Supporting information

S1 Movie Supports this study by providing the true four-dimensional character of the processes that we can only describe in two dimensions in the main article. (RAR)

S1 Fig Three-dimensional structures of VAO, EUGO and PCMH. Proteins (PDB IDs: 1VAO, 5FXE, 1DII) are shown as cartoons. The cap domain is coloured in red, the FAD-binding domain in green and FAD is shown as yellow sticks. A: Octameric VAO, seen from the front. B: Dimeric VAO as seen in Fig 1. C: Octameric VAO seen from the side. D: Dimeric EUGO, using the same colouring scheme as for VAO. E: Tetrameric PCMH, using the same colouring scheme as for VAO, with the cytochrome *c* subunit coloured in blue.
(TIFF)

S2 Fig **Overview of the exit paths identified with VAP and VAD (A), RMSF and atom contacts (B for VAP, C for VAD) and binding energies (D).** A: The VAO dimer is shown as cartoon in grey and its FAD cofactor is shown as sticks in yellow. The paths identified in this work are shown through sphere representation of ligand positions in the trajectories. The cap path is coloured in orange, the FAD path in blue and the subunit interface path in magenta. B: Plot of simulations with VAP, highest RMSF values (black boxplot boxes) and atom contact counts with the ligand (grey boxplot boxes) of residues in VAO. Coloured filling of the boxplots indicates that the residue belongs to the path of that colour. C: Plot of simulations with VAD, highest RMSF values (black boxplot boxes) and atom contact counts with the ligand (grey boxplot boxes) of residues in VAO. Coloured filling of the boxplots indicates that the residue belongs to the path of that colour. D: Scatterplot of binding energies as a function of the distance between the VAP and VAD (in green and black) to FAD.
(TIFF)

S3 Fig **Overview of the exit paths identified with VNP and VND (A), RMSF and atom contacts (B for VNP, C for VND) and binding energies (D).** A: The VAO dimer is shown as cartoon in grey and its FAD cofactor is shown as sticks in yellow. The paths identified in this work are shown through sphere representation of ligand positions in the trajectories. The cap path is coloured in orange, the FAD path in blue and the subunit interface path in magenta. B: Plot of simulations with VNP, highest RMSF values (black boxplot boxes) and atom contact counts with the ligand (grey boxplot boxes) of residues in VAO. Coloured filling of the boxplots indicates that the residue belongs to the path of that colour. C: Plot of simulations with VND, highest RMSF values (black boxplot boxes) and atom contact counts with the ligand (grey boxplot boxes) of residues in VAO. Coloured filling of the boxplots indicates that the residue belongs to the path of that colour. D: Scatterplot of binding energies as a function of the distance between the VNP and VND (in green and black) to FAD.
(TIFF)

S4 Fig Detailed view of the portal connecting the subunit interface to the active site formed by Asp192, Met195 and Glu464. Asp192, Met195 and Glu464 are shown as spheres (with carbons in violet) within dimeric VAO (in grey) with FAD shown as sticks (in yellow). A: The dimer as cartoon from the front. B: The dimer seen from the top as mesh.
(TIFF)

S1 Data Parameters for all the ligands used in this study. (DOCX)

S1 Alignment Multiple sequence alignment of VAO-like sequences used in this study (MSA_VAO_, fasta file). (FA)

S2 Alignment Multiple sequence alignment of EUGO-like sequences used in this study (MSA_EUGO_, fasta file). (FA)

S3 Alignment Multiple sequence alignment of PCMH-like sequences used in this study (MSA_PCMH_, fasta file). (FA)

S4 Alignment Multiple sequence alignment of all sequences used in this study (MSA_all_, fasta file). (FA)
