# Supplementary figures and images for: The ins and outs of vanillyl alcohol oxidase: Identification of ligand migration paths
Source: PLoS Comput Biol. 2017 Oct 6;13(10):e1005787. doi: 10.1371/journal.pcbi.1005787 (PMC5646868; doi:10.1371/journal.pcbi.1005787)

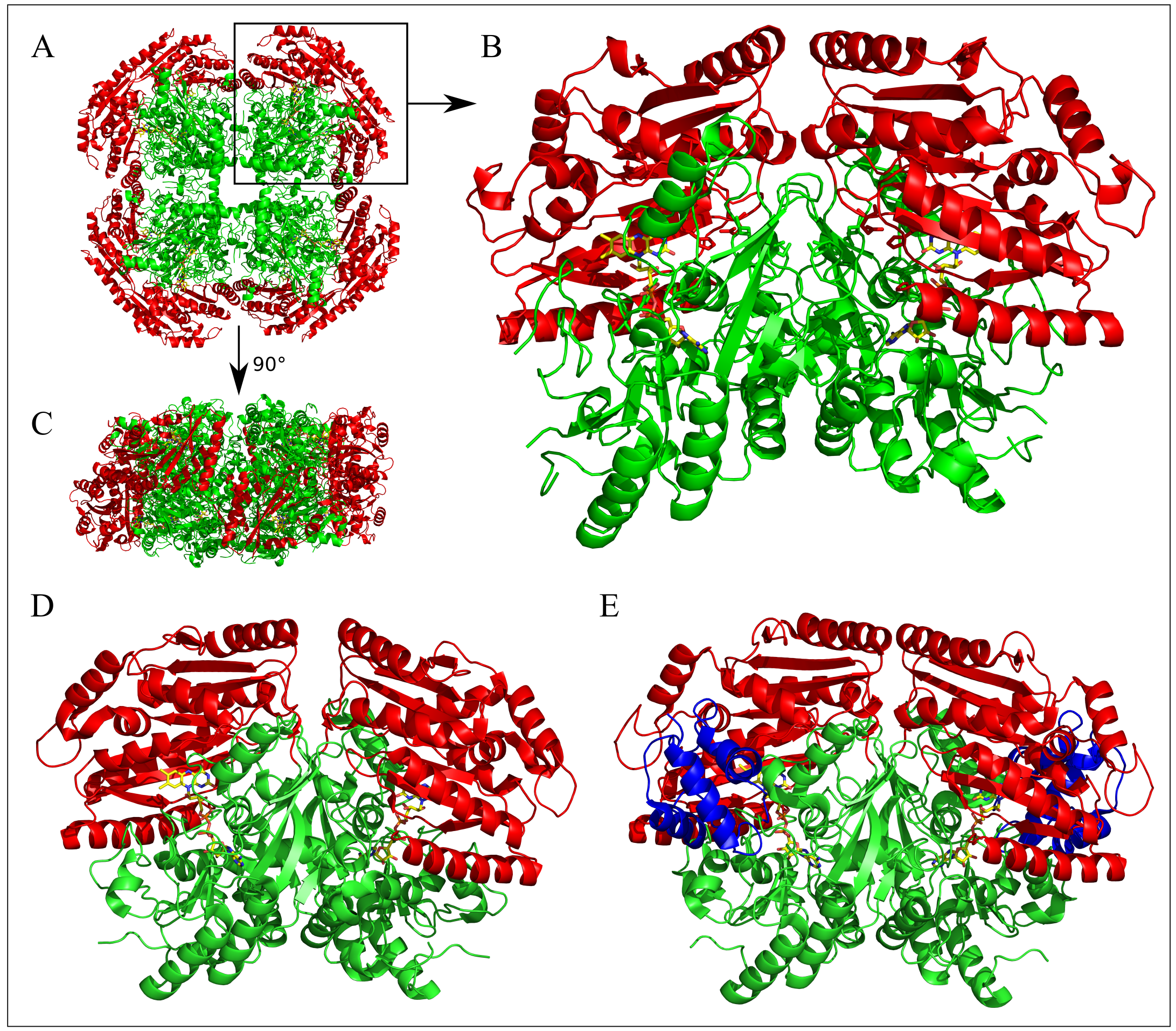

Supplement: S1 Fig — Proteins (PDB IDs: 1VAO, 5FXE, 1DII) are shown as cartoons. The cap domain is coloured in red, the FAD-binding domain in green and FAD is shown as yellow sticks. A: Octameric VAO, seen from the front. B: Dimeric VAO as seen in Fig 1. C: Octameric VAO seen from the side. D: Dimeric EUGO, using the same colouring scheme as for VAO. E: Tetrameric PCMH, using the same colouring scheme as for VAO, with the cytochrome c subunit coloured in blue. (TIFF) [file pcbi.1005787.s002.tiff]

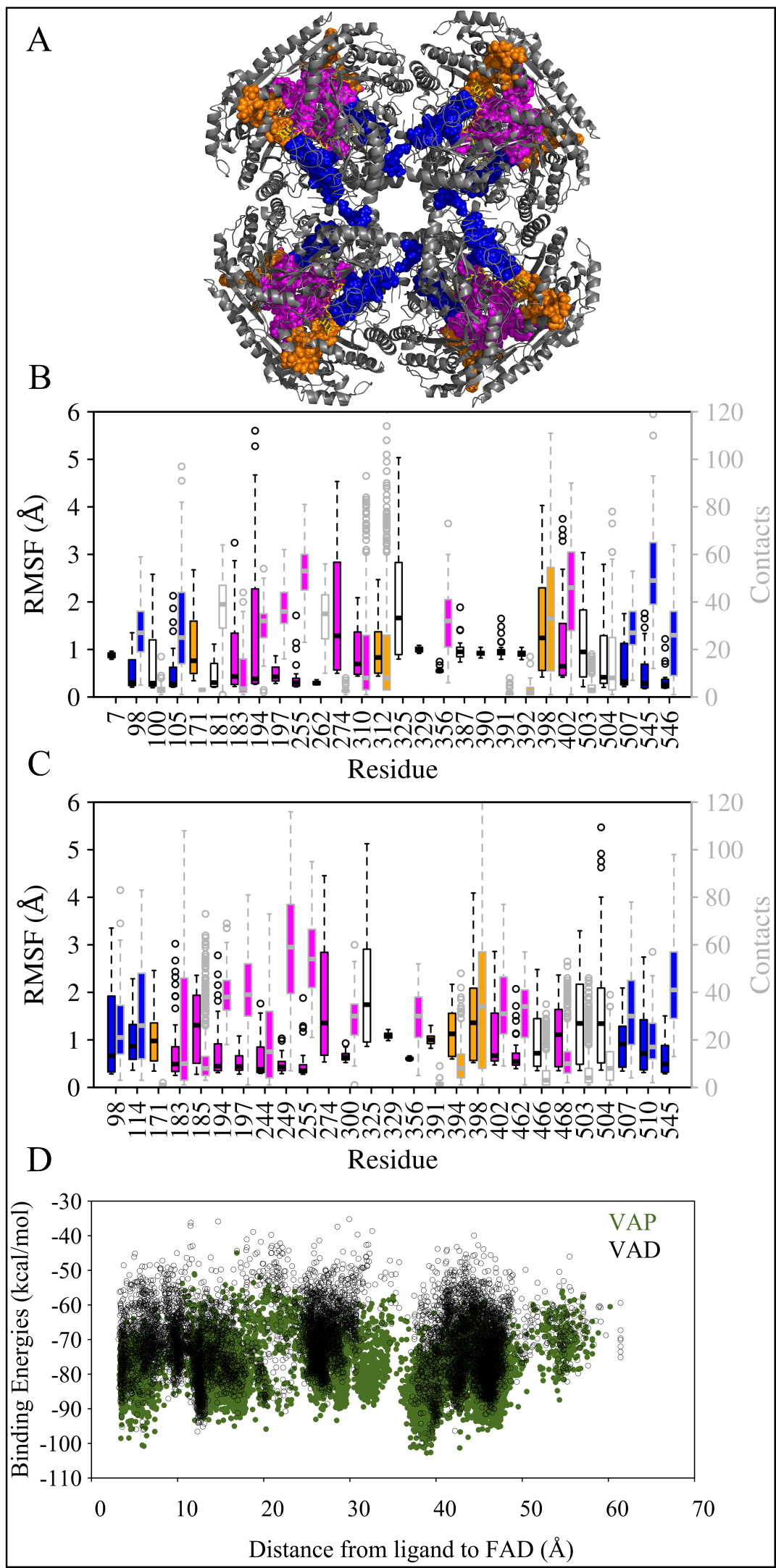

Supplement: S2 Fig — Overview of the exit paths identified with VAP and VAD (A), RMSF and atom contacts (B for VAP, C for VAD) and binding energies (D). A: The VAO dimer is shown as cartoon in grey and its FAD cofactor is shown as sticks in yellow. The paths identified in this work are shown through sphere representation of ligand positions in the trajectories. The cap path is coloured in orange, the FAD path in blue and the subunit interface path in magenta. B: Plot of simulations with VAP, highest RMSF values (black boxplot boxes) and atom contact counts with the ligand (grey boxplot boxes) of residues in VAO. Coloured filling of the boxplots indicates that the residue belongs to the path of that colour. C: Plot of simulations with VAD, highest RMSF values (black boxplot boxes) and atom contact counts with the ligand (grey boxplot boxes) of residues in VAO. Coloured filling of the boxplots indicates that the residue belongs to the path of that colour. D: Scatterplot of binding energies as a function of the distance between the VAP and VAD (in green and black) to FAD. (TIFF) [file pcbi.1005787.s003.tiff]

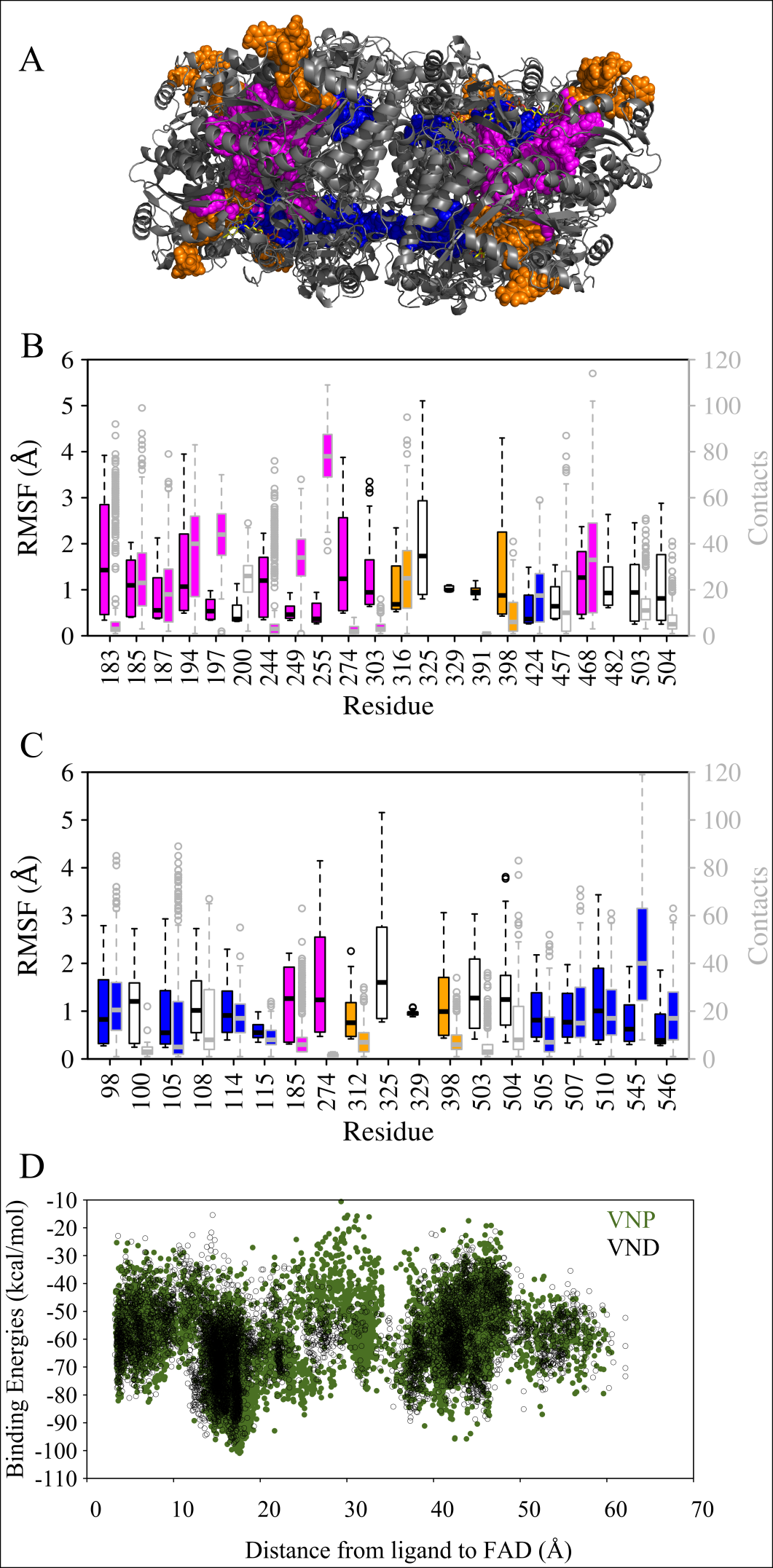

Supplement: S3 Fig — Overview of the exit paths identified with VNP and VND (A), RMSF and atom contacts (B for VNP, C for VND) and binding energies (D). A: The VAO dimer is shown as cartoon in grey and its FAD cofactor is shown as sticks in yellow. The paths identified in this work are shown through sphere representation of ligand positions in the trajectories. The cap path is coloured in orange, the FAD path in blue and the subunit interface path in magenta. B: Plot of simulations with VNP, highest RMSF values (black boxplot boxes) and atom contact counts with the ligand (grey boxplot boxes) of residues in VAO. Coloured filling of the boxplots indicates that the residue belongs to the path of that colour. C: Plot of simulations with VND, highest RMSF values (black boxplot boxes) and atom contact counts with the ligand (grey boxplot boxes) of residues in VAO. Coloured filling of the boxplots indicates that the residue belongs to the path of that colour. D: Scatterplot of binding energies as a function of the distance between the VNP and VND (in green and black) to FAD. (TIFF) [file pcbi.1005787.s004.tiff]

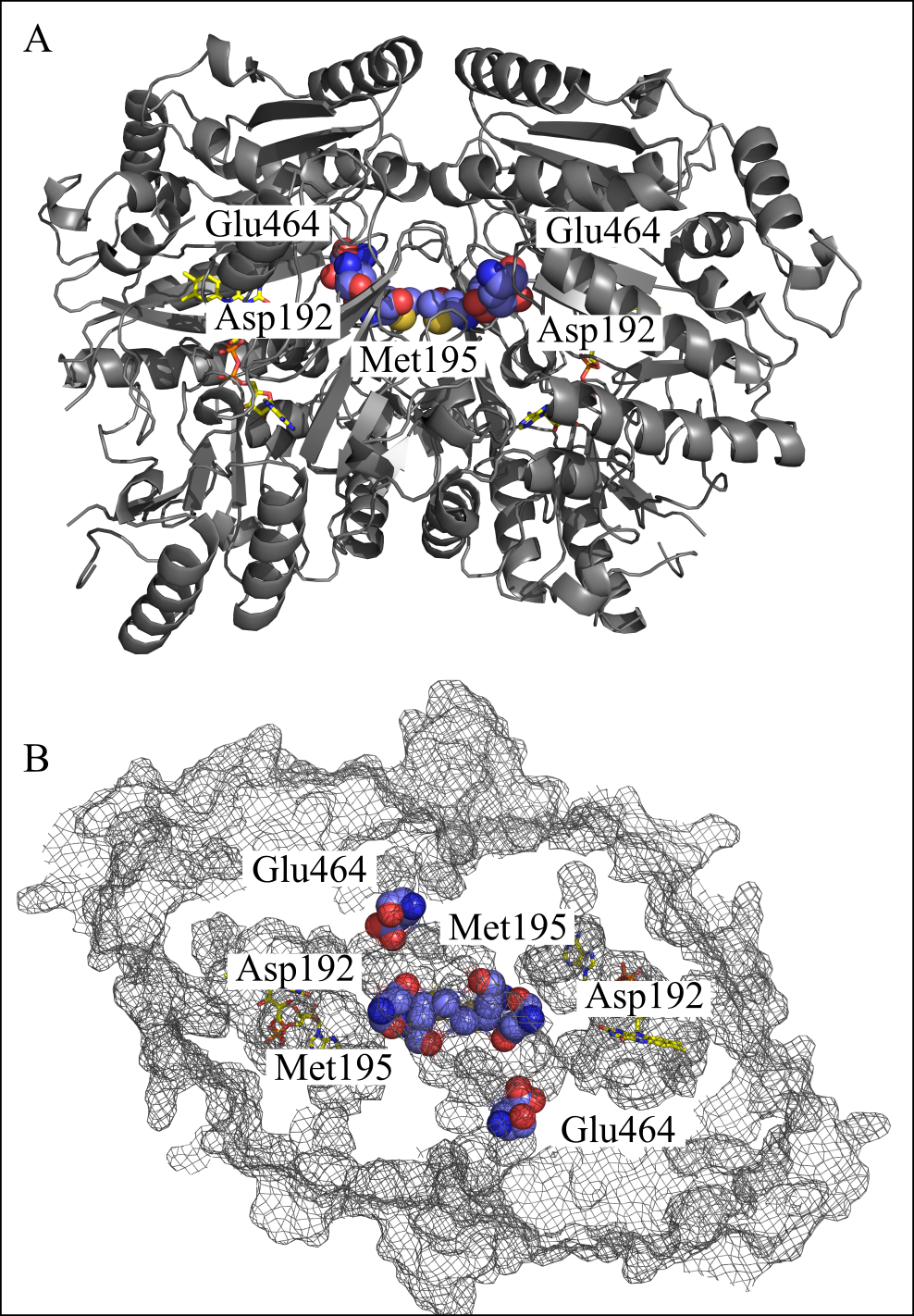

Supplement: S4 Fig — Asp192, Met195 and Glu464 are shown as spheres (with carbons in violet) within dimeric VAO (in grey) with FAD shown as sticks (in yellow). A: The dimer as cartoon from the front. B: The dimer seen from the top as mesh. (TIFF) [file pcbi.1005787.s005.tiff]
