# Supplementary material for: The ins and outs of vanillyl alcohol oxidase: Identification of ligand migration paths
Source: PLoS Comput Biol. 2017 Oct 6;13(10):e1005787. doi: 10.1371/journal.pcbi.1005787 (PMC5646868; doi:10.1371/journal.pcbi.1005787)
Supplement: S1 Data — (DOCX) [file pcbi.1005787.s006.docx]

**Supplementary Information for:**

The ins and outs of vanillyl alcohol oxidase: Identification of ligand migration paths

By: Gudrun Gygli, Maria Fátima Lucas, Victor Guallar and Willem J.H. van Berkel

Below template files for all substrate molecules used in this work as well as the FAD molecule and the covalently bound histidine. Partial charges are shown in the highlighted column.

* LIGAND DATABASE FILE (OPLS2005)

*

COD 19 19 31 41 82

1 0 M CA _C1_ 1 4.015790 106.814730 -169.999760

2 1 M CA _C2_ 2 1.392050 157.200990 167.214080

3 1 M CA _C6_ 6 1.404820 55.798050 -114.441900

4 1 M CT _C7_ 7 1.506470 69.313010 42.572980

5 2 M CA _C3_ 3 1.396290 121.473090 65.341050

6 2 M HA _H1_ 11 1.093070 119.007610 -114.658950

7 5 M CA _C4_ 4 1.420090 123.555240 0.000000

8 5 M HA _H2_ 12 1.091460 119.649850 -180.000000

9 7 M CA _C5_ 5 1.439550 113.473990 0.000000

10 7 M OM _O1_ 8 1.294310 124.046860 -180.000000

11 3 M HA _H3_ 13 1.089830 118.069920 25.201290

12 4 M HC _H7_ 17 1.097160 111.429780 -22.112930

13 4 M HC _H8_ 18 1.099260 112.540740 97.918590

14 4 M HC _H9_ 19 1.099260 112.540740 -142.144440

15 9 S OS _O2_ 9 1.381090 114.247320 -180.000000

16 15 S CT _C8_ 10 1.409010 116.858470 180.000000

17 16 S HC _H4_ 14 1.093080 107.128750 180.000000

18 16 S HC _H5_ 15 1.100640 111.964490 -60.751220

19 16 S HC _H6_ 16 1.100640 111.964490 60.751220

13 12 12 7 6 2 6 2 7 1 1 2 1 1 4 3 2

1 1

2 5 7 9 3 4 15 6 8 11 12 13 14

5 7 9 3 4 10 6 8 11 12 13 14

6 4 12 13 14 5 7 10 9 15 16 11

6 11 12 13 14 5 9

6 7 9 10 15 8

8 7

8 9 10 15 16 11

10 9

10 15 16 11 17 18 19

15

15

13 14

14

0

16 17 18 19

17 18 19

18 19

19

0

NBON

1 3.5500 0.0700 0.154520 2.5000 1.7750 0.001000000 -0.843144165

2 3.5500 0.0700 -0.305300 2.0020 1.7750 0.001000000 -0.843144165

3 3.5500 0.0700 -0.433860 2.0020 1.7750 0.001000000 -0.843144165

4 3.5000 0.0660 -0.343120 1.9750 1.7500 0.005000000 -0.741685710

5 3.5500 0.0700 -0.359950 2.0020 1.7750 0.001000000 -0.843144165

6 2.4200 0.0300 0.156930 1.3810 1.2100 0.030040813 0.268726247

7 3.5500 0.0700 0.348960 2.5000 1.7750 0.001000000 -0.843144165

8 2.4200 0.0300 0.141080 1.3810 1.2100 0.030040813 0.268726247

9 3.5500 0.0700 0.235260 2.5000 1.7750 0.001000000 -0.843144165

10 3.1500 0.2500 -0.863740 1.7825 1.5750 0.001000000 -0.126889456

11 2.4200 0.0300 0.178320 1.3810 1.2100 0.030040813 0.268726247

12 2.5000 0.0300 0.094890 1.4250 1.2500 0.008598240 0.268726247

13 2.5000 0.0300 0.093800 1.4250 1.2500 0.008598240 0.268726247

14 2.5000 0.0300 0.093800 1.4250 1.2500 0.008598240 0.268726247

15 2.9000 0.1400 -0.401180 1.7660 1.5600 0.020110767 -0.896042159

16 3.5000 0.0660 0.021640 1.9750 1.7500 0.005000000 -0.741685710

17 2.5000 0.0300 0.083170 1.4250 1.2500 0.008598240 0.268726247

18 2.5000 0.0300 0.052400 1.4250 1.2500 0.008598240 0.268726247

19 2.5000 0.0300 0.052400 1.4250 1.2500 0.008598240 0.268726247

BOND

1 2 469.000 1.400

1 3 469.000 1.400

1 4 317.000 1.510

2 5 469.000 1.400

2 6 367.000 1.080

5 7 469.000 1.400

5 8 367.000 1.080

7 9 469.000 1.400

7 10 450.000 1.364

9 3 469.000 1.400

9 15 450.000 1.364

3 11 367.000 1.080

4 12 340.000 1.090

4 13 340.000 1.090

4 14 340.000 1.090

15 16 320.000 1.410

16 17 340.000 1.090

16 18 340.000 1.090

16 19 340.000 1.090

THET

1 2 5 63.00000 120.00000

1 2 6 35.00000 120.00000

1 3 9 63.00000 120.00000

1 3 11 35.00000 120.00000

1 4 12 35.00000 109.50000

1 4 13 35.00000 109.50000

1 4 14 35.00000 109.50000

2 5 7 63.00000 120.00000

2 5 8 35.00000 120.00000

5 7 9 63.00000 120.00000

5 7 10 70.00000 120.00000

7 9 3 63.00000 120.00000

7 9 15 70.00000 120.00000

9 3 11 35.00000 120.00000

9 15 16 75.00000 111.00000

3 1 2 63.00000 120.00000

4 1 2 70.00000 120.00000

4 1 3 70.00000 120.00000

10 7 9 70.00000 120.00000

15 9 3 70.00000 120.00000

15 16 17 35.00000 109.50000

15 16 18 35.00000 109.50000

15 16 19 35.00000 109.50000

6 2 5 35.00000 120.00000

8 5 7 35.00000 120.00000

18 16 17 33.00000 107.80000

19 16 17 33.00000 107.80000

19 16 18 33.00000 107.80000

13 4 12 33.00000 107.80000

14 4 12 33.00000 107.80000

14 4 13 33.00000 107.80000

PHI

1 2 5 7 3.62500 -1.0 2.0

1 2 5 8 3.62500 -1.0 2.0

2 1 3 9 3.62500 -1.0 2.0

2 1 3 11 3.62500 -1.0 2.0

2 1 4 12 0.00000 1.0 1.0

2 1 4 13 0.00000 1.0 1.0

2 1 4 14 0.00000 1.0 1.0

2 5 -7 9 3.62500 -1.0 2.0

2 5 7 10 3.62500 -1.0 2.0

5 7 9 3 3.62500 -1.0 2.0

5 7 9 15 3.62500 -1.0 2.0

7 9 -3 1 3.62500 -1.0 2.0

7 9 3 11 3.62500 -1.0 2.0

7 9 15 16 1.95800 -1.0 2.0

9 15 16 17 0.08050 1.0 3.0

9 15 16 18 0.08050 1.0 3.0

9 15 16 19 0.08050 1.0 3.0

3 1 -2 5 3.62500 -1.0 2.0

3 1 2 6 3.62500 -1.0 2.0

3 1 4 12 0.00000 1.0 1.0

3 1 4 13 0.00000 1.0 1.0

3 1 4 14 0.00000 1.0 1.0

3 9 15 16 1.95800 -1.0 2.0

4 1 2 5 3.62500 -1.0 2.0

4 1 2 6 3.62500 -1.0 2.0

4 1 3 9 3.62500 -1.0 2.0

4 1 3 11 3.62500 -1.0 2.0

10 7 9 3 3.62500 -1.0 2.0

10 7 9 15 3.62500 -1.0 2.0

15 9 3 1 3.62500 -1.0 2.0

15 9 3 11 3.62500 -1.0 2.0

6 2 5 7 3.62500 -1.0 2.0

6 2 5 8 3.62500 -1.0 2.0

8 5 7 9 3.62500 -1.0 2.0

8 5 7 10 3.62500 -1.0 2.0

IPHI

3 4 1 2 4.00000 -1.0 2.0

5 6 2 1 1.10000 -1.0 2.0

7 8 5 2 1.10000 -1.0 2.0

9 10 7 5 4.00000 -1.0 2.0

3 15 9 7 4.00000 -1.0 2.0

9 11 3 1 1.10000 -1.0 2.0

END

* LIGAND DATABASE FILE (OPLS2005)

*

COP 20 20 32 43 86

1 0 M CA _C1_ 1 2.849650 106.090450 174.217510

2 1 M CA _C2_ 2 1.397800 73.456300 -109.250200

3 1 M CA _C6_ 6 1.403990 148.956230 132.727090

4 1 M CT _C7_ 7 1.512320 56.106510 38.390060

5 2 M CA _C3_ 3 1.396180 121.223510 -148.821460

6 2 M HA _H1_ 8 1.086890 119.590990 31.178540

7 5 M CA _C4_ 4 1.390230 120.122620 0.000000

8 5 M HA _H2_ 9 1.085180 121.387030 -180.000000

9 7 M CA _C5_ 5 1.406420 119.245400 -0.000000

10 3 M HA _H3_ 12 1.084850 119.321290 -74.226970

11 4 M HC _H8_ 18 1.093900 111.498350 143.282700

12 4 M HC _H9_ 19 1.096260 111.484180 -96.486200

13 4 M HC _H10 20 1.096260 111.484180 23.051590

14 7 S OH _O1_ 10 1.364420 120.288820 180.000000

15 14 S HO _H4_ 13 0.969600 106.810550 -180.000000

16 9 S OS _O2_ 11 1.377110 113.608660 -180.000000

17 16 S CT _C8_ 14 1.418310 118.234020 -180.000000

18 17 S HC _H5_ 15 1.091120 106.265420 -180.000000

19 17 S HC _H6_ 16 1.097260 111.405400 -61.043720

20 17 S HC _H7_ 17 1.097260 111.405400 61.043720

13 12 12 7 7 2 7 2 8 1 2 1 1 2 1 4 3

2 1 1

2 5 7 9 3 4 6 8 16 10 11 12 13

5 7 9 3 4 6 8 14 10 11 12 13

6 4 11 12 13 5 7 9 14 16 10 17

6 10 11 12 13 5 9

6 7 9 8 14 16 15

8 7

8 9 14 16 10 15 17

14 9

14 16 10 15 17 18 19 20

16

12 13

13

0

16 15

0

17 18 19 20

18 19 20

19 20

20

0

NBON

1 3.5500 0.0700 -0.115000 2.5000 1.7750 0.001000000 -0.843144165

2 3.5500 0.0700 -0.115000 2.0020 1.7750 0.001000000 -0.843144165

3 3.5500 0.0700 -0.115000 2.0020 1.7750 0.001000000 -0.843144165

4 3.5000 0.0660 -0.065000 1.9750 1.7500 0.005000000 -0.741685710

5 3.5500 0.0700 -0.115000 2.0020 1.7750 0.001000000 -0.843144165

6 2.4200 0.0300 0.115000 1.3810 1.2100 0.030040813 0.268726247

7 3.5500 0.0700 0.150000 2.0020 1.7750 0.001000000 -0.843144165

8 2.4200 0.0300 0.115000 1.3810 1.2100 0.030040813 0.268726247

9 3.5500 0.0700 0.085000 2.5000 1.7750 0.001000000 -0.843144165

10 2.4200 0.0300 0.115000 1.3810 1.2100 0.030040813 0.268726247

11 2.5000 0.0300 0.060000 1.4250 1.2500 0.008598240 0.268726247

12 2.5000 0.0300 0.060000 1.4250 1.2500 0.008598240 0.268726247

13 2.5000 0.0300 0.060000 1.4250 1.2500 0.008598240 0.268726247

14 3.0700 0.1700 -0.585000 1.7660 1.5600 0.021068034 -0.322181743

15 0.5000 0.0300 0.435000 0.9960 0.8600 0.030040813 -0.651083722

16 2.9000 0.1400 -0.285000 1.7660 1.5600 0.020110767 -0.896042159

17 3.5000 0.0660 0.110000 1.9750 1.7500 0.005000000 -0.741685710

18 2.5000 0.0300 0.030000 1.4250 1.2500 0.008598240 0.268726247

19 2.5000 0.0300 0.030000 1.4250 1.2500 0.008598240 0.268726247

20 2.5000 0.0300 0.030000 1.4250 1.2500 0.008598240 0.268726247

BOND

1 2 469.000 1.400

1 3 469.000 1.400

1 4 317.000 1.510

2 5 469.000 1.400

2 6 367.000 1.080

5 7 469.000 1.400

5 8 367.000 1.080

7 9 469.000 1.400

7 14 450.000 1.364

9 3 469.000 1.400

9 16 450.000 1.364

3 10 367.000 1.080

4 11 340.000 1.090

4 12 340.000 1.090

4 13 340.000 1.090

14 15 553.000 0.945

16 17 320.000 1.410

17 18 340.000 1.090

17 19 340.000 1.090

17 20 340.000 1.090

THET

1 2 5 63.00000 120.00000

1 2 6 35.00000 120.00000

1 3 9 63.00000 120.00000

1 3 10 35.00000 120.00000

1 4 11 35.00000 109.50000

1 4 12 35.00000 109.50000

1 4 13 35.00000 109.50000

2 5 7 63.00000 120.00000

2 5 8 35.00000 120.00000

5 7 9 63.00000 120.00000

5 7 14 70.00000 120.00000

7 9 3 63.00000 120.00000

7 9 16 70.00000 120.00000

7 14 15 35.00000 113.00000

9 3 10 35.00000 120.00000

9 16 17 75.00000 111.00000

3 1 2 63.00000 120.00000

4 1 2 70.00000 120.00000

4 1 3 70.00000 120.00000

6 2 5 35.00000 120.00000

8 5 7 35.00000 120.00000

14 7 9 70.00000 120.00000

16 9 3 70.00000 120.00000

16 17 18 35.00000 109.50000

16 17 19 35.00000 109.50000

16 17 20 35.00000 109.50000

19 17 18 33.00000 107.80000

20 17 18 33.00000 107.80000

20 17 19 33.00000 107.80000

12 4 11 33.00000 107.80000

13 4 11 33.00000 107.80000

13 4 12 33.00000 107.80000

PHI

1 2 5 7 3.62500 -1.0 2.0

1 2 5 8 3.62500 -1.0 2.0

2 1 3 9 3.62500 -1.0 2.0

2 1 3 10 3.62500 -1.0 2.0

2 1 4 11 0.00000 1.0 1.0

2 1 4 12 0.00000 1.0 1.0

2 1 4 13 0.00000 1.0 1.0

2 5 -7 9 3.62500 -1.0 2.0

2 5 7 14 3.62500 -1.0 2.0

5 7 9 3 3.62500 -1.0 2.0

5 7 9 16 3.62500 -1.0 2.0

5 7 14 15 0.84100 -1.0 2.0

7 9 -3 1 3.62500 -1.0 2.0

7 9 3 10 3.62500 -1.0 2.0

7 9 16 17 1.95800 -1.0 2.0

9 7 14 15 0.84100 -1.0 2.0

9 16 17 18 0.08050 1.0 3.0

9 16 17 19 0.08050 1.0 3.0

9 16 17 20 0.08050 1.0 3.0

3 1 -2 5 3.62500 -1.0 2.0

3 1 2 6 3.62500 -1.0 2.0

3 1 4 11 0.00000 1.0 1.0

3 1 4 12 0.00000 1.0 1.0

3 1 4 13 0.00000 1.0 1.0

3 9 16 17 1.95800 -1.0 2.0

4 1 2 5 3.62500 -1.0 2.0

4 1 2 6 3.62500 -1.0 2.0

4 1 3 9 3.62500 -1.0 2.0

4 1 3 10 3.62500 -1.0 2.0

6 2 5 7 3.62500 -1.0 2.0

6 2 5 8 3.62500 -1.0 2.0

8 5 7 9 3.62500 -1.0 2.0

8 5 7 14 3.62500 -1.0 2.0

14 7 9 3 3.62500 -1.0 2.0

14 7 9 16 3.62500 -1.0 2.0

16 9 3 1 3.62500 -1.0 2.0

16 9 3 10 3.62500 -1.0 2.0

IPHI

3 4 1 2 4.00000 -1.0 2.0

5 6 2 1 1.10000 -1.0 2.0

7 8 5 2 1.10000 -1.0 2.0

9 14 7 5 4.00000 -1.0 2.0

3 16 9 7 4.00000 -1.0 2.0

9 10 3 1 1.10000 -1.0 2.0

END

* LIGAND DATABASE FILE (OPLS2005)

*

VAD 20 20 32 44 87

1 0 M CA _C2_ 2 2.900133 102.726631 -179.105004

2 1 M CA _C1_ 1 1.394220 86.328799 -122.749318

3 2 M CA _C6_ 6 1.398280 119.034327 -150.748894

4 1 M CA _C3_ 3 1.393340 141.212691 99.224453

5 1 M HA _H1_ 8 1.090610 43.465564 19.090134

6 4 M CA _C4_ 4 1.387770 120.041970 128.882547

7 4 M HA _H2_ 9 1.089140 121.119380 -51.117453

8 6 M CA _C5_ 5 1.403260 119.390170 0.000000

9 6 M OM _O1_ 10 1.363330 119.955390 -180.000000

10 3 M HA _H3_ 12 1.086630 119.314680 -180.000000

11 2 S CT _C7_ 7 1.509610 118.469191 29.251117

12 11 S OH _O3_ 17 1.413970 111.711330 -180.000000

13 11 S HC _H8_ 19 1.103650 108.676400 -57.335850

14 11 S HC _H9_ 20 1.103650 108.676400 57.335840

15 12 S HO _H7_ 18 0.970490 107.596400 -180.000000

16 8 S OS _O2_ 11 1.362980 113.778650 180.000000

17 16 S CT _C8_ 13 1.416370 118.236170 -180.000000

18 17 S HC _H4_ 14 1.092670 106.412710 -180.000000

19 17 S HC _H5_ 15 1.098260 111.237870 -61.041590

20 17 S HC _H6_ 16 1.098260 111.237870 61.041590

13 13 12 7 3 6 2 8 1 2 4 3 2 1 1 4 3

2 1 1

2 4 6 8 3 11 5 7 9 10 12 13 14

4 6 8 3 11 5 7 16 10 12 15 13 14

5 11 13 14 4 12 6 9 8 16 10 17

5 11 6 8 7 9 16

7 11 6

7 8 9 16 10 17

9 8

11 9 16 10 17 18 19 20

16

11 16

12 15 13 14

13 14 15

14 15

15

0

17 18 19 20

18 19 20

19 20

20

0

NBON

1 3.5500 0.0700 -0.325440 2.0020 1.7750 0.001000000 -0.843144165

2 3.5500 0.0700 0.087160 2.5000 1.7750 0.001000000 -0.843144165

3 3.5500 0.0700 -0.298210 2.0020 1.7750 0.001000000 -0.843144165

4 3.5500 0.0700 -0.184850 2.0020 1.7750 0.001000000 -0.843144165

5 2.4200 0.0300 0.166260 1.3810 1.2100 0.030040813 0.268726247

6 3.5500 0.0700 0.192690 2.5000 1.7750 0.001000000 -0.843144165

7 2.4200 0.0300 0.174580 1.3810 1.2100 0.030040813 0.268726247

8 3.5500 0.0700 0.198920 2.5000 1.7750 0.001000000 -0.843144165

9 3.1500 0.2500 -0.597340 1.7825 1.5750 0.001000000 -0.126889456

10 2.4200 0.0300 0.167800 1.3810 1.2100 0.030040813 0.268726247

11 3.5000 0.0660 0.257980 1.9750 1.7500 0.005000000 -0.741685710

12 3.1200 0.1700 -0.718950 1.7660 1.5600 0.021068034 -0.322181743

13 2.5000 0.0300 0.017500 1.4250 1.2500 0.008598240 0.268726247

14 2.5000 0.0300 0.017500 1.4250 1.2500 0.008598240 0.268726247

15 0.5000 0.0300 0.472940 0.9960 0.8600 0.030040813 -0.651083722

16 2.9000 0.1400 -0.273610 1.7660 1.5600 0.020110767 -0.896042159

17 3.5000 0.0660 -0.164910 1.9750 1.7500 0.005000000 -0.741685710

18 2.5000 0.0300 0.136180 1.4250 1.2500 0.008598240 0.268726247

19 2.5000 0.0300 0.114830 1.4250 1.2500 0.008598240 0.268726247

20 2.5000 0.0300 0.114830 1.4250 1.2500 0.008598240 0.268726247

BOND

2 1 469.000 1.400

2 3 469.000 1.400

2 11 317.000 1.510

1 4 469.000 1.400

1 5 367.000 1.080

4 6 469.000 1.400

4 7 367.000 1.080

6 8 469.000 1.400

6 9 450.000 1.364

8 3 469.000 1.400

8 16 450.000 1.364

3 10 367.000 1.080

11 12 320.000 1.410

11 13 340.000 1.090

11 14 340.000 1.090

16 17 320.000 1.410

17 18 340.000 1.090

17 19 340.000 1.090

17 20 340.000 1.090

12 15 553.000 0.945

THET

2 1 4 63.00000 120.00000

2 1 5 35.00000 120.00000

2 3 8 63.00000 120.00000

2 3 10 35.00000 120.00000

2 11 12 90.00000 112.51600

2 11 13 35.00000 109.50000

2 11 14 35.00000 109.50000

1 4 6 63.00000 120.00000

1 4 7 35.00000 120.00000

4 6 8 63.00000 120.00000

4 6 9 70.00000 120.00000

6 8 3 63.00000 120.00000

6 8 16 70.00000 120.00000

8 3 10 35.00000 120.00000

8 16 17 75.00000 111.00000

3 2 1 63.00000 120.00000

11 2 1 70.00000 120.00000

11 2 3 70.00000 120.00000

11 12 15 55.00000 108.50000

5 1 4 35.00000 120.00000

7 4 6 35.00000 120.00000

9 6 8 70.00000 120.00000

16 8 3 70.00000 120.00000

16 17 18 35.00000 109.50000

16 17 19 35.00000 109.50000

16 17 20 35.00000 109.50000

19 17 18 33.00000 107.80000

20 17 18 33.00000 107.80000

20 17 19 33.00000 107.80000

13 11 12 35.00000 109.50000

14 11 12 35.00000 109.50000

14 11 13 33.00000 107.80000

PHI

2 1 4 6 3.62500 -1.0 2.0

2 1 4 7 3.62500 -1.0 2.0

2 11 12 15 0.31900 1.0 1.0

2 11 12 15 -0.11800 -1.0 2.0

2 11 12 15 0.38750 1.0 3.0

1 2 3 8 3.62500 -1.0 2.0

1 2 3 10 3.62500 -1.0 2.0

1 2 11 12 0.14300 -1.0 2.0

1 2 11 12 0.00300 1.0 3.0

1 2 11 13 0.00000 1.0 1.0

1 2 11 14 0.00000 1.0 1.0

1 4 -6 8 3.62500 -1.0 2.0

1 4 6 9 3.62500 -1.0 2.0

4 6 8 3 3.62500 -1.0 2.0

4 6 8 16 3.62500 -1.0 2.0

6 8 -3 2 3.62500 -1.0 2.0

6 8 3 10 3.62500 -1.0 2.0

6 8 16 17 1.95800 -1.0 2.0

8 16 17 18 0.08050 1.0 3.0

8 16 17 19 0.08050 1.0 3.0

8 16 17 20 0.08050 1.0 3.0

3 2 -1 4 3.62500 -1.0 2.0

3 2 1 5 3.62500 -1.0 2.0

3 2 11 12 0.14300 -1.0 2.0

3 2 11 12 0.00300 1.0 3.0

3 2 11 13 0.00000 1.0 1.0

3 2 11 14 0.00000 1.0 1.0

3 8 16 17 1.95800 -1.0 2.0

11 2 1 4 3.62500 -1.0 2.0

11 2 1 5 3.62500 -1.0 2.0

11 2 3 8 3.62500 -1.0 2.0

11 2 3 10 3.62500 -1.0 2.0

5 1 4 6 3.62500 -1.0 2.0

5 1 4 7 3.62500 -1.0 2.0

7 4 6 8 3.62500 -1.0 2.0

7 4 6 9 3.62500 -1.0 2.0

9 6 8 3 3.62500 -1.0 2.0

9 6 8 16 3.62500 -1.0 2.0

16 8 3 2 3.62500 -1.0 2.0

16 8 3 10 3.62500 -1.0 2.0

13 11 12 15 0.19450 1.0 3.0

14 11 12 15 0.19450 1.0 3.0

IPHI

3 11 2 1 4.00000 -1.0 2.0

4 5 1 2 1.10000 -1.0 2.0

6 7 4 1 1.10000 -1.0 2.0

8 9 6 4 4.00000 -1.0 2.0

3 16 8 6 4.00000 -1.0 2.0

8 10 3 2 1.10000 -1.0 2.0

END

* LIGAND DATABASE FILE (OPLS2005)

*

VAP 21 21 33 46 91

1 0 M CA _C2_ 2 2.900133 102.726631 -179.105004

2 1 M CA _C1_ 1 1.394220 86.328799 -122.749318

3 2 M CA _C6_ 6 1.398280 119.034327 -150.748894

4 1 M CA _C3_ 3 1.393340 141.212691 99.224453

5 1 M HA _H1_ 8 1.090610 43.465564 19.090134

6 4 M CA _C4_ 4 1.387770 120.041970 128.882547

7 4 M HA _H2_ 9 1.089140 121.119380 -51.117453

8 6 M CA _C5_ 5 1.403260 119.390170 0.000000

9 3 M HA _H3_ 12 1.086630 119.314680 -180.000000

10 2 S CT _C7_ 7 1.509610 118.469191 29.251117

11 10 S OH _O3_ 18 1.413970 111.711330 -180.000000

12 10 S HC _H9_ 20 1.103650 108.676400 -57.335850

13 10 S HC _H10 21 1.103650 108.676400 57.335840

14 11 S HO _H8_ 19 0.970490 107.596400 -180.000000

15 6 S OH _O1_ 10 1.363330 119.955390 -180.000000

16 15 S HO _H4_ 13 0.973660 109.241500 180.000000

17 8 S OS _O2_ 11 1.362980 113.778650 180.000000

18 17 S CT _C8_ 14 1.416370 118.236170 -180.000000

19 18 S HC _H5_ 15 1.092670 106.412710 -180.000000

20 18 S HC _H6_ 16 1.098260 111.237870 -61.041590

21 18 S HC _H7_ 17 1.098260 111.237870 61.041590

13 13 12 8 3 7 2 9 2 4 3 2 1 1 2 1 4

3 2 1 1

2 4 6 8 3 10 5 7 15 9 11 12 13

4 6 8 3 10 5 7 17 9 11 14 12 13

5 10 12 13 4 11 6 8 15 17 9 18

5 10 6 8 7 15 17 16

7 10 6

7 8 15 17 9 16 18

15 8

10 15 17 9 16 18 19 20 21

10 17

11 14 12 13

12 13 14

13 14

14

0

17 16

0

18 19 20 21

19 20 21

20 21

21

0

NBON

1 3.5500 0.0700 -0.325440 2.0020 1.7750 0.001000000 -0.843144165

2 3.5500 0.0700 0.087160 2.5000 1.7750 0.001000000 -0.843144165

3 3.5500 0.0700 -0.298210 2.0020 1.7750 0.001000000 -0.843144165

4 3.5500 0.0700 -0.184850 2.0020 1.7750 0.001000000 -0.843144165

5 2.4200 0.0300 0.166260 1.3810 1.2100 0.030040813 0.268726247

6 3.5500 0.0700 0.192690 2.0020 1.7750 0.001000000 -0.843144165

7 2.4200 0.0300 0.174580 1.3810 1.2100 0.030040813 0.268726247

8 3.5500 0.0700 0.198920 2.5000 1.7750 0.001000000 -0.843144165

9 2.4200 0.0300 0.167800 1.3810 1.2100 0.030040813 0.268726247

10 3.5000 0.0660 0.257980 1.9750 1.7500 0.005000000 -0.741685710

11 3.1200 0.1700 -0.718950 1.7660 1.5600 0.021068034 -0.322181743

12 2.5000 0.0300 0.017500 1.4250 1.2500 0.008598240 0.268726247

13 2.5000 0.0300 0.017500 1.4250 1.2500 0.008598240 0.268726247

14 0.5000 0.0300 0.472940 0.9960 0.8600 0.030040813 -0.651083722

15 3.0700 0.1700 -0.597340 1.7660 1.5600 0.021068034 -0.322181743

16 0.5000 0.0300 0.444150 0.9960 0.8600 0.030040813 -0.651083722

17 2.9000 0.1400 -0.273610 1.7660 1.5600 0.020110767 -0.896042159

18 3.5000 0.0660 -0.164910 1.9750 1.7500 0.005000000 -0.741685710

19 2.5000 0.0300 0.136180 1.4250 1.2500 0.008598240 0.268726247

20 2.5000 0.0300 0.114830 1.4250 1.2500 0.008598240 0.268726247

21 2.5000 0.0300 0.114830 1.4250 1.2500 0.008598240 0.268726247

BOND

2 1 469.000 1.400

2 3 469.000 1.400

2 10 317.000 1.510

1 4 469.000 1.400

1 5 367.000 1.080

4 6 469.000 1.400

4 7 367.000 1.080

6 8 469.000 1.400

6 15 450.000 1.364

8 3 469.000 1.400

8 17 450.000 1.364

3 9 367.000 1.080

10 11 320.000 1.410

10 12 340.000 1.090

10 13 340.000 1.090

15 16 553.000 0.945

17 18 320.000 1.410

18 19 340.000 1.090

18 20 340.000 1.090

18 21 340.000 1.090

11 14 553.000 0.945

THET

2 1 4 63.00000 120.00000

2 1 5 35.00000 120.00000

2 3 8 63.00000 120.00000

2 3 9 35.00000 120.00000

2 10 11 90.00000 112.51600

2 10 12 35.00000 109.50000

2 10 13 35.00000 109.50000

1 4 6 63.00000 120.00000

1 4 7 35.00000 120.00000

4 6 8 63.00000 120.00000

4 6 15 70.00000 120.00000

6 8 3 63.00000 120.00000

6 8 17 70.00000 120.00000

6 15 16 35.00000 113.00000

8 3 9 35.00000 120.00000

8 17 18 75.00000 111.00000

3 2 1 63.00000 120.00000

10 2 1 70.00000 120.00000

10 2 3 70.00000 120.00000

10 11 14 55.00000 108.50000

5 1 4 35.00000 120.00000

7 4 6 35.00000 120.00000

15 6 8 70.00000 120.00000

17 8 3 70.00000 120.00000

17 18 19 35.00000 109.50000

17 18 20 35.00000 109.50000

17 18 21 35.00000 109.50000

20 18 19 33.00000 107.80000

21 18 19 33.00000 107.80000

21 18 20 33.00000 107.80000

12 10 11 35.00000 109.50000

13 10 11 35.00000 109.50000

13 10 12 33.00000 107.80000

PHI

2 1 4 6 3.62500 -1.0 2.0

2 1 4 7 3.62500 -1.0 2.0

2 10 11 14 0.31900 1.0 1.0

2 10 11 14 -0.11800 -1.0 2.0

2 10 11 14 0.38750 1.0 3.0

1 2 3 8 3.62500 -1.0 2.0

1 2 3 9 3.62500 -1.0 2.0

1 2 10 11 0.14300 -1.0 2.0

1 2 10 11 0.00300 1.0 3.0

1 2 10 12 0.00000 1.0 1.0

1 2 10 13 0.00000 1.0 1.0

1 4 -6 8 3.62500 -1.0 2.0

1 4 6 15 3.62500 -1.0 2.0

4 6 8 3 3.62500 -1.0 2.0

4 6 8 17 3.62500 -1.0 2.0

4 6 15 16 0.84100 -1.0 2.0

6 8 -3 2 3.62500 -1.0 2.0

6 8 3 9 3.62500 -1.0 2.0

6 8 17 18 1.95800 -1.0 2.0

8 6 15 16 0.84100 -1.0 2.0

8 17 18 19 0.08050 1.0 3.0

8 17 18 20 0.08050 1.0 3.0

8 17 18 21 0.08050 1.0 3.0

3 2 -1 4 3.62500 -1.0 2.0

3 2 1 5 3.62500 -1.0 2.0

3 2 10 11 0.14300 -1.0 2.0

3 2 10 11 0.00300 1.0 3.0

3 2 10 12 0.00000 1.0 1.0

3 2 10 13 0.00000 1.0 1.0

3 8 17 18 1.95800 -1.0 2.0

10 2 1 4 3.62500 -1.0 2.0

10 2 1 5 3.62500 -1.0 2.0

10 2 3 8 3.62500 -1.0 2.0

10 2 3 9 3.62500 -1.0 2.0

5 1 4 6 3.62500 -1.0 2.0

5 1 4 7 3.62500 -1.0 2.0

7 4 6 8 3.62500 -1.0 2.0

7 4 6 15 3.62500 -1.0 2.0

15 6 8 3 3.62500 -1.0 2.0

15 6 8 17 3.62500 -1.0 2.0

17 8 3 2 3.62500 -1.0 2.0

17 8 3 9 3.62500 -1.0 2.0

12 10 11 14 0.19450 1.0 3.0

13 10 11 14 0.19450 1.0 3.0

IPHI

3 10 2 1 4.00000 -1.0 2.0

4 5 1 2 1.10000 -1.0 2.0

6 7 4 1 1.10000 -1.0 2.0

8 15 6 4 4.00000 -1.0 2.0

3 17 8 6 4.00000 -1.0 2.0

8 9 3 2 1.10000 -1.0 2.0

END

* LIGAND DATABASE FILE (OPLS2005)

*

VND 18 18 28 40 76

1 0 M CA _C2_ 2 3.064368 116.227442 142.323089

2 1 M CA _C1_ 1 1.405080 77.217801 83.574396

3 2 M CA _C6_ 6 1.418560 118.414709 151.755930

4 1 M CA _C3_ 3 1.379200 148.232770 -146.797288

5 1 M HA _H1_ 8 1.092380 49.699899 -63.469015

6 4 M CA _C4_ 4 1.431000 122.603550 -118.765641

7 4 M HA _H2_ 9 1.089810 120.908130 61.234359

8 6 M CA _C5_ 5 1.456220 114.909680 -0.000000

9 6 M OM _O1_ 10 1.270190 123.474750 -180.000000

10 3 M HA _H3_ 12 1.088040 117.378450 -180.000000

11 2 S CO4 _C7_ 7 1.434090 120.449883 -28.244062

12 11 S O _O3_ 17 1.237290 126.682500 -180.000000

13 11 S HC _H7_ 18 1.115380 113.836570 0.000000

14 8 S OS _O2_ 11 1.370380 113.528550 -180.000000

15 14 S CT _C8_ 13 1.411200 116.547750 180.000000

16 15 S HC _H4_ 14 1.092350 106.989600 -180.000000

17 15 S HC _H5_ 15 1.099930 111.661320 -60.706860

18 15 S HC _H6_ 16 1.099930 111.661320 60.706860

12 11 11 7 3 6 2 8 1 2 2 1 1 4 3 2 1

1

2 4 6 8 3 11 5 7 9 10 12 13

4 6 8 3 11 5 7 14 10 12 13

5 11 12 13 4 6 9 8 14 10 15

5 11 6 8 7 9 14

7 11 6

7 8 9 14 10 15

9 8

11 9 14 10 15 16 17 18

14

11 14

12 13

13

0

15 16 17 18

16 17 18

17 18

18

0

NBON

1 3.5500 0.0700 -0.185410 2.0020 1.7750 0.001000000 -0.843144165

2 3.5500 0.0700 -0.202540 2.5000 1.7750 0.001000000 -0.843144165

3 3.5500 0.0700 -0.199980 2.0020 1.7750 0.001000000 -0.843144165

4 3.5500 0.0700 -0.352040 2.0020 1.7750 0.001000000 -0.843144165

5 2.4200 0.0300 0.159200 1.3810 1.2100 0.030040813 0.268726247

6 3.5500 0.0700 0.463890 2.5000 1.7750 0.001000000 -0.843144165

7 2.4200 0.0300 0.141020 1.3810 1.2100 0.030040813 0.268726247

8 3.5500 0.0700 0.167460 2.5000 1.7750 0.001000000 -0.843144165

9 3.1500 0.2500 -0.787180 1.7825 1.5750 0.001000000 -0.126889456

10 2.4200 0.0300 0.137730 1.3810 1.2100 0.030040813 0.268726247

11 3.7500 0.1050 0.475400 2.1120 1.8750 0.001000000 -0.126889456

12 2.9600 0.2100 -0.623400 1.6780 1.4800 0.001000000 -0.126889456

13 2.4200 0.0150 -0.030880 1.3810 1.2100 0.008598240 0.268726247

14 2.9000 0.1400 -0.384990 1.7660 1.5600 0.020110767 -0.896042159

15 3.5000 0.0660 0.013050 1.9750 1.7500 0.005000000 -0.741685710

16 2.5000 0.0300 0.091700 1.4250 1.2500 0.008598240 0.268726247

17 2.5000 0.0300 0.058480 1.4250 1.2500 0.008598240 0.268726247

18 2.5000 0.0300 0.058480 1.4250 1.2500 0.008598240 0.268726247

BOND

2 1 469.000 1.400

2 3 469.000 1.400

2 11 310.000 1.470

1 4 469.000 1.400

1 5 367.000 1.080

4 6 469.000 1.400

4 7 367.000 1.080

6 8 469.000 1.400

6 9 450.000 1.364

8 3 469.000 1.400

8 14 450.000 1.364

3 10 367.000 1.080

11 12 570.000 1.229

11 13 340.000 1.090

14 15 320.000 1.410

15 16 340.000 1.090

15 17 340.000 1.090

15 18 340.000 1.090

THET

2 1 4 63.00000 120.00000

2 1 5 35.00000 120.00000

2 3 8 63.00000 120.00000

2 3 10 35.00000 120.00000

2 11 12 75.00000 123.78400

2 11 13 35.00000 115.00000

1 4 6 63.00000 120.00000

1 4 7 35.00000 120.00000

4 6 8 63.00000 120.00000

4 6 9 70.00000 120.00000

6 8 3 63.00000 120.00000

6 8 14 70.00000 120.00000

8 3 10 35.00000 120.00000

8 14 15 75.00000 111.00000

3 2 1 63.00000 120.00000

11 2 1 60.00000 119.52600

11 2 3 60.00000 119.52600

5 1 4 35.00000 120.00000

7 4 6 35.00000 120.00000

9 6 8 70.00000 120.00000

14 8 3 70.00000 120.00000

14 15 16 35.00000 109.50000

14 15 17 35.00000 109.50000

14 15 18 35.00000 109.50000

17 15 16 33.00000 107.80000

18 15 16 33.00000 107.80000

18 15 17 33.00000 107.80000

13 11 12 35.00000 123.00000

PHI

2 1 4 6 3.62500 -1.0 2.0

2 1 4 7 3.62500 -1.0 2.0

1 2 3 8 3.62500 -1.0 2.0

1 2 3 10 3.62500 -1.0 2.0

1 2 11 12 1.05000 -1.0 2.0

1 2 11 13 1.00000 -1.0 2.0

1 4 -6 8 3.62500 -1.0 2.0

1 4 6 9 3.62500 -1.0 2.0

4 6 8 3 3.62500 -1.0 2.0

4 6 8 14 3.62500 -1.0 2.0

6 8 -3 2 3.62500 -1.0 2.0

6 8 3 10 3.62500 -1.0 2.0

6 8 14 15 1.95800 -1.0 2.0

8 14 15 16 0.08050 1.0 3.0

8 14 15 17 0.08050 1.0 3.0

8 14 15 18 0.08050 1.0 3.0

3 2 -1 4 3.62500 -1.0 2.0

3 2 1 5 3.62500 -1.0 2.0

3 2 11 12 1.05000 -1.0 2.0

3 2 11 13 1.00000 -1.0 2.0

3 8 14 15 1.95800 -1.0 2.0

11 2 1 4 3.62500 -1.0 2.0

11 2 1 5 3.62500 -1.0 2.0

11 2 3 8 3.62500 -1.0 2.0

11 2 3 10 3.62500 -1.0 2.0

5 1 4 6 3.62500 -1.0 2.0

5 1 4 7 3.62500 -1.0 2.0

7 4 6 8 3.62500 -1.0 2.0

7 4 6 9 3.62500 -1.0 2.0

9 6 8 3 3.62500 -1.0 2.0

9 6 8 14 3.62500 -1.0 2.0

14 8 3 2 3.62500 -1.0 2.0

14 8 3 10 3.62500 -1.0 2.0

IPHI

3 11 2 1 4.00000 -1.0 2.0

4 5 1 2 1.10000 -1.0 2.0

6 7 4 1 1.10000 -1.0 2.0

8 9 6 4 4.00000 -1.0 2.0

3 14 8 6 4.00000 -1.0 2.0

8 10 3 2 1.10000 -1.0 2.0

2 13 11 12 10.50000 -1.0 2.0

END

* LIGAND DATABASE FILE (OPLS2005)

*

VNP 19 19 29 42 80

1 0 M CA _C2_ 2 99.411233 59.252189 -41.951782

2 1 M CA _C1_ 1 1.404880 136.359926 -113.464201

3 2 M CA _C6_ 6 1.418380 118.430472 -125.552603

4 1 M CA _C3_ 3 1.379460 88.275489 110.774546

5 1 M HA _H1_ 12 1.092460 45.589870 -26.382699

6 4 M CA _C4_ 4 1.431500 122.566160 145.781207

7 4 M HA _H2_ 13 1.088760 120.941770 -34.164753

8 6 M CA _C5_ 5 1.455980 114.903230 0.048460

9 3 M HA _H3_ 14 1.088570 117.398300 179.991961

10 2 S CO4 _C7_ 7 1.433960 120.455547 54.534365

11 10 S O _O3_ 11 1.237760 126.711700 179.994797

12 10 S HC _H7_ 18 1.115900 113.823060 -0.036503

13 6 S OH _O1_ 8 1.269700 123.472920 -179.972020

14 13 S HO _H8_ 19 0.959830 109.503320 180.000000

15 8 S OS _O2_ 9 1.371050 113.536380 180.000000

16 15 S CT _C8_ 10 1.411270 116.557250 179.951540

17 16 S HC _H4_ 15 1.092290 106.938890 179.955770

18 16 S HC _H5_ 16 1.099240 111.633170 -60.701140

19 16 S HC _H6_ 17 1.100100 111.619120 60.729110

12 11 11 8 3 7 2 9 2 2 1 1 2 1 4 3 2

1 1

2 4 6 8 3 10 13 11 5 7 9 12

4 6 8 3 10 15 11 5 7 9 12

10 11 5 12 4 6 8 13 15 16 9

10 5 6 8 13 15 7 14

10 7 6

7 8 13 15 16 9 14

8 13

10 13 15 16 9 17 18 19 14

10 15

11 12

12

0

15 14

0

16 17 18 19

17 18 19

18 19

19

0

NBON

1 3.5500 0.0700 -0.115000 2.0020 1.7750 0.001000000 -0.843144165

2 3.5500 0.0700 -0.115000 2.5000 1.7750 0.001000000 -0.843144165

3 3.5500 0.0700 -0.115000 2.0020 1.7750 0.001000000 -0.843144165

4 3.5500 0.0700 -0.115000 2.0020 1.7750 0.001000000 -0.843144165

5 2.4200 0.0300 0.115000 1.3810 1.2100 0.030040813 0.268726247

6 3.5500 0.0700 0.150000 2.0020 1.7750 0.001000000 -0.843144165

7 2.4200 0.0300 0.115000 1.3810 1.2100 0.030040813 0.268726247

8 3.5500 0.0700 0.085000 2.5000 1.7750 0.001000000 -0.843144165

9 2.4200 0.0300 0.115000 1.3810 1.2100 0.030040813 0.268726247

10 3.7500 0.1050 0.565000 2.1120 1.8750 0.001000000 -0.126889456

11 2.9600 0.2100 -0.450000 1.6780 1.4800 0.001000000 -0.126889456

12 2.4200 0.0150 0.000000 1.3810 1.2100 0.008598240 0.268726247

13 3.0700 0.1700 -0.585000 1.7660 1.5600 0.021068034 -0.322181743

14 0.5000 0.0300 0.435000 0.9960 0.8600 0.030040813 -0.651083722

15 2.9000 0.1400 -0.285000 1.7660 1.5600 0.020110767 -0.896042159

16 3.5000 0.0660 0.110000 1.9750 1.7500 0.005000000 -0.741685710

17 2.5000 0.0300 0.030000 1.4250 1.2500 0.008598240 0.268726247

18 2.5000 0.0300 0.030000 1.4250 1.2500 0.008598240 0.268726247

19 2.5000 0.0300 0.030000 1.4250 1.2500 0.008598240 0.268726247

BOND

2 1 469.000 1.400

2 3 469.000 1.400

2 10 310.000 1.470

1 4 469.000 1.400

1 5 367.000 1.080

4 6 469.000 1.400

4 7 367.000 1.080

6 8 469.000 1.400

6 13 450.000 1.364

8 3 469.000 1.400

8 15 450.000 1.364

3 9 367.000 1.080

10 11 570.000 1.229

10 12 340.000 1.090

13 14 553.000 0.945

15 16 320.000 1.410

16 17 340.000 1.090

16 18 340.000 1.090

16 19 340.000 1.090

THET

2 1 4 63.00000 120.00000

2 1 5 35.00000 120.00000

2 3 8 63.00000 120.00000

2 3 9 35.00000 120.00000

2 10 11 75.00000 123.78400

2 10 12 35.00000 115.00000

1 4 6 63.00000 120.00000

1 4 7 35.00000 120.00000

4 6 8 63.00000 120.00000

4 6 13 70.00000 120.00000

6 8 3 63.00000 120.00000

6 8 15 70.00000 120.00000

6 13 14 35.00000 113.00000

8 3 9 35.00000 120.00000

8 15 16 75.00000 111.00000

3 2 1 63.00000 120.00000

10 2 1 60.00000 119.52600

10 2 3 60.00000 119.52600

13 6 8 70.00000 120.00000

15 8 3 70.00000 120.00000

15 16 17 35.00000 109.50000

15 16 18 35.00000 109.50000

15 16 19 35.00000 109.50000

5 1 4 35.00000 120.00000

7 4 6 35.00000 120.00000

18 16 17 33.00000 107.80000

19 16 17 33.00000 107.80000

19 16 18 33.00000 107.80000

12 10 11 35.00000 123.00000

PHI

2 1 4 6 3.62500 -1.0 2.0

2 1 4 7 3.62500 -1.0 2.0

1 2 3 8 3.62500 -1.0 2.0

1 2 3 9 3.62500 -1.0 2.0

1 2 10 11 1.05000 -1.0 2.0

1 2 10 12 1.00000 -1.0 2.0

1 4 -6 8 3.62500 -1.0 2.0

1 4 6 13 3.62500 -1.0 2.0

4 6 8 3 3.62500 -1.0 2.0

4 6 8 15 3.62500 -1.0 2.0

4 6 13 14 0.84100 -1.0 2.0

6 8 -3 2 3.62500 -1.0 2.0

6 8 3 9 3.62500 -1.0 2.0

6 8 15 16 1.95800 -1.0 2.0

8 6 13 14 0.84100 -1.0 2.0

8 15 16 17 0.08050 1.0 3.0

8 15 16 18 0.08050 1.0 3.0

8 15 16 19 0.08050 1.0 3.0

3 2 -1 4 3.62500 -1.0 2.0

3 2 1 5 3.62500 -1.0 2.0

3 2 10 11 1.05000 -1.0 2.0

3 2 10 12 1.00000 -1.0 2.0

3 8 15 16 1.95800 -1.0 2.0

10 2 1 4 3.62500 -1.0 2.0

10 2 1 5 3.62500 -1.0 2.0

10 2 3 8 3.62500 -1.0 2.0

10 2 3 9 3.62500 -1.0 2.0

13 6 8 3 3.62500 -1.0 2.0

13 6 8 15 3.62500 -1.0 2.0

15 8 3 2 3.62500 -1.0 2.0

15 8 3 9 3.62500 -1.0 2.0

5 1 4 6 3.62500 -1.0 2.0

5 1 4 7 3.62500 -1.0 2.0

7 4 6 8 3.62500 -1.0 2.0

7 4 6 13 3.62500 -1.0 2.0

IPHI

3 10 2 1 4.00000 -1.0 2.0

4 5 1 2 1.10000 -1.0 2.0

6 7 4 1 1.10000 -1.0 2.0

8 13 6 4 4.00000 -1.0 2.0

3 15 8 6 4.00000 -1.0 2.0

8 9 3 2 1.10000 -1.0 2.0

2 12 10 11 10.50000 -1.0 2.0

END

* LIGAND DATABASE FILE (OPLS2005)

*

FAD 84 89 153 243 444

1 0 S P1 _PA_ 1 128.29359 72.10143 -30.64046

2 1 S O2Z _O1A 2 1.48818 153.08600 109.99841

3 1 S OS _O2A 3 1.49001 54.44365 -163.11388

4 3 S HO HO2A 54 0.96000 109.50003 31.42609

5 1 S OS _O5B 4 1.59683 60.15034 41.85886

6 1 S OS _O3P 53 1.62055 96.91510 -54.03036

7 5 S CT _C5B 5 1.30538 130.61222 -40.30041

8 6 S P2 _P__ 50 1.62402 130.25431 -142.45319

9 8 S O2Z _O1P 51 1.48700 107.62731 149.66378

10 8 S O2Z _O2P 52 1.49421 108.99963 18.24207

11 7 S HC H5B1 55 1.08999 109.41314 53.60794

12 7 S HC H5B2 56 1.09000 109.41290 -70.15510

13 7 S CT _C4B 6 1.50749 106.42524 171.72675

14 13 S HC _H4B 57 1.09000 111.53793 73.96642

15 8 S OS _O5' 49 1.61410 101.54991 -96.35270

16 13 S OS _O4B 7 1.45264 102.11602 -166.79932

17 13 S CT _C3B 8 1.53258 113.69829 -53.20877

18 17 S HC _H3B 58 1.09000 112.07712 -5.61213

19 15 S CT _C5' 48 1.42020 112.70859 -84.31735

20 19 S HC H5'1 83 1.09000 109.44072 23.15812

21 19 S HC H5'2 84 1.09000 109.44111 140.91196

22 17 S OH _O3B 9 1.43527 110.55829 120.21223

23 22 S HO HO3B 59 0.96000 109.50041 67.92137

24 17 S CT _C2B 10 1.53138 102.88590 -126.20048

25 24 S HC _H2B 60 1.09000 111.49937 151.83926

26 19 S CT _C4' 46 1.52436 111.34349 -97.96468

27 26 S HC _H4' 81 1.09000 108.66714 -17.39737

28 24 S OH _O2B 11 1.41359 112.21276 -82.26419

29 28 S HO HO2B 61 0.96000 109.50022 -180.00000

30 16 S CO _C1B 12 1.40854 109.45067 108.45788

31 30 S HC _H1B 62 1.09000 108.89794 148.42607

32 26 S CT _C3' 44 1.52707 111.68414 -137.25919

33 32 S HC _H3' 79 1.08999 108.57269 -162.89345

34 26 S OH _O4' 47 1.42391 109.88567 101.38362

35 34 S HO HO4' 82 0.96000 109.49982 -35.43880

36 30 S N* _N9A 13 1.46651 113.19136 -90.30268

37 32 S CT _C2' 42 1.52534 109.48554 78.72676

38 37 S HC _H2' 77 1.09000 107.93121 -55.19416

39 32 S OH _O3' 45 1.43016 110.36522 -44.00304

40 39 S HO HO3' 80 0.96000 109.49946 167.68897

41 36 S CRAB _C8A 14 1.36748 129.05612 36.44547

42 41 S HA _H8A 63 1.08000 120.00015 -0.85707

43 37 S CT _C1' 41 1.52805 113.43006 -174.72567

44 43 S HC H1'1 75 1.09001 109.36145 49.76354

45 43 S HC H1'2 76 1.09000 109.36121 164.18719

46 37 S OH _O2' 43 1.42375 108.61162 61.57705

47 46 S HO HO2' 78 0.96000 109.49946 -105.68774

48 41 S NNA _N7A 15 1.31118 113.49783 179.14271

49 43 S NE _N10 39 1.48300 114.04111 -73.02441

50 48 S C56B _C5A 16 1.38591 103.98851 -0.54858

51 49 S CA _C9A 38 1.37300 120.26651 -92.91675

52 49 S CDX _C10 40 1.37243 120.09761 91.51859

53 50 S CA _C6A 17 1.41037 132.88213 -179.82086

54 52 S NI _N1_ 23 1.35038 120.01719 -8.03544

55 53 S NND _N6A 18 1.33719 123.55292 -0.03956

56 53 S NB _N1A 19 1.34969 117.81953 -180.00000

57 54 S C _C2_ 24 1.32424 120.81712 -174.15434

58 57 S O _O2_ 25 1.22990 119.82087 -179.42223

59 55 S H H6A1 64 1.01000 120.00021 -180.00000

60 55 S H H6A2 65 1.01001 119.99980 -0.00000

61 56 S CQ _C2A 20 1.33777 118.86545 0.02798

62 61 S HA _H2A 66 1.07999 120.00001 179.82086

63 57 S N _N3_ 26 1.39036 121.65002 0.75666

64 63 S H _HN3 67 1.01000 119.99991 -179.81655

65 61 S NB _N3A 21 1.33217 128.64903 -0.18023

66 63 S C _C4_ 27 1.38902 121.52793 0.18345

67 66 S O _O4_ 28 1.23013 120.33953 178.86771

68 36 S C56A _C4A 22 1.37164 124.88888 -145.45261

69 52 S CD _C4X 29 1.42611 118.97942 178.24654

70 69 S NI _N5_ 30 1.35061 122.15426 -1.78761

71 70 S CA _C5X 31 1.34913 118.09812 0.90590

72 71 S CA _C6_ 32 1.40025 117.56966 -178.38723

73 72 S HA _H6_ 68 1.08000 120.00010 -3.65848

74 72 S CA _C7_ 33 1.40107 120.38206 176.34189

75 74 S CT _C7M 34 1.50778 120.41451 -180.00000

76 75 S HC H7M1 69 1.09000 109.50006 180.00000

77 75 S HC H7M2 70 1.09000 109.50008 -59.99991

78 75 S HC H7M3 71 1.09000 109.49969 60.00001

79 74 S CA _C8_ 35 1.42619 119.66170 -0.51244

80 79 S CM _C8M 36 1.51295 120.30648 179.82754

81 80 S H H8M1 72 1.09000 109.21423 -124.20875

82 80 S H H8M2 73 1.09000 109.21425 -13.14448

83 79 S CA _C9_ 37 1.40177 119.51283 0.86048

84 83 S HA _H9_ 74 1.08000 120.00022 -179.51382

13 6 5 2 9 6 10 7 3 2 5 4 12 6 7 11

10 6 10 5 4 5 1 8 5 11 6 4 1 8 3 13

7 3 1 8 11 7 3 1 6 3 11 5 4 2 1 6

12 8 12 10 8 7 5 6 6 3 1 1 3 2 5 3

1 4 2 1 3 5 7 9 3 10 6 3 2 1 5 4

2 1 1 1

2 3 5 7 13 15 8 9 10 6 4 11 12

3 5 7 8 6 4

5 7 8 6 4

5 6

7 13 16 17 8 6 11 12 14

7 8 9 10 15 19

13 16 17 22 24 30 11 12 14 18

9 10 20 21 15 19 26

10 15 19

15 19

12 14 13 16 17

14 13 16 17

16 17 22 24 28 30 36 14 18 23 25 31

18 16 17 22 24 30

27 20 21 19 26 32 34

17 22 24 28 30 36 41 68 18 25 31

22 24 28 30 36 18 23 25 29 31

23 25 22 24 28 30

33 27 35 20 21 26 32 34 37 39

21 26 27 32 34

26 27 32 34

24 28 30 23 25

24

28 30 36 41 68 25 29 31

29 31 28 30 36

34 38 33 40 27 35 32 37 39 43 46

35 32 33 34 37 39

30 36 29 31

30

36 41 48 50 65 68 31 42

36 41 68

39 34 44 45 38 47 33 40 35 37 43 46 49

40 34 37 38 39 43 46

35 37 39

0

41 48 50 53 61 65 68 42

46 39 44 45 38 47 40 43 49 51 52

47 39 43 44 45 46 49

40 43 46

0

48 50 53 65 68 42

48 50 68

46 44 45 47 49 51 52 54 69 71 83

45 46 49 51 52

46 49 51 52

47 49

0

50 53 55 56 65 68

52 84 51 54 57 66 69 70 71 72 79 83

53 55 56 61 65 68 59 60

52 73 84 54 69 70 71 72 74 79 80 83

54 57 58 63 66 67 69 70 71 83

55 56 61 65 68 59 60 62

57 58 63 66 69 70 64

56 61 68 59 60

61 65 68 59 60 62

58 63 66 67 69 64

63 66 64

60

0

65 68 62

65 68

66 67 69 70 64

66 67 69

68

67 69 70 71

69 70

0

70 71 72

71 72 74 83 73

72 74 75 79 83 73 84

74 75 79 80 83 73 76 77 78

74 75 79

75 79 80 83 76 77 78 81 82 84

79 80 83 76 77 78

77 78 79

78 79

79

80 83 81 82 84

83 81 82 84

82 83

83

84

0

NBON

1 3.7400 0.2000 1.230700 2.1070 1.8700 0.005000000 0.000000000

2 2.9800 0.2000 -0.666100 1.6890 1.4900 0.001000000 -0.126889456

3 2.8500 0.1400 -0.568200 1.7660 1.5600 0.021068034 -0.322181743

4 0.5000 0.0300 0.380000 0.9960 0.8600 0.030040813 -0.651083722

5 2.8500 0.1400 -0.461100 1.7660 1.5600 0.020110767 -0.896042159

6 2.8500 0.1400 -0.500700 1.5900 1.4000 0.020110767 -0.896042159

7 3.5000 0.0660 0.152900 1.9750 1.7500 0.005000000 -0.741685710

8 3.7400 0.2000 1.662500 2.1070 1.8700 0.005000000 0.000000000

9 2.9800 0.2000 -0.970000 1.6890 1.4900 0.001000000 -0.126889456

10 2.9800 0.2000 -0.970000 1.6890 1.4900 0.001000000 -0.126889456

11 2.5000 0.0300 0.060000 1.4250 1.2500 0.008598240 0.268726247

12 2.5000 0.0300 0.060000 1.4250 1.2500 0.008598240 0.268726247

13 3.5000 0.0660 0.170000 1.9750 1.7500 0.005000000 -0.741685710

14 2.5000 0.0300 0.030000 1.4250 1.2500 0.008598240 0.268726247

15 2.8500 0.1400 -0.610000 1.5900 1.4000 0.020110767 -0.896042159

16 2.9000 0.1400 -0.400000 1.7660 1.5600 0.020110767 -0.896042159

17 3.5000 0.0660 0.205000 1.9750 1.7500 0.005000000 -0.741685710

18 2.5000 0.0300 0.060000 1.4250 1.2500 0.008598240 0.268726247

19 3.5000 0.0660 0.080000 1.9750 1.7500 0.005000000 -0.741685710

20 2.5000 0.0300 0.060000 1.4250 1.2500 0.008598240 0.268726247

21 2.5000 0.0300 0.060000 1.4250 1.2500 0.008598240 0.268726247

22 3.1200 0.1700 -0.683000 1.7660 1.5600 0.021068034 -0.322181743

23 0.5000 0.0300 0.418000 0.9960 0.8600 0.030040813 -0.651083722

24 3.5000 0.0660 0.205000 1.9750 1.7500 0.005000000 -0.741685710

25 2.5000 0.0300 0.060000 1.4250 1.2500 0.008598240 0.268726247

26 3.5000 0.0660 0.205000 1.9750 1.7500 0.005000000 -0.741685710

27 2.5000 0.0300 0.060000 1.4250 1.2500 0.008598240 0.268726247

28 3.1200 0.1700 -0.683000 1.7660 1.5600 0.021068034 -0.322181743

29 0.5000 0.0300 0.418000 0.9960 0.8600 0.030040813 -0.651083722

30 3.5000 0.0660 0.450000 1.9750 1.7500 0.005000000 -0.741685710

31 2.5000 0.0500 0.100000 1.4250 1.2500 0.008598240 0.268726247

32 3.5000 0.0660 0.205000 1.9750 1.7500 0.005000000 -0.741685710

33 2.5000 0.0300 0.060000 1.4250 1.2500 0.008598240 0.268726247

34 3.1200 0.1700 -0.683000 1.7660 1.5600 0.021068034 -0.322181743

35 0.5000 0.0300 0.418000 0.9960 0.8600 0.030040813 -0.651083722

36 3.2500 0.1700 -0.500000 1.8000 1.6000 0.001000000 -0.511443539

37 3.5000 0.0660 0.205000 1.9750 1.7500 0.005000000 -0.741685710

38 2.5000 0.0300 0.060000 1.4250 1.2500 0.008598240 0.268726247

39 3.1200 0.1700 -0.683000 1.7660 1.5600 0.021068034 -0.322181743

40 0.5000 0.0300 0.418000 0.9960 0.8600 0.030040813 -0.651083722

41 3.5000 0.0800 0.200000 2.0020 1.7750 0.001000000 -0.843144165

42 2.5000 0.0500 0.200000 1.3810 1.2100 0.030040813 0.268726247

43 3.5000 0.0660 0.002500 1.9750 1.7500 0.005000000 -0.741685710

44 2.5000 0.0150 0.060000 1.4250 1.2500 0.008598240 0.268726247

45 2.5000 0.0150 0.060000 1.4250 1.2500 0.008598240 0.268726247

46 3.1200 0.1700 -0.683000 1.7660 1.5600 0.021068034 -0.322181743

47 0.5000 0.0300 0.418000 0.9960 0.8600 0.030040813 -0.651083722

48 3.2500 0.1700 -0.490000 1.8000 1.6000 0.001000000 -0.511443539

49 3.2500 0.1700 -0.197700 1.9050 1.6860 0.051222218 -2.220205820

50 3.5000 0.0800 0.150000 2.5000 1.7750 0.001000000 -0.843144165

51 3.5500 0.0700 0.100200 2.5000 1.7750 0.001000000 -0.843144165

52 3.7500 0.1050 0.334000 2.0020 1.7750 0.023028004 -0.852763146

53 3.5000 0.0800 0.440000 2.0020 1.7750 0.001000000 -0.843144165

54 3.2800 0.1700 -0.481500 1.8540 1.6400 0.005000000 0.000000000

55 3.2500 0.1700 -0.810000 1.8650 1.6500 0.051222218 -2.220205820

56 3.2500 0.1700 -0.530000 1.8000 1.6000 0.001000000 -0.511443539

57 3.7500 0.1050 0.753000 1.9250 1.7000 0.001000000 -0.126889456

58 2.9600 0.2100 -0.508000 1.5250 1.4250 0.001000000 -1.480495490

59 0.5000 0.0300 0.370000 0.9960 0.8600 0.030040813 -0.651083722

60 0.5000 0.0300 0.370000 0.9960 0.8600 0.030040813 -0.651083722

61 3.5000 0.0800 0.220000 2.0020 1.7750 0.001000000 -0.843144165

62 2.5000 0.0500 0.200000 1.3810 1.2100 0.030040813 0.268726247

63 3.2500 0.1700 -0.536200 1.8100 1.6000 0.054801302 -1.445846770

64 0.5000 0.0300 0.291200 1.5900 1.4000 0.030040813 -0.651083722

65 3.2500 0.1700 -0.550000 1.8000 1.6000 0.001000000 -0.511443539

66 3.7500 0.1050 0.630500 1.9250 1.7000 0.001000000 -0.126889456

67 2.9600 0.2100 -0.508000 1.5250 1.4250 0.001000000 -1.480495490

68 3.5000 0.0800 0.380000 2.5000 1.7750 0.001000000 -0.843144165

69 3.7500 0.1050 0.389700 2.0020 1.7750 0.023028004 -0.852763146

70 3.2800 0.1700 -0.468500 1.8540 1.6400 0.005000000 0.000000000

71 3.5500 0.0700 0.078800 2.5000 1.7750 0.001000000 -0.843144165

72 3.5500 0.0700 -0.115000 2.0020 1.7750 0.001000000 -0.843144165

73 2.4200 0.0300 0.115000 1.3810 1.2100 0.030040813 0.268726247

74 3.5500 0.0700 -0.115000 2.5000 1.7750 0.001000000 -0.843144165

75 3.5000 0.0660 -0.065000 1.9750 1.7500 0.005000000 -0.741685710

76 2.5000 0.0300 0.060000 1.4250 1.2500 0.008598240 0.268726247

77 2.5000 0.0300 0.060000 1.4250 1.2500 0.008598240 0.268726247

78 2.5000 0.0300 0.060000 1.4250 1.2500 0.008598240 0.268726247

79 3.5500 0.0700 0.000000 2.5000 1.7750 0.001000000 -0.843144165

80 3.5500 0.0760 -1.230000 2.0020 1.7750 0.023028004 -0.852763146

81 2.5000 0.0300 0.115000 1.4250 1.2500 0.008598240 0.268726247

82 2.5000 0.0300 0.115000 1.4250 1.2500 0.008598240 0.268726247

83 3.5500 0.0700 -0.115000 2.0020 1.7750 0.001000000 -0.843144165

84 2.4200 0.0300 0.115000 1.3810 1.2100 0.030040813 0.268726247

BOND

1 2 675.000 1.490

1 3 405.000 1.623

1 5 405.000 1.623

1 6 405.000 1.623

3 4 553.000 0.945

5 7 320.000 1.410

7 13 268.000 1.529

7 11 340.000 1.090

7 12 340.000 1.090

13 16 320.000 1.410

13 17 268.000 1.529

13 14 340.000 1.090

16 30 320.000 1.380

17 22 320.000 1.410

17 24 268.000 1.529

17 18 340.000 1.090

22 23 553.000 0.945

24 28 320.000 1.410

24 30 250.000 1.527

24 25 340.000 1.090

28 29 553.000 0.945

30 36 275.000 1.479

30 31 340.000 1.090

36 41 440.000 1.371

36 68 436.000 1.374

41 48 529.000 1.304

41 42 367.000 1.080

48 50 414.000 1.391

50 53 469.000 1.404

50 68 447.000 1.419

53 55 481.000 1.340

53 56 483.000 1.339

55 59 434.000 1.010

55 60 434.000 1.010

56 61 502.000 1.324

61 65 502.000 1.324

61 62 367.000 1.080

65 68 461.000 1.354

54 57 415.000 1.368

54 52 650.000 1.285

57 58 570.000 1.229

57 63 490.000 1.335

63 66 490.000 1.335

63 64 434.000 1.010

66 67 570.000 1.229

66 69 270.000 1.486

69 70 500.000 1.261

69 52 270.000 1.479

70 71 400.000 1.385

71 72 469.000 1.400

71 51 469.000 1.400

72 74 469.000 1.400

72 73 367.000 1.080

74 75 317.000 1.510

74 79 469.000 1.400

75 76 340.000 1.090

75 77 340.000 1.090

75 78 340.000 1.090

79 80 320.000 1.460

79 83 469.000 1.400

80 81 380.000 1.080

80 82 380.000 1.080

83 51 469.000 1.400

83 84 367.000 1.080

51 49 481.000 1.393

49 52 445.000 1.380

49 43 382.000 1.448

43 37 268.000 1.529

43 44 340.000 1.090

43 45 340.000 1.090

37 46 320.000 1.410

37 32 268.000 1.529

37 38 340.000 1.090

46 47 553.000 0.945

32 39 320.000 1.410

32 26 268.000 1.529

32 33 340.000 1.090

39 40 553.000 0.945

26 34 320.000 1.410

26 19 268.000 1.529

26 27 340.000 1.090

34 35 553.000 0.945

19 15 320.000 1.410

19 20 340.000 1.090

19 21 340.000 1.090

15 8 230.000 1.697

8 9 525.000 1.480

8 10 525.000 1.480

8 6 230.000 1.697

THET

1 3 4 50.00000 113.86100

1 5 7 75.00000 119.24600

1 6 8 55.00000 126.09000

3 1 2 100.00000 115.86100

5 1 2 100.00000 115.86100

5 1 3 120.00000 102.38000

5 7 13 50.00000 109.50000

5 7 11 35.00000 109.50000

5 7 12 35.00000 109.50000

7 13 16 50.00000 109.50000

7 13 17 58.35000 112.70000

7 13 14 37.50000 110.70000

13 16 30 60.00000 109.50000

13 17 22 50.00000 109.50000

13 17 24 58.35000 112.70000

13 17 18 37.50000 110.70000

16 30 24 90.00000 108.56700

16 30 36 105.00000 106.22000

16 30 31 35.00000 109.50000

17 13 16 50.00000 109.50000

17 22 23 55.00000 108.50000

17 24 28 50.00000 109.50000

17 24 30 75.00000 109.43000

17 24 25 37.50000 110.70000

24 17 22 50.00000 109.50000

24 28 29 55.00000 108.50000

24 30 36 75.00000 107.44000

24 30 31 45.00000 112.01100

30 24 28 90.00000 111.58700

30 36 41 70.00000 128.80000

30 36 68 70.00000 125.80000

36 41 48 70.00000 113.90000

36 41 42 35.00000 123.05000

36 68 50 70.00000 106.20000

36 68 65 70.00000 126.20000

41 48 50 70.00000 103.80000

48 50 53 70.00000 132.40000

48 50 68 70.00000 110.40000

50 53 55 70.00000 123.50000

50 53 56 70.00000 117.30000

50 68 65 70.00000 127.70000

53 55 59 35.00000 120.00000

53 55 60 35.00000 120.00000

53 56 61 70.00000 118.60000

56 53 55 70.00000 119.30000

56 61 65 70.00000 129.10000

56 61 62 35.00000 115.45000

61 65 68 70.00000 111.00000

68 36 41 70.00000 105.40000

68 50 53 85.00000 116.20000

54 57 58 80.00000 126.08800

54 57 63 90.00000 112.94000

54 52 69 65.00000 122.46400

54 52 49 75.00000 124.36500

57 63 66 70.00000 121.37400

57 63 64 35.00000 119.80000

63 57 58 80.00000 122.90000

63 66 67 80.00000 122.90000

63 66 69 75.00000 109.57000

66 69 70 65.00000 123.79500

66 69 52 70.00000 118.70000

69 66 67 80.00000 120.45000

69 70 71 70.00000 113.96500

69 52 49 75.00000 117.28700

70 71 72 70.00000 120.44500

70 71 51 70.00000 120.44500

71 72 74 63.00000 120.00000

71 72 73 35.00000 120.00000

71 51 83 63.00000 120.00000

71 51 49 70.00000 120.10000

72 74 75 70.00000 120.00000

72 74 79 63.00000 120.00000

74 75 76 35.00000 109.50000

74 75 77 35.00000 109.50000

74 75 78 35.00000 109.50000

74 79 80 60.00000 120.93500

74 79 83 63.00000 120.00000

79 74 75 70.00000 120.00000

79 80 81 35.00000 120.00000

79 80 82 35.00000 120.00000

79 83 51 63.00000 120.00000

79 83 84 35.00000 120.00000

83 79 80 60.00000 120.93500

83 51 49 70.00000 120.10000

51 71 72 63.00000 120.00000

51 49 52 50.00000 129.40000

51 49 43 50.00000 110.00000

49 43 37 80.00000 111.61500

49 43 44 35.00000 109.50000

49 43 45 35.00000 109.50000

52 54 57 75.00000 109.13600

52 69 70 65.00000 122.46400

43 49 52 50.00000 119.50000

43 37 46 50.00000 109.50000

43 37 32 58.35000 112.70000

43 37 38 37.50000 110.70000

37 46 47 55.00000 108.50000

37 32 39 50.00000 109.50000

37 32 26 58.35000 112.70000

37 32 33 37.50000 110.70000

32 37 46 50.00000 109.50000

32 39 40 55.00000 108.50000

32 26 34 50.00000 109.50000

32 26 19 58.35000 112.70000

32 26 27 37.50000 110.70000

26 32 39 50.00000 109.50000

26 34 35 55.00000 108.50000

26 19 15 50.00000 109.50000

26 19 20 37.50000 110.70000

26 19 21 37.50000 110.70000

19 26 34 50.00000 109.50000

19 15 8 100.00000 120.50000

15 8 9 100.00000 108.23000

15 8 10 100.00000 108.23000

15 8 6 45.00000 102.60000

10 8 9 140.00000 119.90000

6 1 2 100.00000 115.86100

6 1 3 120.00000 102.38000

6 1 5 120.00000 102.38000

6 8 9 100.00000 108.23000

6 8 10 100.00000 108.23000

11 7 13 37.50000 110.70000

12 7 13 37.50000 110.70000

12 7 11 33.00000 107.80000

14 13 16 35.00000 109.50000

14 13 17 37.50000 110.70000

18 17 22 35.00000 109.50000

18 17 24 37.50000 110.70000

25 24 28 35.00000 109.50000

25 24 30 45.00000 108.72000

31 30 36 55.00000 103.44000

42 41 48 35.00000 123.05000

60 55 59 35.00000 120.00000

62 61 65 35.00000 115.45000

64 63 66 35.00000 119.80000

73 72 74 35.00000 120.00000

77 75 76 33.00000 107.80000

78 75 76 33.00000 107.80000

78 75 77 33.00000 107.80000

82 80 81 35.00000 117.00000

84 83 51 35.00000 120.00000

44 43 37 37.50000 110.70000

45 43 37 37.50000 110.70000

45 43 44 33.00000 107.80000

38 37 46 35.00000 109.50000

38 37 32 37.50000 110.70000

33 32 39 35.00000 109.50000

33 32 26 37.50000 110.70000

27 26 34 35.00000 109.50000

27 26 19 37.50000 110.70000

20 19 15 35.00000 109.50000

21 19 15 35.00000 109.50000

21 19 20 33.00000 107.80000

PHI

1 5 7 13 -2.65550 1.0 1.0

1 5 7 13 -0.22350 -1.0 2.0

1 5 7 13 0.24150 1.0 3.0

1 5 7 11 0.03000 1.0 3.0

1 5 7 12 0.03000 1.0 3.0

2 1 3 4 0.00000 1.0 1.0

2 1 5 7 0.00000 1.0 1.0

2 1 6 8 0.50000 1.0 3.0

3 1 5 7 0.91800 1.0 1.0

3 1 5 7 0.22900 -1.0 2.0

3 1 5 7 0.32050 1.0 3.0

3 1 6 8 0.88300 1.0 1.0

3 1 6 8 0.16450 -1.0 2.0

3 1 6 8 0.32850 1.0 3.0

5 1 3 4 -0.22900 1.0 1.0

5 1 3 4 0.09450 -1.0 2.0

5 1 3 4 -0.07800 1.0 3.0

5 1 6 8 0.88300 1.0 1.0

5 1 6 8 0.16450 -1.0 2.0

5 1 6 8 0.32850 1.0 3.0

5 7 13 16 1.11900 1.0 1.0

5 7 13 16 -1.16350 -1.0 2.0

5 7 13 16 -0.34150 1.0 3.0

5 7 13 17 0.62750 1.0 1.0

5 7 13 17 -0.30950 -1.0 2.0

5 7 13 17 0.27300 1.0 3.0

5 7 13 14 0.23400 1.0 3.0

7 13 16 30 0.06250 1.0 1.0

7 13 16 30 -0.01100 -1.0 2.0

7 13 16 30 0.28050 1.0 3.0

7 13 17 22 0.86500 1.0 1.0

7 13 17 22 -0.17500 -1.0 2.0

7 13 17 22 0.20800 1.0 3.0

7 13 17 24 0.56750 1.0 1.0

7 13 17 24 -0.07550 -1.0 2.0

7 13 17 24 0.20000 1.0 3.0

7 13 17 18 0.15000 1.0 3.0

13 16 -30 24 0.06250 1.0 1.0

13 16 -30 24 -0.01100 -1.0 2.0

13 16 -30 24 0.28050 1.0 3.0

13 16 30 36 -2.37750 1.0 1.0

13 16 30 36 -0.08150 -1.0 2.0

13 16 30 36 0.59000 1.0 3.0

13 16 30 31 0.37050 1.0 3.0

13 17 22 23 -0.11600 1.0 1.0

13 17 22 23 -0.09450 -1.0 2.0

13 17 22 23 0.21250 1.0 3.0

13 17 24 28 0.86500 1.0 1.0

13 17 24 28 -0.17500 -1.0 2.0

13 17 24 28 0.20800 1.0 3.0

13 17 -24 30 3.97050 1.0 1.0

13 17 24 25 0.15000 1.0 3.0

16 13 17 22 3.69350 1.0 1.0

16 13 17 22 -1.82950 -1.0 2.0

16 13 -17 24 0.62750 1.0 1.0

16 13 -17 24 -0.30950 -1.0 2.0

16 13 -17 24 0.27300 1.0 3.0

16 13 17 18 0.23400 1.0 3.0

16 30 36 41 0.10350 1.0 1.0

16 30 36 41 -0.15350 -1.0 2.0

16 30 36 68 0.10350 1.0 1.0

16 30 36 68 -0.15350 -1.0 2.0

17 13 -16 30 0.06250 1.0 1.0

17 13 -16 30 -0.01100 -1.0 2.0

17 13 -16 30 0.28050 1.0 3.0

17 24 28 29 -0.11600 1.0 1.0

17 24 28 29 -0.09450 -1.0 2.0

17 24 28 29 0.21250 1.0 3.0

17 24 -30 16 0.62750 1.0 1.0

17 24 -30 16 -0.30950 -1.0 2.0

17 24 -30 16 0.27300 1.0 3.0

17 24 30 36 -0.64600 1.0 1.0

17 24 30 36 0.58400 -1.0 2.0

17 24 30 31 0.15000 1.0 3.0

22 17 24 28 3.75450 1.0 1.0

22 17 24 30 0.86500 1.0 1.0

22 17 24 30 -0.17500 -1.0 2.0

22 17 24 30 0.20800 1.0 3.0

22 17 24 25 0.23400 1.0 3.0

24 17 22 23 -0.11600 1.0 1.0

24 17 22 23 -0.09450 -1.0 2.0

24 17 22 23 0.21250 1.0 3.0

24 30 36 41 1.10250 1.0 1.0

24 30 36 41 0.01650 -1.0 2.0

24 30 36 41 0.39100 1.0 3.0

24 30 36 68 1.10250 1.0 1.0

24 30 36 68 0.01650 -1.0 2.0

24 30 36 68 0.39100 1.0 3.0

28 24 30 16 3.69350 1.0 1.0

28 24 30 16 -1.82950 -1.0 2.0

28 24 30 36 2.76900 1.0 1.0

28 24 30 36 -0.24350 -1.0 2.0

28 24 30 36 -0.78250 1.0 3.0

28 24 30 31 0.23400 1.0 3.0

30 24 28 29 -0.11600 1.0 1.0

30 24 28 29 -0.09450 -1.0 2.0

30 24 28 29 0.21250 1.0 3.0

30 36 41 48 3.62500 -1.0 2.0

30 36 41 42 3.62500 -1.0 2.0

30 36 68 50 0.81400 1.0 1.0

30 36 68 50 1.55550 -1.0 2.0

30 36 68 65 3.62500 -1.0 2.0

36 41 -48 50 3.62500 -1.0 2.0

41 36 -68 50 0.70250 1.0 1.0

41 36 -68 50 1.56150 -1.0 2.0

41 36 68 65 3.62500 -1.0 2.0

41 48 50 53 3.62500 -1.0 2.0

41 48 -50 68 3.62500 -1.0 2.0

48 50 53 55 3.62500 -1.0 2.0

48 50 53 56 3.62500 -1.0 2.0

48 50 -68 36 3.62500 -1.0 2.0

48 50 68 65 3.62500 -1.0 2.0

50 53 55 59 1.00000 -1.0 2.0

50 53 55 60 1.00000 -1.0 2.0

50 53 56 61 3.62500 -1.0 2.0

53 50 68 36 3.00000 -1.0 2.0

53 50 68 65 3.62500 -1.0 2.0

53 56 -61 65 3.62500 -1.0 2.0

53 56 61 62 3.62500 -1.0 2.0

55 53 56 61 3.62500 -1.0 2.0

56 53 55 59 1.00000 -1.0 2.0

56 53 55 60 1.00000 -1.0 2.0

56 61 65 68 3.62500 -1.0 2.0

61 65 68 36 3.62500 -1.0 2.0

61 65 -68 50 3.62500 -1.0 2.0

68 36 -41 48 3.62500 -1.0 2.0

68 36 41 42 3.62500 -1.0 2.0

68 50 53 55 3.62500 -1.0 2.0

68 50 -53 56 3.62500 -1.0 2.0

54 57 63 66 2.00000 -1.0 2.0

54 57 63 64 2.56700 -1.0 2.0

57 54 52 69 6.00000 -1.0 2.0

57 54 52 49 6.00000 -1.0 2.0

57 63 66 67 2.22250 -1.0 2.0

57 63 -66 69 2.45000 -1.0 2.0

58 57 63 66 2.22250 -1.0 2.0

58 57 63 64 2.45000 -1.0 2.0

63 66 69 70 1.50000 -1.0 2.0

63 66 69 52 1.50000 -1.0 2.0

66 69 70 71 6.00000 -1.0 2.0

66 69 -52 54 7.00000 -1.0 2.0

66 69 52 49 7.00000 -1.0 2.0

67 66 69 70 1.00000 -1.0 2.0

67 66 69 52 1.00000 -1.0 2.0

69 70 71 72 1.25000 -1.0 2.0

69 70 71 51 1.25000 -1.0 2.0

70 69 52 54 7.00000 -1.0 2.0

70 69 52 49 7.00000 -1.0 2.0

70 71 72 74 3.62500 -1.0 2.0

70 71 72 73 3.62500 -1.0 2.0

70 71 51 83 3.62500 -1.0 2.0

70 71 -51 49 3.62500 -1.0 2.0

71 72 74 75 3.62500 -1.0 2.0

71 72 74 79 3.62500 -1.0 2.0

71 51 49 52 -0.02750 1.0 1.0

71 51 49 52 1.00000 -1.0 2.0

71 51 49 43 -1.00000 1.0 1.0

71 51 49 43 1.25000 -1.0 2.0

72 71 51 83 3.62500 -1.0 2.0

72 71 51 49 3.62500 -1.0 2.0

72 74 75 76 0.00000 1.0 1.0

72 74 75 77 0.00000 1.0 1.0

72 74 75 78 0.00000 1.0 1.0

72 74 79 80 3.62500 -1.0 2.0

72 74 -79 83 3.62500 -1.0 2.0

74 79 80 81 -0.01750 1.0 1.0

74 79 80 81 -0.32000 -1.0 2.0

74 79 80 81 0.00600 1.0 3.0

74 79 80 82 -0.01750 1.0 1.0

74 79 80 82 -0.32000 -1.0 2.0

74 79 80 82 0.00600 1.0 3.0

74 79 83 51 3.62500 -1.0 2.0

74 79 83 84 3.62500 -1.0 2.0

75 74 79 80 3.62500 -1.0 2.0

75 74 79 83 3.62500 -1.0 2.0

79 74 75 76 0.00000 1.0 1.0

79 74 75 77 0.00000 1.0 1.0

79 74 75 78 0.00000 1.0 1.0

79 83 -51 71 3.62500 -1.0 2.0

79 83 51 49 3.62500 -1.0 2.0

80 79 83 51 3.62500 -1.0 2.0

80 79 83 84 3.62500 -1.0 2.0

83 79 80 81 -0.01750 1.0 1.0

83 79 80 81 -0.32000 -1.0 2.0

83 79 80 81 0.00600 1.0 3.0

83 79 80 82 -0.01750 1.0 1.0

83 79 80 82 -0.32000 -1.0 2.0

83 79 80 82 0.00600 1.0 3.0

83 51 49 52 -0.02750 1.0 1.0

83 51 49 52 1.00000 -1.0 2.0

83 51 49 43 -1.00000 1.0 1.0

83 51 49 43 1.25000 -1.0 2.0

51 71 -72 74 3.62500 -1.0 2.0

51 71 72 73 3.62500 -1.0 2.0

51 49 52 54 -1.45100 1.0 1.0

51 49 52 54 1.83200 -1.0 2.0

51 49 -52 69 -0.54500 1.0 1.0

51 49 -52 69 1.14150 -1.0 2.0

51 49 43 37 -0.12500 1.0 1.0

51 49 43 37 -0.12500 -1.0 2.0

51 49 43 37 0.25000 1.0 3.0

51 49 43 44 0.00000 1.0 1.0

51 49 43 45 0.00000 1.0 1.0

49 43 37 46 4.07950 1.0 1.0

49 43 37 46 -0.26450 -1.0 2.0

49 43 37 46 -0.12150 1.0 3.0

49 43 37 32 1.26750 1.0 1.0

49 43 37 32 -0.00950 -1.0 2.0

49 43 37 32 0.30600 1.0 3.0

49 43 37 38 0.22950 1.0 3.0

52 54 57 58 3.09300 -1.0 2.0

52 54 -57 63 -5.73250 1.0 1.0

52 54 -57 63 0.67550 -1.0 2.0

52 69 -70 71 6.00000 -1.0 2.0

52 49 43 37 0.38950 1.0 1.0

52 49 43 37 -0.47050 -1.0 2.0

52 49 43 37 0.83150 1.0 3.0

52 49 43 44 0.00000 1.0 1.0

52 49 43 45 0.00000 1.0 1.0

43 49 52 54 -0.86000 1.0 1.0

43 49 52 54 1.00000 -1.0 2.0

43 49 52 69 -0.54700 1.0 1.0

43 49 52 69 1.23000 -1.0 2.0

43 37 46 47 -0.11600 1.0 1.0

43 37 46 47 -0.09450 -1.0 2.0

43 37 46 47 0.21250 1.0 3.0

43 37 32 39 0.86500 1.0 1.0

43 37 32 39 -0.17500 -1.0 2.0

43 37 32 39 0.20800 1.0 3.0

43 37 32 26 0.56750 1.0 1.0

43 37 32 26 -0.07550 -1.0 2.0

43 37 32 26 0.20000 1.0 3.0

43 37 32 33 0.15000 1.0 3.0

37 32 39 40 -0.11600 1.0 1.0

37 32 39 40 -0.09450 -1.0 2.0

37 32 39 40 0.21250 1.0 3.0

37 32 26 34 0.86500 1.0 1.0

37 32 26 34 -0.17500 -1.0 2.0

37 32 26 34 0.20800 1.0 3.0

37 32 26 19 0.56750 1.0 1.0

37 32 26 19 -0.07550 -1.0 2.0

37 32 26 19 0.20000 1.0 3.0

37 32 26 27 0.15000 1.0 3.0

46 37 32 39 3.75450 1.0 1.0

46 37 32 26 0.86500 1.0 1.0

46 37 32 26 -0.17500 -1.0 2.0

46 37 32 26 0.20800 1.0 3.0

46 37 32 33 0.23400 1.0 3.0

32 37 46 47 -0.11600 1.0 1.0

32 37 46 47 -0.09450 -1.0 2.0

32 37 46 47 0.21250 1.0 3.0

32 26 34 35 -0.11600 1.0 1.0

32 26 34 35 -0.09450 -1.0 2.0

32 26 34 35 0.21250 1.0 3.0

32 26 19 15 0.62750 1.0 1.0

32 26 19 15 -0.30950 -1.0 2.0

32 26 19 15 0.27300 1.0 3.0

32 26 19 20 0.15000 1.0 3.0

32 26 19 21 0.15000 1.0 3.0

39 32 26 34 3.75450 1.0 1.0

39 32 26 19 0.86500 1.0 1.0

39 32 26 19 -0.17500 -1.0 2.0

39 32 26 19 0.20800 1.0 3.0

39 32 26 27 0.23400 1.0 3.0

26 32 39 40 -0.11600 1.0 1.0

26 32 39 40 -0.09450 -1.0 2.0

26 32 39 40 0.21250 1.0 3.0

26 19 15 8 -2.65550 1.0 1.0

26 19 15 8 -0.22350 -1.0 2.0

26 19 15 8 0.24150 1.0 3.0

34 26 19 15 3.69350 1.0 1.0

34 26 19 15 -1.82950 -1.0 2.0

34 26 19 20 0.23400 1.0 3.0

34 26 19 21 0.23400 1.0 3.0

19 26 34 35 -0.11600 1.0 1.0

19 26 34 35 -0.09450 -1.0 2.0

19 26 34 35 0.21250 1.0 3.0

19 15 8 9 0.00000 1.0 1.0

19 15 8 10 0.00000 1.0 1.0

19 15 8 6 0.05050 1.0 1.0

19 15 8 6 -1.30700 -1.0 2.0

19 15 8 6 0.91550 1.0 3.0

15 8 6 1 0.15000 1.0 3.0

9 8 6 1 0.50000 1.0 3.0

10 8 6 1 0.50000 1.0 3.0

6 1 3 4 -0.22900 1.0 1.0

6 1 3 4 0.09450 -1.0 2.0

6 1 3 4 -0.07800 1.0 3.0

6 1 5 7 0.91800 1.0 1.0

6 1 5 7 0.22900 -1.0 2.0

6 1 5 7 0.32050 1.0 3.0

11 7 13 16 0.23400 1.0 3.0

11 7 13 17 0.15000 1.0 3.0

11 7 13 14 0.15000 1.0 3.0

12 7 13 16 0.23400 1.0 3.0

12 7 13 17 0.15000 1.0 3.0

12 7 13 14 0.15000 1.0 3.0

14 13 16 30 0.37050 1.0 3.0

14 13 17 22 0.23400 1.0 3.0

14 13 17 24 0.15000 1.0 3.0

14 13 17 18 0.15000 1.0 3.0

18 17 22 23 0.19450 1.0 3.0

18 17 24 28 0.23400 1.0 3.0

18 17 24 30 0.15000 1.0 3.0

18 17 24 25 0.15000 1.0 3.0

25 24 28 29 0.19450 1.0 3.0

25 24 30 16 0.23400 1.0 3.0

25 24 30 36 0.15000 1.0 3.0

25 24 30 31 0.15000 1.0 3.0

31 30 36 41 0.00000 1.0 1.0

31 30 36 68 0.00000 1.0 1.0

42 41 48 50 3.62500 -1.0 2.0

62 61 65 68 3.62500 -1.0 2.0

64 63 66 67 2.45000 -1.0 2.0

64 63 66 69 2.56700 -1.0 2.0

73 72 74 75 3.62500 -1.0 2.0

73 72 74 79 3.62500 -1.0 2.0

84 83 51 71 3.62500 -1.0 2.0

84 83 51 49 3.62500 -1.0 2.0

44 43 37 46 0.23400 1.0 3.0

44 43 37 32 0.15000 1.0 3.0

44 43 37 38 0.15000 1.0 3.0

45 43 37 46 0.23400 1.0 3.0

45 43 37 32 0.15000 1.0 3.0

45 43 37 38 0.15000 1.0 3.0

38 37 46 47 0.19450 1.0 3.0

38 37 32 39 0.23400 1.0 3.0

38 37 32 26 0.15000 1.0 3.0

38 37 32 33 0.15000 1.0 3.0

33 32 39 40 0.19450 1.0 3.0

33 32 26 34 0.23400 1.0 3.0

33 32 26 19 0.15000 1.0 3.0

33 32 26 27 0.15000 1.0 3.0

27 26 34 35 0.19450 1.0 3.0

27 26 19 15 0.23400 1.0 3.0

27 26 19 20 0.15000 1.0 3.0

27 26 19 21 0.15000 1.0 3.0

20 19 15 8 0.03000 1.0 3.0

21 19 15 8 0.03000 1.0 3.0

IPHI

41 68 36 30 4.00000 -1.0 2.0

48 42 41 36 1.10000 -1.0 2.0

53 68 50 48 4.00000 -1.0 2.0

55 56 53 50 4.00000 -1.0 2.0

60 53 55 59 1.00000 -1.0 2.0

65 62 61 56 1.10000 -1.0 2.0

50 65 68 36 4.00000 -1.0 2.0

54 63 57 58 10.50000 -1.0 2.0

64 57 63 66 1.00000 -1.0 2.0

63 69 66 67 10.50000 -1.0 2.0

66 52 69 70 10.50000 -1.0 2.0

72 51 71 70 4.00000 -1.0 2.0

74 73 72 71 1.10000 -1.0 2.0

75 79 74 72 4.00000 -1.0 2.0

80 83 79 74 4.00000 -1.0 2.0

51 84 83 79 1.10000 -1.0 2.0

83 49 51 71 4.00000 -1.0 2.0

43 52 49 51 1.00000 -1.0 2.0

49 69 52 54 10.50000 -1.0 2.0

END

* LIGAND DATABASE FILE (OPLS2005)

*

HIF 16 16 25 36 69

1 0 M N _N__ 1 1.32888 114.27491 147.12704

2 1 S H _H__ 14 1.01008 119.96957 0.05935

3 1 M CT1 _CA_ 2 1.44126 118.43521 -179.94405

4 3 S HC _HA_ 15 1.09026 105.09173 5.88783

5 3 M C _C__ 3 1.50445 111.47958 -107.43472

6 5 S O _O__ 4 1.23381 120.25866 -28.32038

7 3 S CT _CB_ 5 1.54182 112.46574 119.70006

8 7 S HC _HB2 11 1.09044 109.23908 -166.53273

9 7 S HC _HB3 16 1.09021 109.22708 -55.00168

10 7 S CA5 _CG_ 6 1.50095 116.36422 69.22096

11 10 S N5B _ND1 7 1.37689 124.23761 -32.92503

12 10 S CA5 _CD2 8 1.35510 129.73426 154.69055

13 12 S HA _HD2 12 1.08036 120.02487 -4.01610

14 11 S CA5 _CE1 9 1.32338 109.45786 -176.74418

15 14 S HA _HE1 13 1.08025 119.96810 -177.99904

16 12 S NB _NE2 10 1.37223 107.68894 175.99337

9 4 9 6 5 1 8 4 3 6 5 4 2 2 1 1

3 5 6 7 10 8 2 4 9

4 3 5 7

5 6 7 10 11 12 8 4 9

9 5 6 7 8 10

6 7 10 8 9

7

10 11 12 14 16 8 13 9

9 10 11 12

10 11 12

11 12 14 16 13 15

12 14 16 13 15

14 16 13 15

14 16

16 15

16

0

NBON

1 3.2500 0.1700 -0.500000 1.8100 1.6000 0.054801302 -1.445846770

2 0.5000 0.0300 0.300000 1.5900 1.4000 0.030040813 -0.651083722

3 3.5000 0.0660 0.140000 1.9750 1.7500 0.005000000 -0.741685710

4 2.5000 0.0300 0.060000 1.3810 1.2100 0.008598240 0.268726247

5 3.7500 0.1050 0.500000 1.9250 1.7000 0.001000000 -0.126889456

6 2.9600 0.2100 -0.500000 1.5250 1.4250 0.001000000 -1.480495490

7 3.5000 0.0660 -0.297000 2.4950 1.7500 0.005000000 -0.741685710

8 2.5000 0.0300 0.060000 1.3810 1.2100 0.008598240 0.268726247

9 2.5000 0.0300 0.060000 1.3810 1.2100 0.008598240 0.268726247

10 3.5500 0.0700 0.504000 2.0020 1.7750 0.001000000 -0.843144165

11 3.2500 0.1700 -0.564000 1.8650 1.6500 0.001000000 -0.511443539

12 3.5500 0.0700 -0.261000 2.0020 1.7750 0.001000000 -0.843144165

13 2.4200 0.0300 0.183000 1.3810 1.2100 0.030040813 0.268726247

14 3.5500 0.0700 0.182000 2.0020 1.7750 0.001000000 -0.843144165

15 2.4200 0.0300 0.098000 1.3810 1.2100 0.030040813 0.268726247

16 1.8500 0.1700 0.035000 1.8375 1.6250 0.005000000 0.000000000

BOND

1 3 337.000 1.449

1 2 434.000 1.010

3 5 317.000 1.522

3 7 268.000 1.529

3 4 340.000 1.090

5 6 570.000 1.229

7 10 317.000 1.510

7 8 340.000 1.090

7 9 340.000 1.090

10 11 525.000 1.333

10 12 360.000 1.432

11 14 525.000 1.333

12 16 470.000 1.355

12 13 385.000 1.083

14 16 470.000 1.355

14 15 385.000 1.083

THET

1 3 5 63.00000 110.10000

1 3 7 80.00000 109.70000

1 3 4 35.00000 109.50000

3 5 6 80.00000 120.40000

3 7 10 63.00000 114.00000

3 7 8 37.50000 110.70000

3 7 9 37.50000 110.70000

7 3 5 63.00000 111.10000

7 10 11 60.00000 130.00000

7 10 12 65.00000 117.00000

10 11 14 90.00000 108.12000

10 12 16 70.00000 118.32000

10 12 13 35.00000 128.00000

11 14 16 70.00000 120.00000

11 14 15 40.00000 123.38000

12 10 11 80.00000 112.94000

12 16 14 70.00000 125.10000

8 7 10 35.00000 109.50000

13 12 16 35.00000 116.00000

15 14 16 35.00000 116.00000

2 1 3 38.00000 118.40000

4 3 5 35.00000 109.50000

4 3 7 37.50000 110.70000

9 7 10 35.00000 109.50000

9 7 8 33.00000 107.80000

PHI

1 3 5 6 0.00000 1.0 1.0

1 3 7 10 0.42250 1.0 1.0

1 3 7 10 -0.48100 -1.0 2.0

1 3 7 10 0.35650 1.0 3.0

1 3 7 8 0.23200 1.0 3.0

1 3 7 9 0.23200 1.0 3.0

3 7 10 11 0.29300 1.0 1.0

3 7 10 11 -1.19700 -1.0 2.0

3 7 10 11 0.60900 1.0 3.0

3 7 10 12 0.00000 1.0 1.0

5 3 7 10 -0.84850 1.0 1.0

5 3 7 10 -0.22800 -1.0 2.0

5 3 7 10 0.29250 1.0 3.0

5 3 7 8 -0.03800 1.0 3.0

5 3 7 9 -0.03800 1.0 3.0

7 3 5 6 0.00000 1.0 1.0

7 10 11 14 3.62500 -1.0 2.0

7 10 12 16 3.62500 -1.0 2.0

7 10 12 13 3.62500 -1.0 2.0

10 11 -14 16 3.62500 -1.0 2.0

10 11 14 15 3.62500 -1.0 2.0

10 12 -16 14 3.62500 -1.0 2.0

11 10 -12 16 3.62500 -1.0 2.0

11 10 12 13 3.62500 -1.0 2.0

11 14 -16 12 3.62500 -1.0 2.0

12 10 -11 14 3.62500 -1.0 2.0

8 7 10 11 0.00000 1.0 1.0

8 7 10 12 0.00000 1.0 1.0

13 12 16 14 3.62500 -1.0 2.0

15 14 16 12 3.62500 -1.0 2.0

2 1 3 5 0.00000 1.0 1.0

2 1 3 7 0.00000 1.0 1.0

2 1 3 4 0.00000 1.0 1.0

4 3 5 6 0.00000 1.0 1.0

4 3 7 10 0.23100 1.0 3.0

4 3 7 8 0.15000 1.0 3.0

4 3 7 9 0.15000 1.0 3.0

9 7 10 11 0.00000 1.0 1.0

9 7 10 12 0.00000 1.0 1.0

IPHI

11 12 10 7 4.00000 -1.0 2.0

16 13 12 10 1.10000 -1.0 2.0

16 15 14 11 1.10000 -1.0 2.0

END
